# A quadruple cascade protocol for the one-pot synthesis of fully-substituted hexahydroisoindolinones from simple substrates

**DOI:** 10.3762/bjoc.12.27

**Published:** 2016-02-11

**Authors:** Hong-Bo Zhang, Yong-Chun Luo, Xiu-Qin Hu, Yong-Min Liang, Peng-Fei Xu

**Affiliations:** 1 State Key Laboratory of Applied Organic Chemistry, College of Chemistry and Chemical Engineering Lanzhou University, Lanzhou 730000, P. R. China

**Keywords:** bifunctional catalysis, hexahydroisoindolinones, one-pot synthesis, quadruple cascade

## Abstract

A new and efficient synthetic method to obtain fully-substituted hexahydroisoindolinones was developed by using bifunctional tertiary amine-thioureas as powerful catalysts. As far as we know, there is no efficient synthetic method developed toward fully-substituted hexahydroisoindolinones. The products were obtained in good yield and diastereoselectivity. The one-pot cascade quadruple protocol features readily available starting materials, simple manipulation, mild conditions and good atom economy.

## Introduction

Isoindolines and their congeners are one kind of the most widespread compounds in nature. They feature not only high biological activity, but also diverse chemical properties [1–16]. Therefore, it is highly desirable to develop efficient methods toward the synthesis of isoindoline derivatives, which is a frontier in organic synthesis.

However, compared with the synthesis of their congeners, the synthesis of fully-substituted hexahydroisoindolinones is much more difficult due to the steric hindrance and the high strain of the molecular architectures [17]. Three methods to synthesize 3-substituted isoindolinones have been developed. The first method was the synthesis of 3-substituted isoindolinones from the corresponding *N*-methylmaleimides by the Diels–Alder reaction with 1,3-butadiene followed by hydrogenation. The second and the third methods employed the corresponding dicarboxylic acids and the carboxylic acid anhydrides, respectively [17]. To the best of our knowledge, no efficient method toward the synthesis of fully-substituted hexahydroisoindolinones has been developed so far.

The synthesis of complicated molecular structures can now be achieved by organocatalytic cascade reactions [18–33]. By simplifying the experimental procedures and reducing the usage of both solvents and reagents, one-pot reactions can improve the synthesis efficiency and both save time and reduce cost [34]. Although a few types of complicated molecules were generated through multicomponent quadruple cascade reactions, there is no report about the cascade synthesis of isoindolines in the past few decades [35–46], not mention the quadruple cascade synthesis of difficult fully-substituted hexahydroisoindolinones. Previously, we established organocatalytic domino reactions to construct very useful molecular architectures [47–60]. Based on this past experience, we decided to develop a one-pot quadruple protocol to construct this difficult molecular architecture using easily accessible substrates.

## Results and Discussion

We initiated this study by using 2-benzylidenemalononitrile (**1a**) and 2-oxo-*N*,3-diphenylpropanamide (**2a**) [61–64] in 0.5 mL of CH_3_CN in the presence of 10 mol % of DABCO. After 12 h at room temperature, the reaction afforded the expected product ***rac*-3a** in 59% yield (Table 1, entry 1). We then tested different catalysts to optimize the reaction. When Et_3_N was used, the reaction afforded the product with 41% yield (Table 1, entry 2). However, a complex mixture was observed when DBU was used (Table 1, entry 3), while no reaction was observed when K_2_CO_3_ was used as the catalyst (Table 1, entry 4). When thioureas were used as the catalysts, we also did not get the expected product (Table 1, entries 5 and 6). Since bifunctional tertiary amine-thioureas have been proved as powerful catalysts that can catalyze a variety of organocascade reactions, we also tested thiourea catalysts, **cat-1** to **cat-3**. Interestingly, the thioureas **cat-1** and **cat-2** were able to promote the reaction (Table 1, entries 7 and 8), but we obtained an even better yield when the tertiary amine-thiourea **cat-3** was used as the catalyst (Table 1, entry 9). All products were racemic even when chiral catalysts were used (see Supporting Information File 1 for details). Next, we performed a solvent screening. As shown in Table 1, when DCM and THF were used as the solvent, the yield of the desired product was 33% and 34%, respectively (Table 1, entries 10 and 11). Only traces of the product were seen when toluene or methanol was used as the solvent (Table 1, entries 12 and 13). Furthermore, raising the reaction temperature was not beneficial for the diastereoselectivity of the reaction (Table 1, entry 14).

**Table 1:** Screening the reaction conditions.^a^

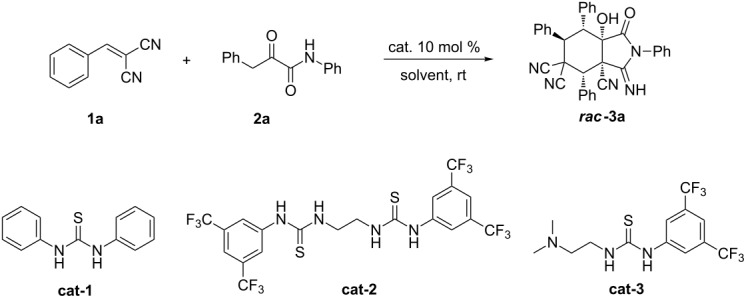				

entry	cat.	solvent	dr^b^	yield [%]^c^

1	DABCO	CH_3_CN	4:1	59
2	Et_3_N	CH_3_CN	4:1	41
3	DBU	CH_3_CN	n.d.	complex
4	K_2_CO_3_	CH_3_CN	n.d.	n.r.
5	**cat-1**	CH_3_CN	n.d.	n.r.
6	**cat-2**	CH_3_CN	n.d.	n.r.
7	DABCO^d^	CH_3_CN	4:1	62
8	Et_3_N^d^	CH_3_CN	5:1	52
9	**cat-3**	CH_3_CN	9:1	87
10	**cat-3**	DCM	4:1	33
11	**cat-3**	THF	4:1	34
12	**cat-3**	toluene	n.d.	trace
13	**cat-3**	CH_3_OH	n.d.	trace
14^e^	**cat-3**	CH_3_CN	6:1	87

^a^Unless otherwise noted, the reactions were carried out with **1a** (0.25 mmol, 38.5 mg), **2a** (0.1 mmol, 23.9 mg), catalyst (0.01 mmol, 10 mol %) in the indicated solvent (0.5 mL) at rt for 12 h. ^b^Determined by ^1^H NMR analysis of the crude products. ^c^Column chromatography yields. ^d^10 mol % **cat-2** was added. ^e^The reaction was carried out at 35 °C.

With the optimal conditions in hand, we next examined the reaction scope (Table 2). All reactions afforded the corresponding products **3a**–**t** with medium to good yield and diastereoselectivity using the simple protocol at room temperature. To our delight, with our optimized reaction system, various types of substrates **1** showed very good reaction activities. Different types of substrates **1**, bearing either electron withdrawing or donating groups in *para-*, *meta-* and *ortho-*positions, gave the desired products in good yield and diastereoselectivity (Table 2, entries 1–10 and 12), although 4-NO_2_C_6_H_4_ gave the product in medium yield due to its poor solubility (Table 2, entry 11). A heteroaromatic substrate such as thiophene could also be successfully employed to afford ***rac-*3** with medium yield and diastereoselectivity (Table 2, entry 13). 3,4-Dichloro-substituted and 3,5-dimethoxy-substituted substrates produced the desired products in 84% and 55% yield with 20:1 and 15:1 diastereoselectivity, respectively (Table 2, entries 14 and 15). When substrates with different R^2^ and R^3^ were used in this reaction, the corresponding products were obtained in medium yield and diastereoselectivity (Table 2, entries 16–20). The structure of **3p** was determined by X-ray analysis [65]. However, substrates with aliphatic R^1^, R^2^ or R^3^ did not produce the desired products (Table 2, entries 21–26).

**Table 2:** Substrates scope.^a^

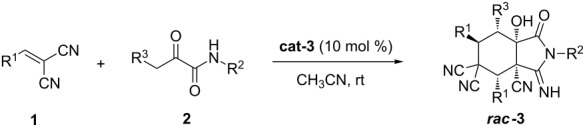					

entry	R^1^	R^2^	R^3^	dr^b^	yield [%]^c^

1	C_6_H_5_	C_6_H_5_	C_6_H_5_	9:1	87 (**3a**)
2	2-MeC_6_H_4_	C_6_H_5_	C_6_H_5_	>20:1	89 (**3b**)
3	3-MeC_6_H_4_	C_6_H_5_	C_6_H_5_	10:1	69 (**3c**)
4	4-OMeC_6_H_4_	C_6_H_5_	C_6_H_5_	10:1	66 (**3d**)
5	2-BrC_6_H_4_	C_6_H_5_	C_6_H_5_	>20:1	84 (**3e**)
6	3-ClC_6_H_4_	C_6_H_5_	C_6_H_5_	4:1	72 (**3f**)
7	4-FC_6_H_4_	C_6_H_5_	C_6_H_5_	>20:1	82 (**3g**)
8	4-CF_3_C_6_H_4_	C_6_H_5_	C_6_H_5_	>20:1	86 (**3h**)
9	2-NO_2_C_6_H_4_	C_6_H_5_	C_6_H_5_	>20:1	89 (**3i**)
10	3-NO_2_C_6_H_4_	C_6_H_5_	C_6_H_5_	>20:1	91 (**3j**)
11	4-NO_2_C_6_H_4_	C_6_H_5_	C_6_H_5_	3:1	42 (**3k**)
12	2-naphthalene	C_6_H_5_	C_6_H_5_	>20:1	90 (**3l**)
13	2-thiophene	C_6_H_5_	C_6_H_5_	3:1	51 (**3m**)
14	3,4-diClC_6_H_3_	C_6_H_5_	C_6_H_5_	>20:1	84 (**3n**)
15	3,5-diOMeC_6_H_3_	C_6_H_5_	C_6_H_5_	15:1	55 (**3o**)
16	C_6_H_5_	4-OMeC_6_H_4_	C_6_H_5_	4:1	56 (**3p**)
17	C_6_H_5_	4-ClC_6_H_4_	C_6_H_5_	>20:1	89 (**3q**)
18	2-naphthalene	4-OMeC_6_H_4_	C_6_H_5_	14:1	88 (**3r**)
19	C_6_H_5_	C_6_H_5_	4-MeC_6_H_4_	8:1	61 (**3s**)
20	C_6_H_5_	C_6_H_5_	4-FC_6_H_4_	8:1	61 (**3t**)
21	C_6_H_5_(CH_2_)_2_	C_6_H_5_	C_6_H_5_	n.d.	n.r.
22	CH_3_(CH_2_)_5_	C_6_H_5_	C_6_H_5_	n.d.	n.r.
23	C_6_H_5_	CH_3_(CH_2_)_3_	C_6_H_5_	n.d.	n.r.
24	C_6_H_5_	CH_3_CH_2_	C_6_H_5_	n.d.	n.r.
25	C_6_H_5_	C_6_H_5_	H	n.d.	n.r.
26	C_6_H_5_	C_6_H_5_	CH_3_	n.d.	n.r.

^a^Unless otherwise noted, the reactions were carried out with **1** (0.25 mmol), **2** (0.1 mmol), **cat-3** (3.6 mg, 0.01 mmol, 10 mol %) in CH_3_CN (0.5 mL) at rt for 12 h. ^b^Determined by ^1^H NMR analysis of the crude products. ^c^Column chromatography yields.

**Scheme 1:**
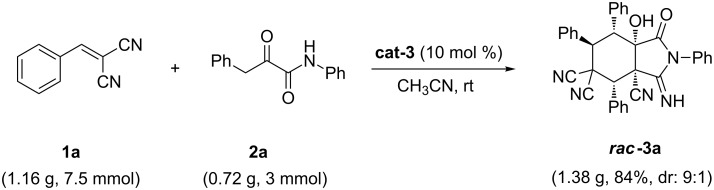
An example of scalable synthesis.

This bifunctional catalysis cascade reaction was also amenable to scale-up. When the reaction was carried out on a 3 mmol scale, the desired product was obtained in 84% yield. Therefore, this method is fast and easy to implement, and it is suitable for large-scale synthesis (Scheme 1).

**Scheme 2:**
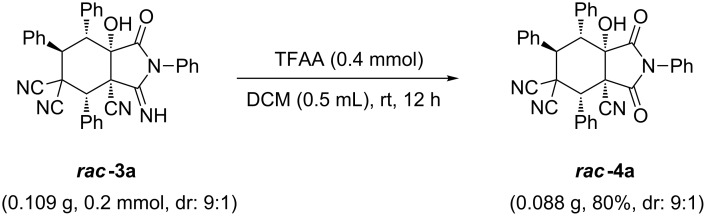
Hydrolysis reaction to produce a useful product.

Many isoindolinone skeletons show high biological potential as antihypertensives, anesthetics, etc. [66–68]. The useful hydrolyzed product ***rac-*4a** was obtained in 80% yield by treating ***rac-*3a** with trifluoroacetic anhydride in DCM (Scheme 2).

**Scheme 3:**
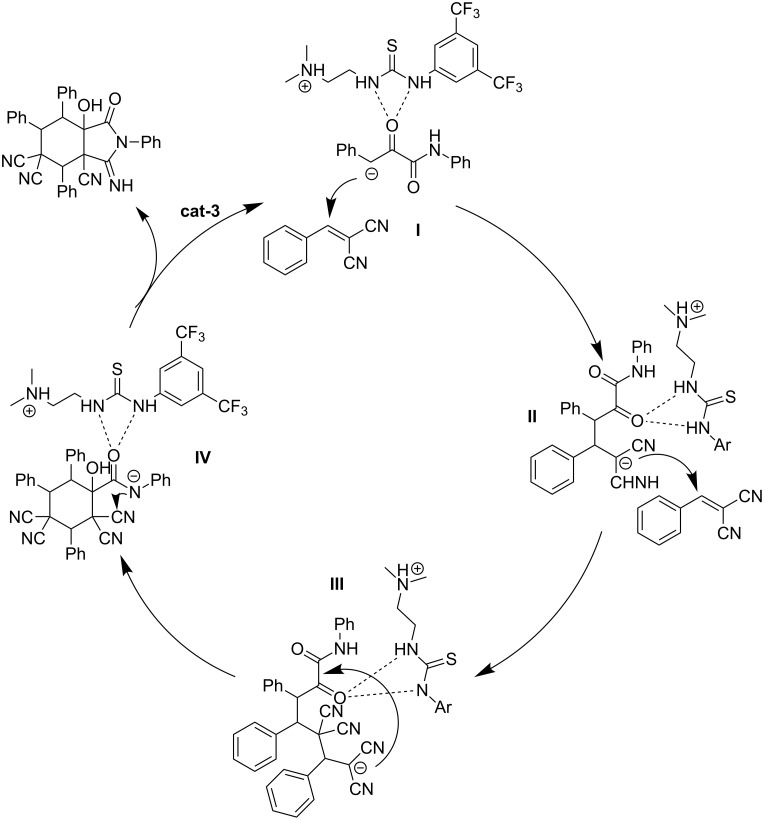
Proposed mechanism.

Finally, we propose a mechanism for the reaction. Initially, substrate **1** is activated by catalyst (**I**), which reacts with substrate **2** via two Michael addition reactions to sequentially produce **II** and **III**. Then, **IV** is generated from **III** by an aldol reaction. Finally, the product is produced after the nucleophilic reaction, and the catalyst is regenerated (Scheme 3).

## Conclusion

In summary, we have developed a one-pot quadruple cascade protocol to obtain fully-substituted hexahydroisoindolinones. This new, synthetic method is simple, efficient and atom-economic. This reaction can be widely used in organic synthesis due to its advantages such as simple operation, availability of raw materials, mild conditions and high efficiency.

## Experimental

### General procedure for the synthesis of fully-substituted hexahydroisoindolinones

Benzylidenemalononitrile (0.1 mmol), 2-oxo-*N*,3-diphenylpropanamide (0.25 mmol) and **cat-3** (0.01 mmol) were added to a test tube, then CH_3_CN (0.5 mL) was added to the mixture. The reaction mixture was stirred at 300 rpm at 21 °C in a stoppered carousel tube for 12 h. The solvent was removed in vacuo and the product was purified by silica gel flash column chromatography to give the corresponding product **3**.

## Supporting Information

File 1 Experimental procedures, characterization data for all new compounds and X-ray analysis of compound **3**.
