# Supplementary material for: A quadruple cascade protocol for the one-pot synthesis of fully-substituted hexahydroisoindolinones from simple substrates
Source: Beilstein J Org Chem. 2016 Feb 11;12:253–9. doi: 10.3762/bjoc.12.27 (PMC4778526; doi:10.3762/bjoc.12.27)
Supplement: File 1 — Experimental procedures, characterization data for all new compounds and X-ray analysis of compound 3. [file Beilstein_J_Org_Chem-12-253-s001.pdf]

Supporting Information

for

A quadruple cascade protocol for the one-pot synthesis of fully substituted hexahydroisoindolinones from simple substrates

Hong-Bo Zhang, Yong-Chun Luo, Xiu-Qin Hu, Yong-Min Liang and Peng-Fei Xu*

Address: State Key Laboratory of Applied Organic Chemistry, College of Chemistry and Chemical Engineering Lanzhou University, Lanzhou 730000, P. R. China

Email: Peng-Fei Xu - xupf@lzu.edu.cn

* Corresponding author

Experimental procedures, characterisation data for all new compounds and X-ray analysis of compound 3

Contents

1. General information S2
2. General procedure for the preparation of compounds **3a–t**, **4a** S2
3. Screening the reaction conditions using chiral catalysts S2–S3
4. Analytical data S4–S17
5. NMR spectra S18–S59
6. XRD spectra S60–S61

General information

Chemicals and solvents were either purchased from commercial suppliers or purified by standard procedures as specified in *Purification of Laboratory Chemicals*, 4th Ed (Armarego, W. L. F.; Perrin, D. D. Butterworth Heinemann: 1997). All reactions were carried out in carousel tubes (15 cm × 2 cm) equipped with an octagon-shaped magnetic stirrer bar (12.7 mm × 3 mm). All reactions were monitored by thin-layer chromatography (TLC) on pre-coated silica gel plates (254 μm). Flash column chromatography was carried out with (200–300 mesh) silica gel. ^{1}H NMR spectra were recorded at 400 MHz and ^{13}C NMR at 100 MHz on Bruker AM-400 spectrometers at ambient temperature. HRMS were performed on Bruker maXis 4G mass instrument (ESI) or Thermo ORBITRAP ELITE (ESI). IR spectra were recorded using Nicolet NEXUS 670 FT-IR instrument.

α-Ketoamides preparation

The 1,2-ketoacids were prepared by the method which has been reported.[1,2] The Damien Bonne and Jean Rodriguez's method was used to prepare the α-ketoamides.[3]

General Procedure for the functionalization of benzylidenemalononitrile with 2-oxo-N,3-diphenylpropanamide

Benzylidenemalononitrile (0.1 mmol), 2-oxo-*N*,3-diphenylpropanamide (0.25 mmol) and **cat-3** (0.01 mmol) were added to a test tube, then CH_3CN (0.5 ml) was added to the mixture. The reaction mixture was stirred at 300 rpm at 21 °C in a stoppered carousel tube for 12 h. The solvent was removed in vacuo and the product was purified as specified below.

Preparation procedure for the hydrolyzed product *rac*-4a

7a-Hydroxy-3-imino-1-oxo-2,4,6,7-tetraphenylhexahydro-5*H*-isoindole-3a,5,5(4*H*)-tricarbonitrile (***rac*-3a**, 0.109 g, 0.2 mmol) and CH_2Cl_2 (0.5 mL) were added to a test tube, then trifluoroacetic anhydride (28 μL, 0.4 mmol) was added to the mixture. The reaction mixture was stirred at 300 rpm at 21 °C in a stoppered carousel tube for 12 h. The solvent was removed in vacuo and the product was purified as specified below.

Optimization of the reaction conditions using chiral catalysts

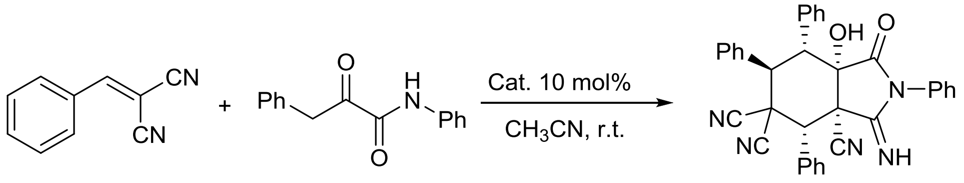

1a **2a** **3a**

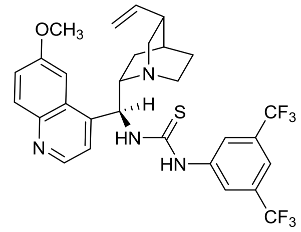

Cat. 4

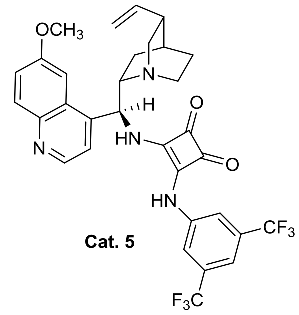

Cat. 5

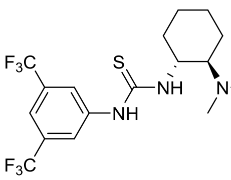

Cat. 6

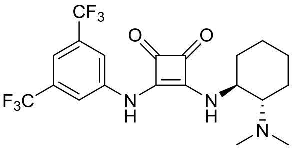

Cat. 7

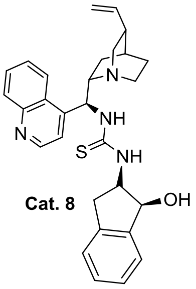

Cat. 8

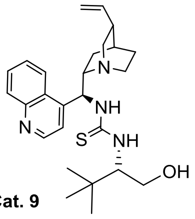

Cat. 9

Table S1: The optimization of the reaction conditions using chiral catalysts.[a]

**entry**	**cat.**	**d.r. [b]**	**yield [c]**	***ee* [d] %**
**1**	4	8:1	85 %	*rac.*
**2**	5	5:1	62 %	*rac.*
**3**	6	9:1	80 %	*rac.*
**4**	7	4:1	65 %	*rac.*
**5**	8	6:1	77 %	*rac.*
**6**	9	6:1	76 %	*rac.*

[a]Unless otherwise noted, the reactions were carried out with **1a** (0.25 mmol, 38.5 mg), **2a** (0.1 mmol, 23.9 mg), catalyst (0.01 mmol, 10 mol %) in the CH_3CN (0.5 mL) at rt for 12 h. [b]Determined by ^{1}H NMR analysis. [c]Column chromatography yields. [d]Determined by chiral-phase HPLC analysis.

Different types of chiral catalysts were tested, but all the products were racemic. Therefore, racemic catalysts were used in all the reactions.

7a-Hydroxy-3-imino-1-oxo-2,4,6,7-tetraphenylhexahydro-5*H*-isoindole-3a,5,5(4*H*)-tricarbonitrile (*rac*-3a, major)

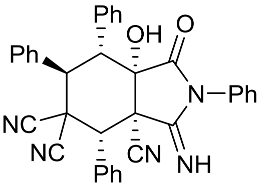

The reaction was stirred for 12 h. Purification by column chromatography (25% EtOAc/Petrol) gave 7a-hydroxy-3-imino-1-oxo-2,4,6,7-tetraphenylhexahydro-5*H*-isoindole-3a,5,5(4*H*)-tricarbonitrile as a white solid (47.5 mg, 0.087 mmol, 87%): m.p.: 194-199 °C. ^{1}H NMR (400 MHz, $CDCl_3$) δ 8.57 (s, 1H), 7.64–7.58 (m, 6H), 7.39-7.37 (d, 3H), 7.17–7.02 (m, 11H), 5.26 (s, 1H), 4.27 (d, *J* = 12.0 Hz, 1H), 3.98 (d, *J* = 12.0 Hz, 1H), 3.85 (s, 1H); ^{13}C NMR (100 MHz, $CDCl_3$) δ 171.6, 155.2, 133.5, 132.9, 131.9, 130.7, 130.5, 130.4, 129.5, 129.0, 128.7, 128.6, 128.4, 126.7, 114.6, 114.0, 112.3, 77.2, 52.4, 49.5, 47.7, 45.9, 42.5; IR (thin film) 3366, 3274, 3066, 2950, 2261, 1768, 1678, 1596, 1494, 1457, 1393 cm^{-1}; HRMS (ESI) calculated for $C_{35}H_{25}N_5O_2H$ $[M+H]^+$ 548.2081, found 548.2085.

7a-Hydroxy-3-imino-1-oxo-2,7-diphenyl-4,6-di-*o*-tolylhexahydro-5*H*-isoindole-3a, 5,5(4*H*)-tricarbonitrile (*rac*-3b, major)

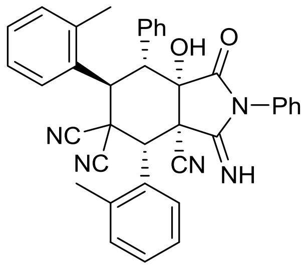

The reaction was stirred for 12 h. Purification by column chromatography (25% EtOAc/Petrol) gave 7a-hydroxy-3-imino-1-oxo-2,7-diphenyl-4,6-di-*o*-tolyl-hexa-hydro-5*H*-isoindole-3a,5,5(4*H*)-tricarbonitrile as a white solid (51.2 mg, 0.089 mmol, 89%): m.p.: 196-201 °C. ^{1}H NMR (400 MHz, CD_3CN) δ 8.95 (s, 1H),8.47–

8.45 (d, 1H), 7.69–7.38 (m, 10H), 7.18 (s, 4H), 7.08-7.03 (m, 3H), 5.65 (s, 1H), 5.28 (s, 1H), 4.84 (d, *J* = 12.0 Hz, 1H), 4.03 (d, *J* = 12.0 Hz, 1H), 2.61 (s, 3H), 2.29 (s, 3H); ^{13}C NMR (100 MHz, CD_3CN) δ 172.3, 156.2, 140.0, 139.1, 134.8, 134.3, 133.9, 132.5, 132.1, 131.7, 131.4, 131.2, 131.0,130.7, 129.5, 129.0, 128.9, 128.3, 128.2,127.0, 116.5, 115.0, 114.2, 79.1, 54.6, 49.9, 45.3, 42.1, 38.6, 21.1, 20.4; IR (thin film) 3435, 3281, 3068, 2970, 2261, 1770, 1683, 1493, 1458, 1388 cm^{-1}; HRMS (ESI) calculated for $C_{37}H_{29}N_5O_2H$ $[M+H]^+$ 576.2394, found 576.2389.

7a-Hydroxy-3-imino-1-oxo-2,7-diphenyl-4,6-di-*m*-tolylhexahydro-5*H*-isoindole-3a,5,5(4*H*)-tricarbonitrile (*rac*-3c, major)

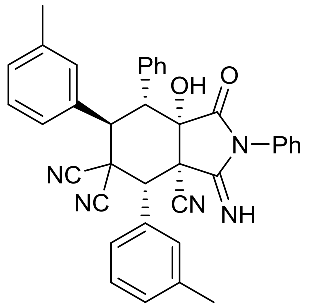

The reaction was stirred for 12 h. Purification by column chromatography (25% EtOAc/Petrol) gave 7a-hydroxy-3-imino-1-oxo-2,7-diphenyl-4,6-di-*m*-tolylhexa-hydro-5*H*-isoindole-3a,5,5(4*H*)-tricarbonitrile as a white solid (39.7 mg, 0.069 mmol, 69%): m.p.: 195-200 ^{o}C. ^{1}H NMR (400 MHz, $CDCl_3$) δ 8.56 (s, 1H), 7.64–7.39 (m, 8H), 7.18–6.85 (m, 10H), 5.22 (s, 1H), 4.23 (d, *J* = 12.0 Hz, 1H), 3.93 (d, *J* = 12.0 Hz, 1H), 3.61 (s, 1H), 2.48 (s, 3H), 2.17 (s, 3H); ^{13}C NMR (100 MHz, $CDCl_3$) δ 171.7, 155.2, 139.2, 138.2, 133.4, 132.8, 132.0, 131.2, 130.7, 130.4, 129.7, 129.4, 128.9, 128.6, 128.4,126.7, 114.6, 114.1, 112.3, 77.2, 52.4, 49.5, 47.6, 46.0, 42.3, 21.6, 21.2; IR (thin film) 3438, 3277, 3066, 2924, 2261, 1768, 1679, 1607, 1493, 1457, 1391 cm^{-1}; HRMS (ESI) calculated for $C_{37}H_{29}N_5O_2H$ $[M+H]^+$ 576.2394, found 576.2397.

7a-Hydroxy-3-imino-4,6-bis(4-methoxyphenyl)-1-oxo-2,7-diphenylhexahydro-5H-isoindole-3a,5,5(4*H*)-tricarbonitrile (*rac*-3d, major)

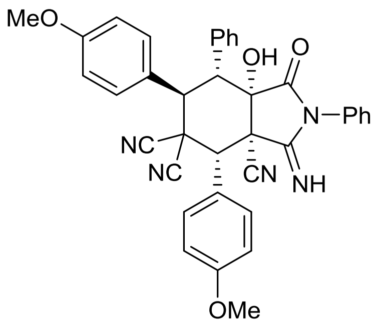

The reaction was stirred for 12 h. Purification by column chromatography (30% EtOAc/Petrol) gave 7a-hydroxy-3-imino-4,6-bis(4-methoxyphenyl)-1-oxo-2,7-diphenylhexahydro-5*H*-isoindole-3a,5,5(4*H*)-tricarbonitrile as a white solid (40.1 mg, 0.066 mmol, 66%): m.p.: 199-205 ^oC. ^1H NMR (400 MHz, d_6-DMSO) δ 8.63 (s, 1H), 7.58–7.05 (m, 18H), 5.26 (s, 1H), 5.12 (s, 1H), 4.35 (s, 1H), 4.31 (s, 1H), 3.64 (s, 6H); ^{13}C NMR (100 MHz, d_6-DMSO) δ 171.1, 160.1, 158.7, 134.6, 134.5, 133.1, 131.3, 129.8, 128.9, 127.7, 127.6, 126.7,126.6, 126.3, 126.2, 124.9, 116.1, 115.5, 114.4, 114.2, 113.9, 77.2, 55.2, 55.0, 54.9, 54.1, 53.1, 47.4, 46.9; IR (thin film) 3691, 3425, 2254, 2127, 1763, 1657, 1516, cm^{-1}; HRMS (ESI) calculated for $C_{37}H_{29}N_5O_4H$ $[M+H]^+$ 608.2292, found 608.2300.

4,6-Bis(2-bromophenyl)-7a-hydroxy-3-imino-1-oxo-2,7-diphenylhexahydro-5*H*-isoindole-3a,5,5(4*H*)-tricarbonitrile (*rac*-3e, major)

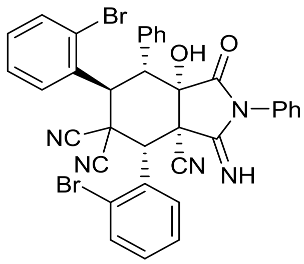

The reaction was stirred for 12 h. Purification by column chromatography (25% EtOAc/Petrol) gave 4,6-bis(2-bromophenyl)-7a-hydroxy-3-imino-1-oxo-2,7-diphenyl-hexahydro-5*H*-isoindole-3a,5,5(4*H*)-tricarbonitrile as a white solid (59.2 mg, 0.084 mmol, 84%): m.p.: 184-190 ^oC. ^1H NMR (400 MHz, CD_3CN) δ 9.01 (s, 1H), 8.66–8.65 (d, 1H), 8.64–8.63 (d, 1H), 7.89–7.62 (m, 5H), 7.54–7.38 (m, 6H), 7.23–7.18 (m, 5H), 6.20 (s, 1H), 5.43 (s, 1H), 5.17 (d, J = 12.0 Hz, 1H), 4.00 (d, J = 12.0 Hz, 1H);

^{13}C NMR (100 MHz, CD_3CN) δ 171.7, 155.0, 134.6, 134.3, 133.8, 133.7, 132.8, 132.7, 131.3, 131.2, 131.0, 130.7, 129.8, 128.9, 128.5, 128.2, 127.9, 115.9, 114.2, 112.9, 78.8, 55.0, 54.3, 49.6, 46.0, 45.5, 44.1; IR (thin film) 3412, 3279, 2928, 2376, 2263, 1763, 1678, 1596, 1534, 1473 cm^{-1}; HRMS (ESI) calculated for $C_{35}H_{23}Br_2N_5O_2H$ $[M+H]^+$ 706.0271, found 706.0278.

4,6-Bis(3-chlorophenyl)-7a-hydroxy-3-imino-1-oxo-2,7-diphenylhexahydro-5*H*-isoindole-3a,5,5(4*H*)-tricarbonitrile (*rac*-3f, major)

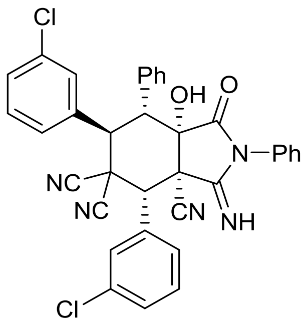

The reaction was stirred for 12 h. Purification by column chromatography (25% EtOAc/Petrol) gave 4,6-bis(3-chlorophenyl)-7a-hydroxy-3-imino-1-oxo-2,7-diphenylhexahydro-5*H*-isoindole-3a,5,5(4*H*)-tricarbonitrile as a white solid (44.3 mg, 0.072 mmol, 72%): m.p.: 179-184 °C. ^1H NMR (400 MHz, CD_3CN) δ 8.80 (s, 1H), 7.64–7.60 (m, 5H), 7.42–7.31 (m, 8H), 7.19 (br, 5H), 5.24 (s, 1H), 5.13 (s, 1H), 4.05 (d, J = 12.0 Hz, 1H), 4.02 (d, J = 12.0 Hz, 1H); ^{13}C NMR (100 MHz, CD_3CN) δ 171.6, 155.3, 137.1, 137.0, 135.2, 135.0, 134.4, 134.3, 133.7, 132.8, 131.8, 131.7, 131.6, 131.1, 131.0, 130.9, 130.8, 130.7, 130.6, 130.0, 129.6, 128.8, 128.7, 128.2, 128.0, 114.1, 114.0, 113.9, 79.8, 56.4, 53.7, 48.7, 46.5, 44.7; IR (thin film) 3631, 3440, 3275, 3068, 2261, 1770, 1682, 1596, 1574, 1493, 1481, 1456, 1437, 1388 cm^{-1}; HRMS (ESI) calculated for $C_{35}H_{15}Cl_2N_5O_2H$ $[M+H]^+$ 616.1302, found 616.1307.

4,6-Bis(4-fluorophenyl)-7a-hydroxy-3-imino-1-oxo-2,7-diphenylhexahydro-5*H*-isoindole-3a,5,5(4*H*)-tricarbonitrile (*rac*-3g, major)

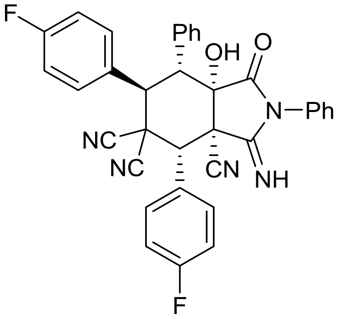

The reaction was stirred for 12 h. Purification by column chromatography (25% EtOAc/Petrol) gave 4,6-bis(4-fluorophenyl)-7a-hydroxy-3-imino-1-oxo-2,7-diphenylhexahydro-5*H*-isoindole-3a,5,5(4*H*)-tricarbonitrile as a white solid (47.8 mg, 0.082 mmol, 82%): m.p.: 176-182 ^{o}C. ^{1}H NMR (400 MHz, CD_3CN) δ 9.04 (s, 1H), 8.37–8.34 (q, 2H), 7.69–7.61 (m, 4H), 7.54–7.51 (q, 2H), 7.43–7.20 (m, 10H), 5.29 (br, 1H), 4.60 (d, *J* = 12.0 Hz, 1H), 4.46 (s, 1H), 4.01 (d, *J* = 12.0 Hz, 1H); ^{13}C NMR (100 MHz, CD_3CN) δ 171.2, 153.3, 135.4, 135.3, 134.5, 132.6, 131.4, 131.1, 130.8, 129.9, 129.4, 128.7, 128.6, 128.5, 128.2, 127.9, 116.5, 116.4, 116.0, 115.8, 115.7, 115.6, 79.8, 56.6, 48.9, 48.5, 48.4, 46.6; IR (thin film) 3437, 3274, 2263, 1770, 1687, 1606, 1513, 1493, 1383 cm^{-1}; HRMS (ESI) calculated for $C_{35}H_{23}F_2N_5O_2H$ $[M+H]^+$ 584.1893, found 584.1889.

7a-Hydroxy-3-imino-1-oxo-2,7-diphenyl-4,6-bis(4-(trifluoromethyl)phenyl)hexa-hydro-5*H*-isoindole-3a,5,5(4*H*)-tricarbonitrile (*rac*-3h, major)

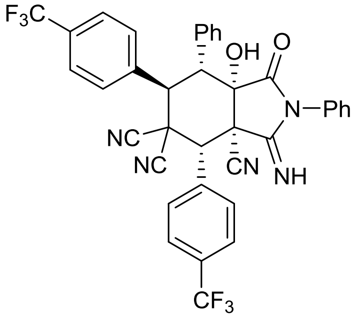

The reaction was stirred for 12 h. Purification by column chromatography (20% EtOAc/Petrol) gave 7a-hydroxy-3-imino-1-oxo-2,7-diphenyl-4,6-bis(4-(trifluoro-methyl)phenyl)hexahydro-5*H*-isoindole-3a,5,5(4*H*)-tricarbonitrile as a white solid (58.7 mg, 0.086 mmol, 86%): m.p.: 177-182 ^{o}C. ^{1}H NMR (400 MHz, CD_3CN) δ 8.79

(s, 1H), 7.88–7.86 (d, 1H), 7.71–7.57 (m, 7H), 7.42–7.19 (m, 10H), 5.24 (s, 1H), 4.71 (d, *J* = 12.0 Hz, 1H), 4.58 (s, 1H), 4.14 (d, *J* = 12.0 Hz, 1H); ^{13}C NMR (100 MHz, CD_3CN) δ 171.5, 155.3, 139.3, 138.9, 137.4, 134.2, 133.9, 133.6, 132.7, 131.7, 131.3, 131.1, 130.9, 130.8, 130.0, 128.9, 128.8, 128.6, 128.1, 127.9, 126.8, 126.7, 126.1, 126.0, 125.8, 113.9, 113.8, 113.8, 78.3, 53.6, 48.8, 48.7, 47.7, 46.6; IR (thin film) 3363, 3273, 3072, 2935, 2262, 1771, 1686, 1620, 1596, 1493, 1426, 1386 cm^{-1}; HRMS (ESI) calculated for $C_{37}H_{23}F_6N_5O_2H$ $[M+H]^+$ 684.1829, found 684.1824.

7a-Hydroxy-3-imino-4,6-bis(2-nitrophenyl)-1-oxo-2,7-diphenylhexahydro-5*H*-isoindole-3a,5,5(4*H*)-tricarbonitrile (*rac*-3i, major)

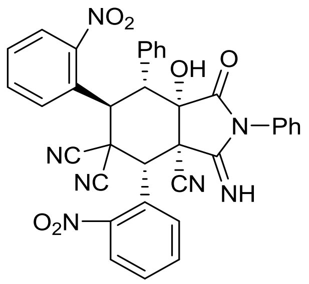

The reaction was stirred for 12 h. Purification by column chromatography (30% EtOAc/Petrol) gave 7a-hydroxy-3-imino-4,6-bis(2-nitrophenyl)-1-oxo-2,7-diphenylhexahydro-5*H*-isoindole-3a,5,5(4*H*)-tricarbonitrile as a yellow solid (56.6 mg, 0.089 mmol, 89%): m.p.: 199-205 ^{o}C. ^{1}H NMR (400 MHz, CD_3CN) δ 8.78–8.76 (d, 1H), 8.66 (s, 1H), 8.08–8.06 (d, 1H), 8.06–8.00 (d, 2H), 7.98–7.69 (m, 3H), 7.60–7.19 (m, 11H), 5.84 (s, 1H), 5.35 (d, *J* = 12.0 Hz, 1H), 4.05 (d, *J* = 12.0 Hz, 1H); ^{13}C NMR (100 MHz, CD_3CN) δ 170.9, 156.3, 152.2, 150.9, 137.8, 134.5, 134.4, 134.3, 132.0, 131.5, 131.4, 131.2, 131.0, 130.8, 130.4, 130.0, 129.2, 128.5, 128.0, 126.5, 125.9, 124.6, 115.7, 115.4, 114.9, 113.4, 79.8, 54.4, 47.6, 43.1, 38.8; IR (thin film) 3361, 3278, 3091, 2929, 2262, 1772, 1737, 1684, 1606, 1530, 1493, 1388, 1351 cm^{-1}; HRMS (ESI) calculated for $C_{35}H_{23}N_7O_6H$ $[M+H]^+$ 638.1783, found 638.1778.

7a-Hydroxy-3-imino-4,6-bis(3-nitrophenyl)-1-oxo-2,7-diphenylhexahydro-5*H*-isoindole-3a,5,5(4*H*)-tricarbonitrile (*rac*-3j, major)

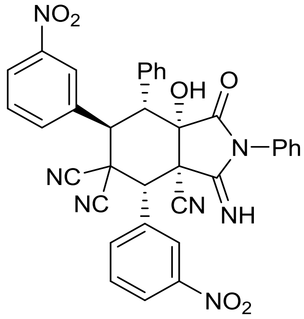

The reaction was stirred for 12 h. Purification by column chromatography (30% EtOAc/Petrol) gave 7a-hydroxy-3-imino-4,6-bis(3-nitrophenyl)-1-oxo-2,7-diphenyl-hexahydro-5*H*-isoindole-3a,5,5(4*H*)-tricarbonitrile as a yellow solid (57.9 mg, 0.091 mmol, 91%): m.p.: 200-207 ^{o}C. ^{1}H NMR (400 MHz, CD_3CN) δ 9.48 (s, 1H), 9.14 (s, 1H), 8.55–7.18 (m, 17H), 5.45 (s, 1H), 4.77 (s, 1H), 4.67 (s, 1H), 4.18 (s, 1H); ^{13}C NMR (100 MHz, CD_3CN) δ 170.8, 153.1, 148.9, 139.5, 136.7, 134.7, 134.0, 132.5, 131.3, 131.1, 130.8, 130.6, 130.4, 130.0, 128.9, 128.7, 128.1, 128.0, 125.8, 124.6, 113.7, 79.8, 56.4, 48.7, 48.5, 48.3, 45.8; IR (thin film) 3426, 2254, 2127, 1656, 1531, 1352 cm^{-1}; HRMS (ESI) calculated for $C_{35}H_{23}N_7O_6H$ $[M+H]^+$ 638.1783, found 638.1787.

7a-Hydroxy-3-imino-4,6-bis(4-nitrophenyl)-1-oxo-2,7-diphenylhexahydro-5*H*-isoindole-3a,5,5(4*H*)-tricarbonitrile (*rac*-3k, major)

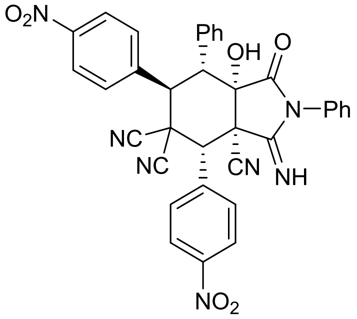

The reaction was stirred for 12 h. Purification by column chromatography (30% EtOAc/Petrol) gave 7a-hydroxy-3-imino-4,6-bis(4-nitrophenyl)-1-oxo-2,7-diphenyl-hexahydro-5*H*-isoindole-3a,5,5(4*H*)-tricarbonitrile as a yellow solid (26.8 mg, 0.042 mmol, 42%): m.p.: 205-212 ^{o}C. ^{1}H NMR (400 MHz, CD_3CN) δ 8.64 (s, 1H), 8.47–8.45 (d, 2H), 8.19–8.15 (br, 4H), 7.68–7.06 (m, 12H), 5.16 (s, 1H), 5.11 (s, 1H), 4.94 (d, J = 12.0 Hz, 1H), 4.10 (d, J = 12.0 Hz, 1H); ^{13}C NMR (100 MHz, CD_3CN) δ

168.2, 154.3, 148,6, 148.4, 147.4, 141.6, 140.1, 132.8, 132.7, 132.6, 131.1, 129.7, 128.7, 127.8, 127.1, 124.2, 123.5, 112.6, 112.2, 112.1, 76.8, 57.5, 55.8, 47.9, 47.4, 45.4; IR (thin film) 3429, 2927, 2253, 2126, 1718, 1656, 1525, 1350 cm^{-1}; HRMS (ESI) calculated for $C_{35}H_{23}N_7O_6H$ $[M+H]^+$ 638.1783, found 638.1780.

7a-Hydroxy-3-imino-4,6-di(naphthalen-2-yl)-1-oxo-2,7-diphenylhexahydro-5*H*-isoindole-3a,5,5(4*H*)-tricarbonitrile (*rac*-3l, major)

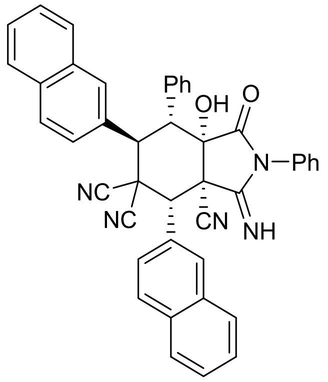

The reaction was stirred for 12 h. Purification by column chromatography (25% EtOAc/Petrol) gave 7a-hydroxy-3-imino-4,6-di(naphthalen-2-yl)-1-oxo-2,7-diphenyl-hexahydro-5*H*-isoindole-3a,5,5(4*H*)-tricarbonitrile as a white solid (58.2 mg, 0.090 mmol, 90%): m.p.: 213-220 °C. ^{1}H NMR (400 MHz, CD_3CN) δ 8.78 (s, 1H), 8.04–7.95 (m, 4H), 7.80–7.73 (m, 4H), 7.65–7.60 (m, 7H), 7.48–7.43 (m, 5H), 7.15–7.07 (m, 4H), 5.35 (s, 1H), 4.80 (d, J = 12.0 Hz, 1H), 4.67 (s, 1H), 4.23 (d, J = 12.0 Hz, 1H); ^{13}C NMR (100 MHz, CD_3CN) δ 171.3, 153.4, 134.3, 133.6, 133.4, 133.2, 133.1, 132.8, 132.5, 131.5, 131.1, 130.9, 130.8, 130.0, 129.6, 128.9, 128.6, 128.5, 128.4, 128.3, 128.2, 128.2, 128.1, 128.1, 128.0, 127.6, 127.5, 127.4, 116.6, 114.5, 114.4, 79.9, 56.9, 49.5, 46.6; IR (thin film) 3444, 3368, 3271, 3063, 2930, 2261, 1769, 1685, 1598, 1493, 1456, 1382 cm^{-1}; HRMS (ESI) calculated for $C_{43}H_{29}N_5O_2H$ $[M+H]^+$ 648.2394, found 648.2398.

7a-Hydroxy-3-imino-1-oxo-2,7-diphenyl-4,6-di(thiophen-2-yl)hexahydro-5*H*-isoindole-3a,5,5(4*H*)-tricarbonitrile (*rac*-3m, major)

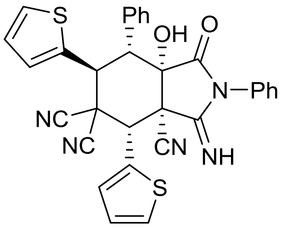

The reaction was stirred for 12 h. Purification by column chromatography (25% EtOAc/Petrol) gave 7a-hydroxy-3-imino-1-oxo-2,7-diphenyl-4,6-di(thiophen-2-yl)hexahydro-5*H*-isoindole-3a,5,5(4*H*)-tricarbonitrile as a yellow solid (28.5 mg, 0.051 mmol, 51%): m.p.: 183-187 ^{o}C. ^{1}H NMR (400 MHz, CD_3CN) δ 8.85 (s, 1H),7.66–7.59 (m, 4H), 7.40–7.13 (m, 11H), 6.85–6.82 (m, 1H), 5.51 (s, 1H), 5.13 (s, 1H), 4.84 (d, J = 12.0 Hz, 1H), 3.91 (d, J = 12.0 Hz, 1H); ^{13}C NMR (100 MHz, CD_3CN) δ 170.9, 154.4, 136.5, 134.8, 133.2, 132.1, 130.7, 130.6, 130.5, 130.2, 129.8, 129.1, 128.8, 128.5, 128.2, 128.1, 127.9, 127.8, 127.3, 127.0, 126.8, 121.3, 115.1, 113.7, 113.3, 77.9, 54.2, 49.8, 46.4, 41.2, 40.6; IR (thin film) 3631, 3279, 3105, 2930, 2261, 1770, 1682, 1597, 1541, 1494, 1456, 1387 cm^{-1}; HRMS (ESI) calculated for $C_{31}H_{21}N_5O_2S_2H$ [M+H]$^+$ 560.1209, found 560.1215.

4,6-Bis(3,4-dichlorophenyl)-7a-hydroxy-3-imino-1-oxo-2,7-diphenylhexahydro-5*H*-isoindole-3a,5,5(4*H*)-tricarbonitrile (*rac*-3n, major)

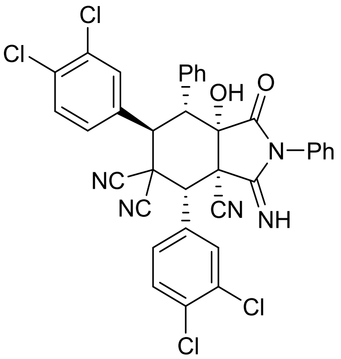

The reaction was stirred for 12 h. Purification by column chromatography (20% EtOAc/Petrol) gave 4,6-bis(3,4-dichlorophenyl)-7a-hydroxy-3-imino-1-oxo-2,7-diphenylhexahydro-5*H*-isoindole-3a,5,5(4*H*)-tricarbonitrile as a white solid (57.5 mg, 0.084 mmol, 84%): m.p.: 185-191 ^{o}C. ^{1}H NMR (400 MHz, CD_3CN) δ 8.83 (s, 1H), 7.78–7.76 (d, 1H), 7.64–7.58 (m, 5H), 7.40–7.21 (m, 10H), 5.17 (s, 1H), 4.43 (s, 1H),

4.38 (d, J = 12.0 Hz, 1H), 4.00 (d, J = 12.0 Hz, 1H); ^{13}C NMR (100 MHz, CD_3CN) δ 171.4, 155.0, 135.5, 135.4, 134.9, 134.7, 134.1, 133.6, 133.5, 133.4, 133.2, 132.0, 131.6, 131.3, 131.2, 131.1, 131.0, 130.9, 130.8, 130.8, 130.0, 128.9, 128.8, 128.1, 127.9, 113.8, 113.7, 113.5, 78.3, 53.6, 47.7, 45.9, 45.8, 44.0; IR (thin film) 3274, 3093, 3068, 2928, 2262, 1771, 1686, 1595, 1493, 1476, 1458, 1384 cm^{-1}; HRMS (ESI) calculated for $C_{35}H_{21}Cl_4N_5O_2H$ [M+H]$^+$ 684.0522, found 684.0530.

4,6-Bis(3,5-dimethoxyphenyl)-7a-hydroxy-3-imino-1-oxo-2,7-diphenylhexahydro-5*H*-isoindole-3a,5,5(4*H*)-tricarbonitrile (*rac*-3o, major)

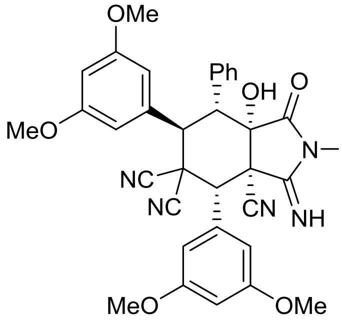

The reaction was stirred for 12 h. Purification by column chromatography (25% EtOAc/Petrol) gave 4,6-bis(3,5-dimethoxyphenyl)-7a-hydroxy-3-imino-1-oxo-2,7-diphenylhexahydro-5*H*-isoindole-3a,5,5(4*H*)-tricarbonitrile as a white solid (36.7 mg, 0.055 mmol, 55%): m.p.: 201-208 ^{o}C. ^{1}H NMR (400 MHz, CD_3CN) δ 8.79 (s, 1H), 7.63–6.70 (m, 16H), 5.13 (s, 1H), 5.07 (s, 1H), 4.45 (d, J = 12.0 Hz, 1H), 4.34 (d, J = 12.0 Hz, 1H), 3.94 (s, 3H), 3.89 (s, 3H), 3.68 (s, 3H), 3.67 (s, 3H); ^{13}C NMR (100 MHz, CD_3CN) δ 172.0, 155.7, 153.5, 151.4, 151.0, 149.9, 149.7, 149.2, 149.0, 134.3, 131.8, 131.5, 131.0, 130.7, 130.6, 129.9, 128.4, 128.3, 128.2, 128.1, 127.9, 127.1, 127.0, 125.3, 116.7, 116.3, 112.1, 111.5, 111.3, 78.5, 56.3, 56.2, 56.0, 55.8, 54.2, 47.4, 47.3, 47.2, 46.9; IR (thin film) 3524, 3440, 3268, 3009, 2963, 2940, 2841, 2261, 1768, 1682, 1594, 1520, 1466, 1426, 1390 cm^{-1}; HRMS (ESI) calculated for $C_{39}H_{34}N_5O_6H$ [M+H]$^+$ 668.2504, found 668.2509.

7a-Hydroxy-3-imino-2-(4-methoxyphenyl)-1-oxo-4,6,7-triphenylhexahydro-5*H*-isoindole-3a,5,5(4*H*)-tricarbonitrile (*rac*-3p, major)

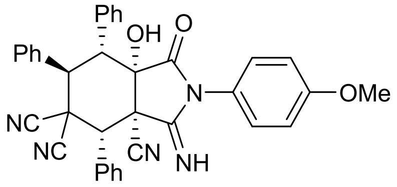

The reaction was stirred for 12 h. Purification by column chromatography (25% EtOAc/Petrol) gave 7a-hydroxy-3-imino-2-(4-methoxyphenyl)-1-oxo-4,6,7-triphenylhexahydro-5*H*-isoindole-3a,5,5(4*H*)-tricarbonitrile as a white solid (32.3 mg, 0.056 mmol, 56%): m.p.: 192-198 °C. ^{1}H NMR (400 MHz, $CDCl_3$) δ 8.52 (s, 1H), 7.58–6.97 (m, 19H), 5.25 (s, 1H), 4.27 (d, *J* = 12.0 Hz, 1H), 3.95 (d, *J* = 12.0 Hz, 1H), 3.88 (s, 3H), 3.72 (s, 1H); ^{13}C NMR (100 MHz, $CDCl_3$) δ 171.9, 161.0, 155.7, 133.5, 132.9, 132.0, 130.4, 129.5, 128.9, 128.7, 128.6, 128.4, 128.1, 122.6, 115.9, 114.7, 114.1, 112.3, 77.2, 55.7, 53.4, 52.3, 49.5, 47.7, 45.9, 42.5; IR (thin film) 3367, 3273, 3067, 3037, 2940, 2843, 2261, 1768, 1679, 1608, 1513, 1458, 1396 cm^{-1}; HRMS (ESI) calculated for $C_{36}H_{27}N_5O_3H$ $[M+H]^+$ 578.2187, found 578.2184.

2-(4-Chlorophenyl)-7a-hydroxy-3-imino-1-oxo-4,6,7-triphenylhexahydro-5*H*-isoindole-3a,5,5(4*H*)-tricarbonitrile (*rac*-3q, major)

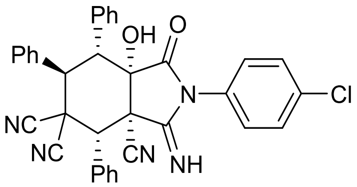

The reaction was stirred for 12 h. Purification by column chromatography (25% EtOAc/Petrol) gave 2-(4-chlorophenyl)-7a-hydroxy-3-imino-1-oxo-4,6,7-triphenylhexahydro-5*H*-isoindole-3a,5,5(4*H*)-tricarbonitrile as a white solid (51.7 mg, 0.089 mmol, 89%): m.p.: 189-194 °C. ^{1}H NMR (400 MHz, CD_3CN) δ 8.79 (s, 1H), 7.59–7.16 (m, 19H), 5.08 (s, 1H), 5.05 (s, 1H), 4.42 (d, *J* = 12.0 Hz, 1H), 4.11 (d, *J* = 12.0 Hz, 1H); ^{13}C NMR (100 MHz, CD_3CN) δ 171.0, 154.6, 134.3, 134.0, 133.4, 132.3, 130.5, 130.1, 129.8, 129.6, 129.4, 129.3, 129.1, 129.0, 128.9, 128.8, 128.7, 128.5, 128.4, 128.2, 127.8, 127.7, 127.5, 127.0, 113.9, 113.7, 113.6, 77.7, 53.2, 49.5, 47.2, 46.6, 44.6; IR (thin film) 3514, 3278, 3094, 3067, 3037, 2261, 1768, 1680, 1595, 1494, 1457, 1393 cm^{-1}; HRMS (ESI) calculated for $C_{35}H_{24}ClN_5O_2H$ $[M+H]^+$

582.1691, found 582.1696.

7a-Hydroxy-3-imino-2-(4-methoxyphenyl)-4,6-di(naphthalen-2-yl)-1-oxo-7-phenylhexahydro-5*H*-isoindole-3a,5,5(4*H*)-tricarbonitrile (*rac*-3r, major)

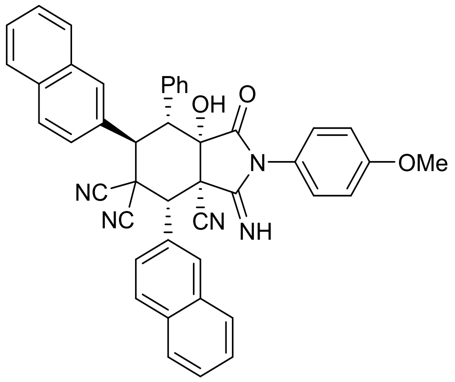

The reaction was stirred for 12 h. Purification by column chromatography (30% EtOAc/Petrol) gave 7a-hydroxy-3-imino-2-(4-methoxyphenyl)-4,6-di(naphthalen-2-yl)-1-oxo-7-phenylhexahydro-5*H*-isoindole-3a,5,5(4*H*)-tricarbonitrile as a white solid (59.5 mg, 0.088 mmol, 88%): m.p.: 214-221 ^{o}C. ^{1}H NMR (400 MHz, CD_3CN) δ 9.02 (s, 1H), 8.79 (s, 1H), 8.51 (s, 1H), 8.04–7.95 (m, 4H), 7.81–7.59 (m, 6H), 7.48–7.35 (m, 6H), 7.17–7.07 (m, 5H), 5.35 (s, 1H), 4.80 (d, *J* = 12.0 Hz, 1H), 4.67 (s, 1H), 4.23 (d, *J* = 12.0 Hz, 1H), 3.86 (s, 3H); ^{13}C NMR (100 MHz, CD_3CN) δ 171.6, 161.5, 161.4, 153.9, 134.3, 133.6, 133.4, 133.2, 133.1, 132.5, 130.9, 130.5, 130.1, 129.7, 129.6, 129.4, 128.9, 128.6, 128.5, 128.4, 128.3, 128.2, 128.1, 128.1, 127.6, 127.5, 127.3, 123.6, 116.3, 114.5, 114.4, 79.8, 56.7, 56.1, 49.5, 49.2, 46.6; IR (thin film) 3330, 3268, 3062, 2962, 2938, 2842, 2261, 1768, 1684, 1606, 1512, 1457, 1443, 1389 cm^{-1}; HRMS (ESI) calculated for $C_{44}H_{31}N_5O_3H$ $[M+H]^+$ 678.2500, found 678.2507.

7a-Hydroxy-3-imino-1-oxo-2,4,6-triphenyl-7-(*p*-tolyl)hexahydro-5*H*-isoindole-3a, 5,5(4*H*)-tricarbonitrile (*rac*-3s, major)

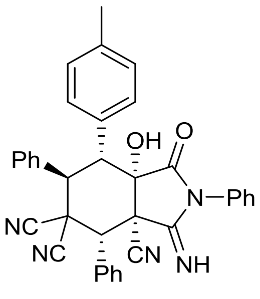

The reaction was stirred for 12 h. Purification by column chromatography (25%

EtOAc/Petrol) gave 7a-hydroxy-3-imino-1-oxo-2,4,6-triphenyl-7-(*p*-tolyl)hexahydro-5*H*-isoindole-3a,5,5(4*H*)-tricarbonitrile as a white solid (34.2 mg, 0.061 mmol, 61%): m.p.: 192-198 °C. ^{1}H NMR (400 MHz, CD_3CN) δ 8.75 (s, 1H), 7.64–7.58 (m, 6H), 7.52–7.35 (m, 6H), 7.19–6.99 (m, 7H), 5.08 (s, 1H), 4.98 (s, 1H), 4.41 (d, *J* = 12.0 Hz, 1H), 4.05 (d, *J* = 12.0 Hz, 1H), 2.21 (s, 3H); ^{13}C NMR (100 MHz, CD_3CN) δ 171.9, 155.7, 138.2, 135.1, 135.0, 133.0, 131.8, 131.3, 131.1, 130.9, 130.8, 130.7, 130.4, 129.9, 129.8, 129.7, 129.4, 128.9, 128.0, 116.2, 114.2, 78.4, 53.8, 47.8, 47.2, 46.6, 45.0, 20.8; IR (thin film) 3438, 3284, 3066, 3037, 2927, 2262, 1768, 1679, 1597, 1494, 1457, 1390 cm^{-1}; HRMS (ESI) calculated for $C_{36}H_{27}N_5O_2H$ [M+H]$^+$ 562.2238, found 562.2242.

7-(4-Fluorophenyl)-7a-hydroxy-3-imino-1-oxo-2,4,6-triphenylhexahydro-5*H*-isoindole-3a,5,5(4*H*)-tricarbonitrile (*rac*-3t, major)

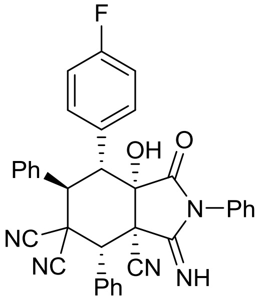

The reaction was stirred for 12 h. Purification by column chromatography (25% EtOAc/Petrol) gave 7-(4-fluorophenyl)-7a-hydroxy-3-imino-1-oxo-2,4,6-triphenyl-hexahydro-5*H*-isoindole-3a,5,5(4*H*)-tricarbonitrile as a white solid (34.4 mg, 0.061 mmol, 61%): m.p.: 184-191 °C. ^{1}H NMR (400 MHz, CD_3CN) δ 8.68 (s, 1H), 7.63–7.45 (m, 8H), 7.35–7.21 (m, 9H), 6.92 (br, 2H), 5.21 (s, 1H), 5.03 (s, 1H), 4.43 (d, *J* = 12.0 Hz, 1H), 4.16 (d, *J* = 12.0 Hz, 1H); ^{13}C NMR (100 MHz, CD_3CN) δ 171.7, 164.0, 155.8, 134.8, 134.7, 134.6, 133.5, 132.8, 131.8, 131.3, 131.1, 130.8, 130.5, 130.3, 129.9, 129.8, 129.7, 129.4, 128.9, 128.0, 116.1, 114.5, 114.3, 78.2, 53.7, 47.3, 47.1, 46.7, 45.4; IR (thin film) 3434, 3281, 3068, 2966, 2262, 1767, 1679, 1605, 1511, 1494, 1458, 1393 cm^{-1}; HRMS (ESI) calculated for $C_{35}H_{24}FN_5O_2H$ [M+H]$^+$ 566.1987, found 566.1982.

7a-Hydroxy-1,3-dioxo-2,4,6,7-tetraphenylhexahydro-5*H*-isoindole-3a,5,5(4*H*)-tricarbonitrile (*rac*-4a)

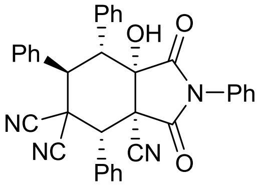

The reaction was stirred for 12 h. Purification by column chromatography (20% EtOAc/Petrol) gave 7a-hydroxy-1,3-dioxo-2,4,6,7-tetraphenylhexahydro-5*H*-isoindole-3a,5,5(4*H*)-tricarbonitrile as a white solid (87.7 mg, 0.160 mmol, 80%): m.p.: 193-197 ^{o}C. ^{1}H NMR (400 MHz, CD_3CN) δ 8.05 (br, 2H), 7.59–7.53 (m, 6H), 7.38–7.37 (m, 6H), 7.21-7.19 (m, 6H), 5.40 (s, 1H), 4.75 (s, 1H), 4.50 (d, *J* = 12.0 Hz, 1H), 4.26 (d, *J* = 12.0 Hz, 1H); ^{13}C NMR (100 MHz, CD_3CN) δ 172.7, 167.0, 135.2, 133.8, 133.2, 131.5, 131.1, 130.5, 130.3, 130.0, 129.5, 129.1, 128.7, 128.5, 127.1, 114.4, 114.1, 78.2, 54.7, 47.2, 47.1, 47.0, 46.6; IR (thin film) 3367, 3068, 3034, 2926, 2851, 2372, 2262, 1739, 1597, 1494, 1458, 1377 cm^{-1}; HRMS (ESI) calculated for $C_{35}H_{24}N_4O_3NH_4$ $[M+NH_4]^+$ 566.2187, found 566.2192.

1. Paradisi, F.; Moynihan, E.; Maguire, A. R. and Engel, P. C. *Org. Biomol. Chem.* **2004**, *2*, 2684-2691. http://dx.doi.org/10.1039/b406364c
2. Liu, S.; Shang, R.; Shi, L.; Wan, D. C. C. and Lin, H. *Eur. J. Med. Chem.* **2014**, *81*, 237-244. http://dx.doi.org/10.1016/j.ejmech.2014.05.020
3. Goudedranche, S.; Pierrot, D.; Constantieux, T.; Bonne, D. and Rodriguez, J. *Chem. Commun.* **2014**, *50*, 15605-15608. http://dx.doi.org/10.1039/C4CC07731H

NMR spectra

^{1}H NMR spectrum of compound 3a ($CDCl_3$, 400 MHz)

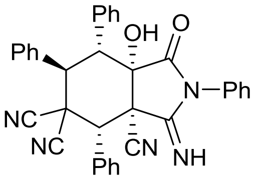

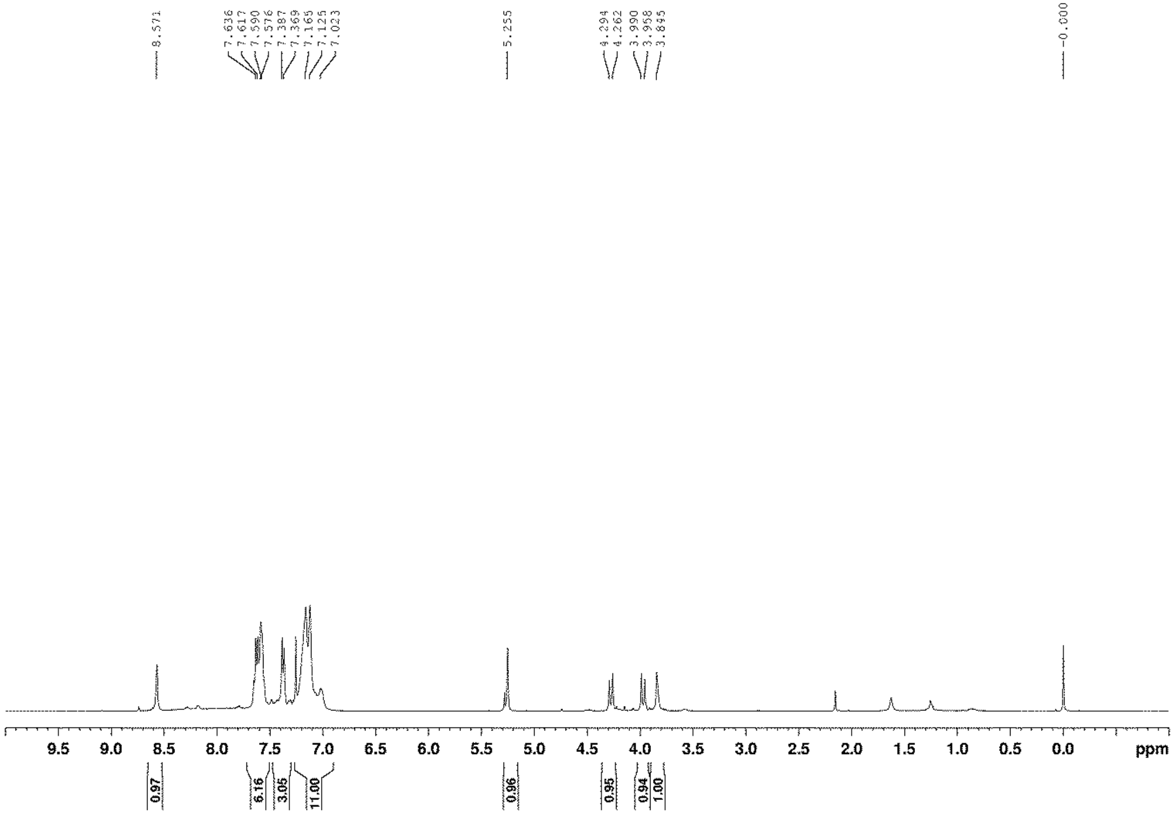

^{13}C NMR spectrum of compound 3a ($CDCl_3$, 100 MHz)

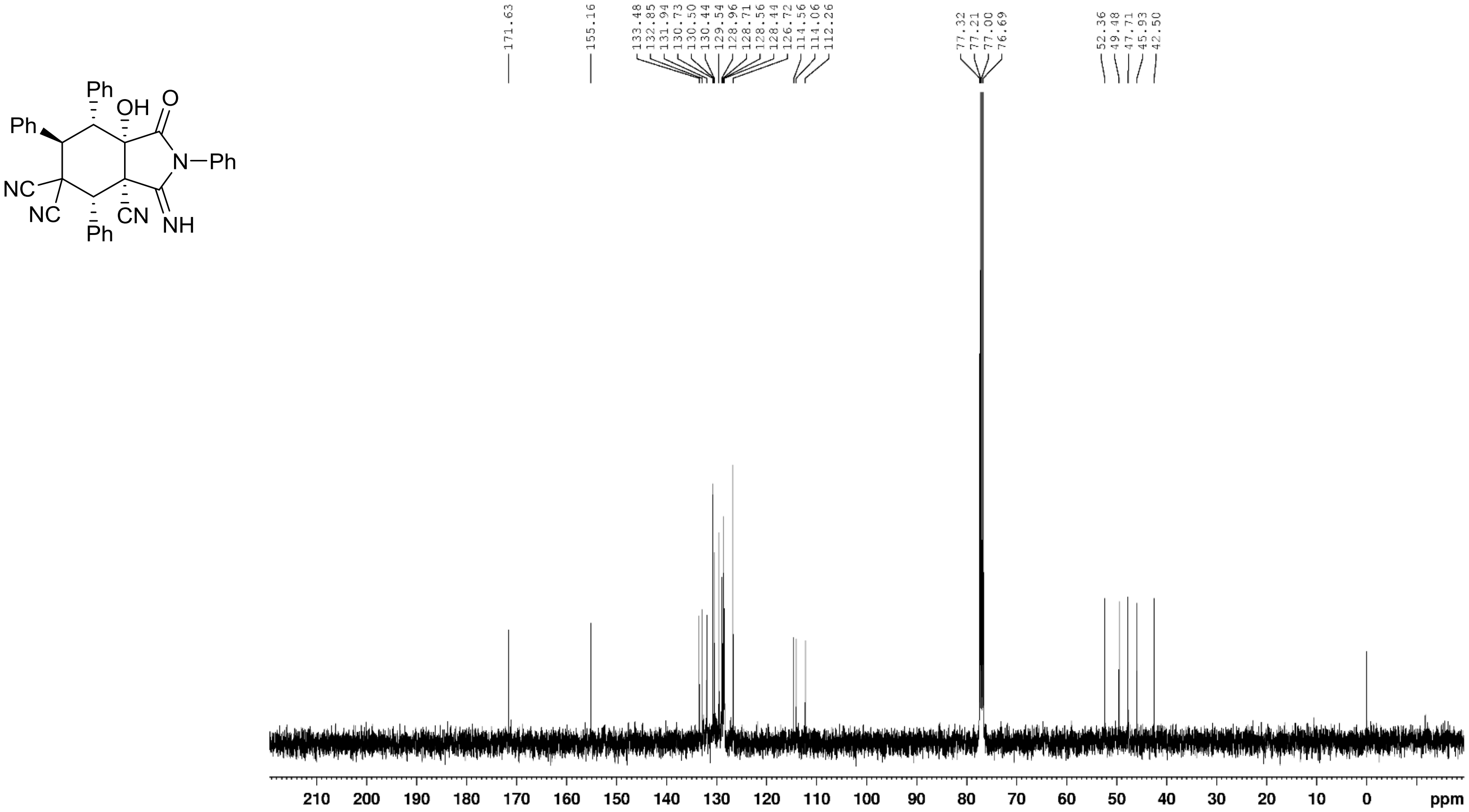

^{1}H NMR spectrum of compound 3b (CD_3CN, 400 MHz)

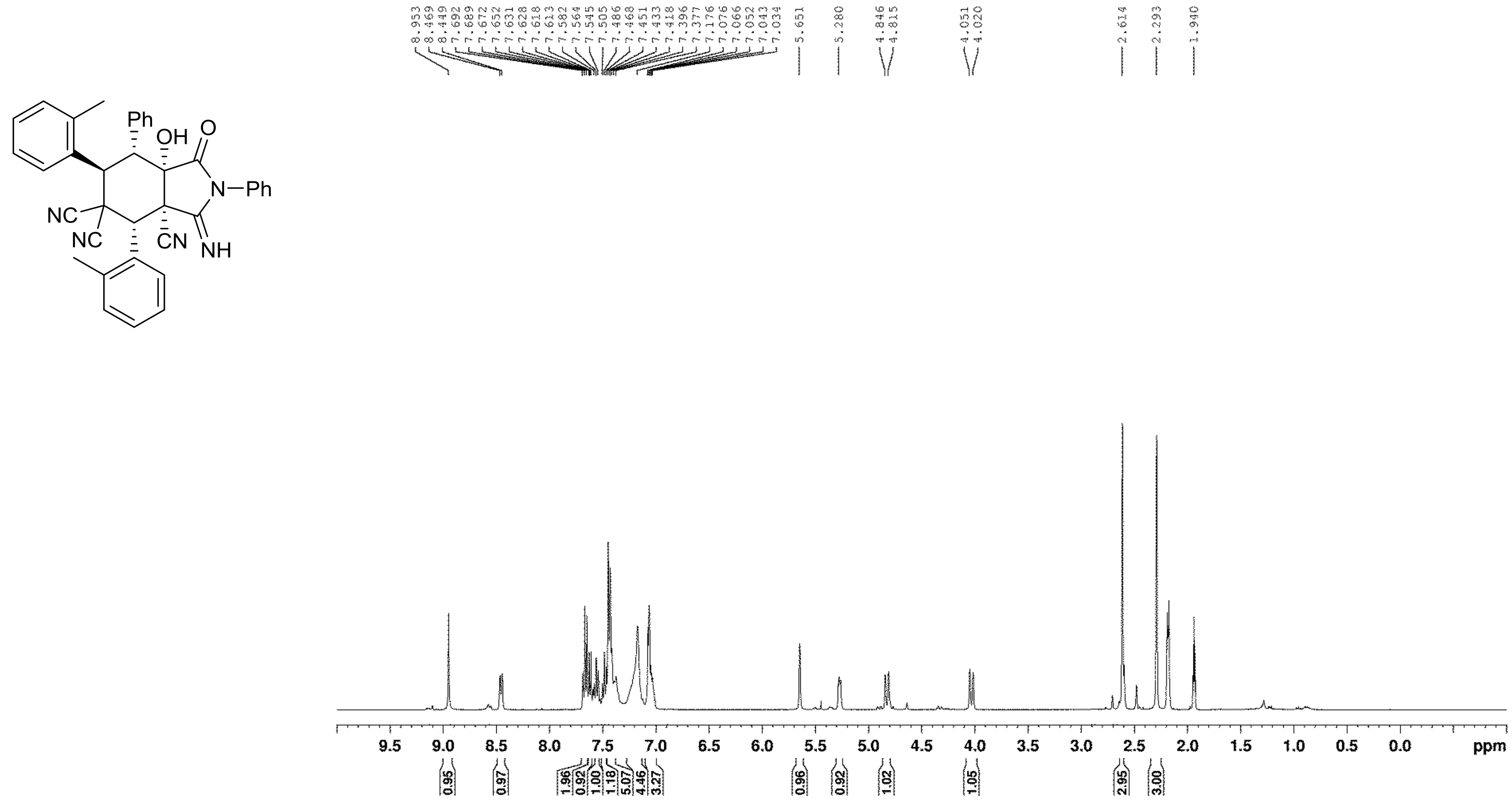

^{13}C NMR spectrum of compound 3b (CD_3CN, 100 MHz)

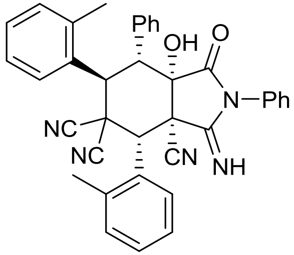

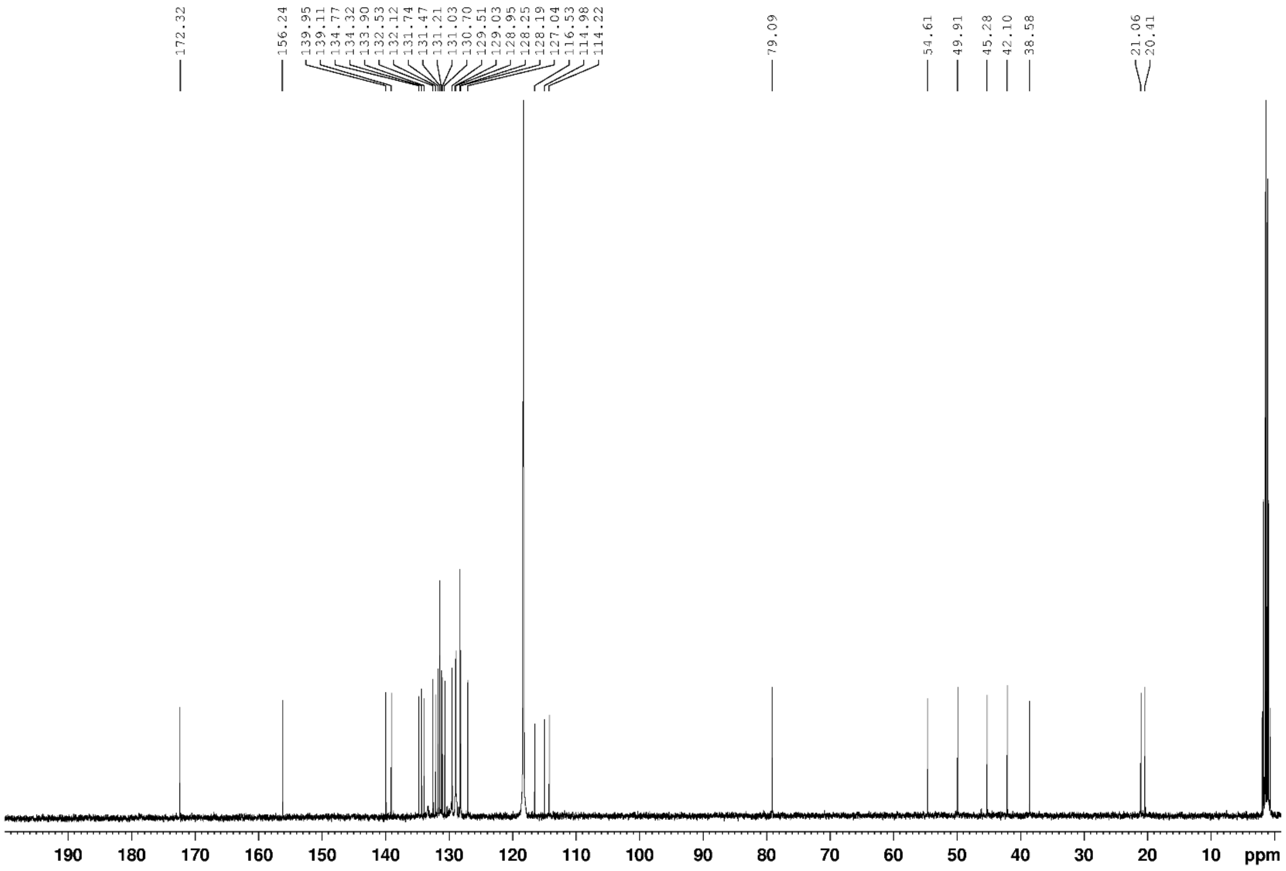

^{1}H NMR spectrum of compound 3c ($CDCl_3$, 400 MHz)

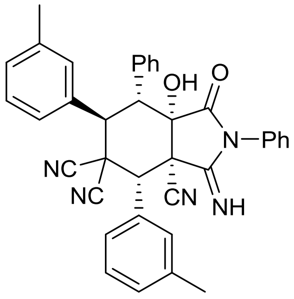

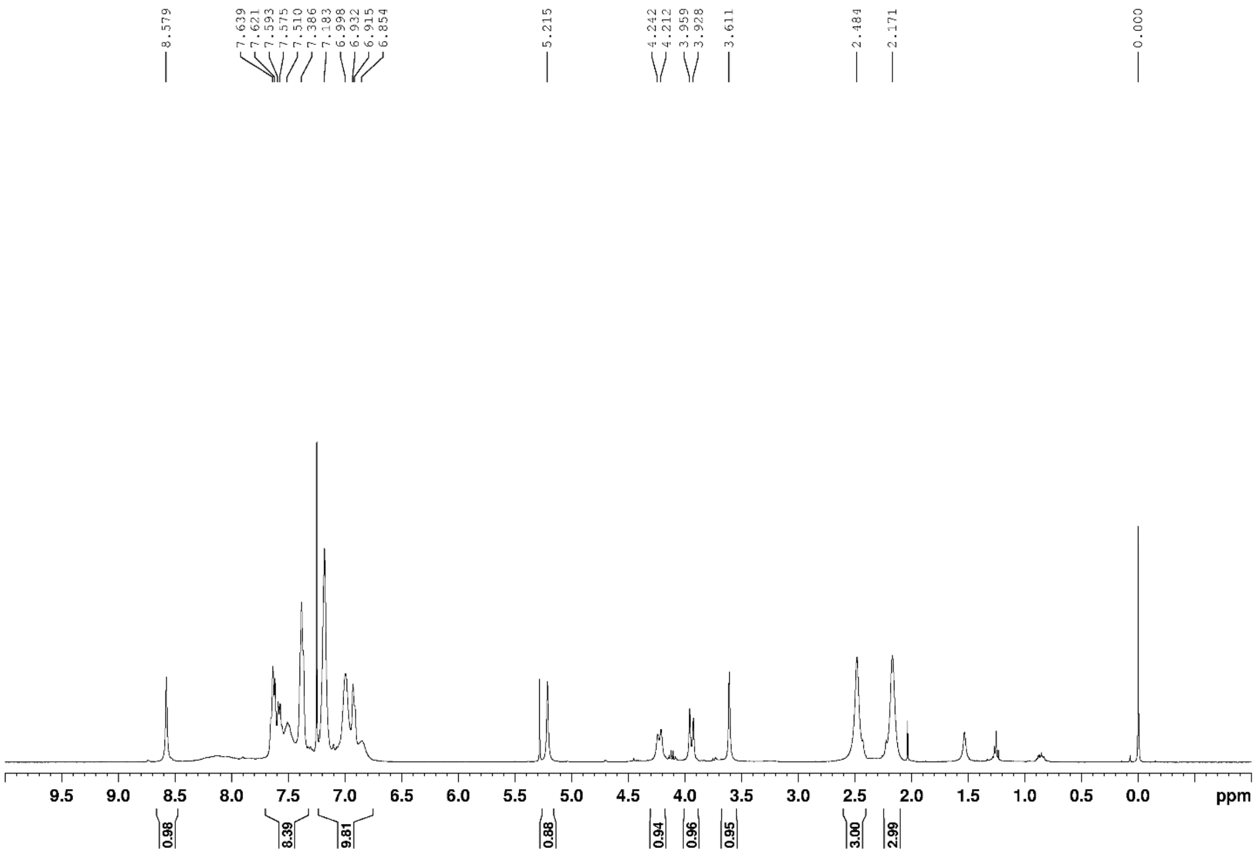

^{13}C NMR spectrum of compound 3c ($CDCl_3$, 100 MHz)

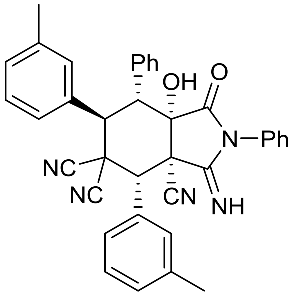

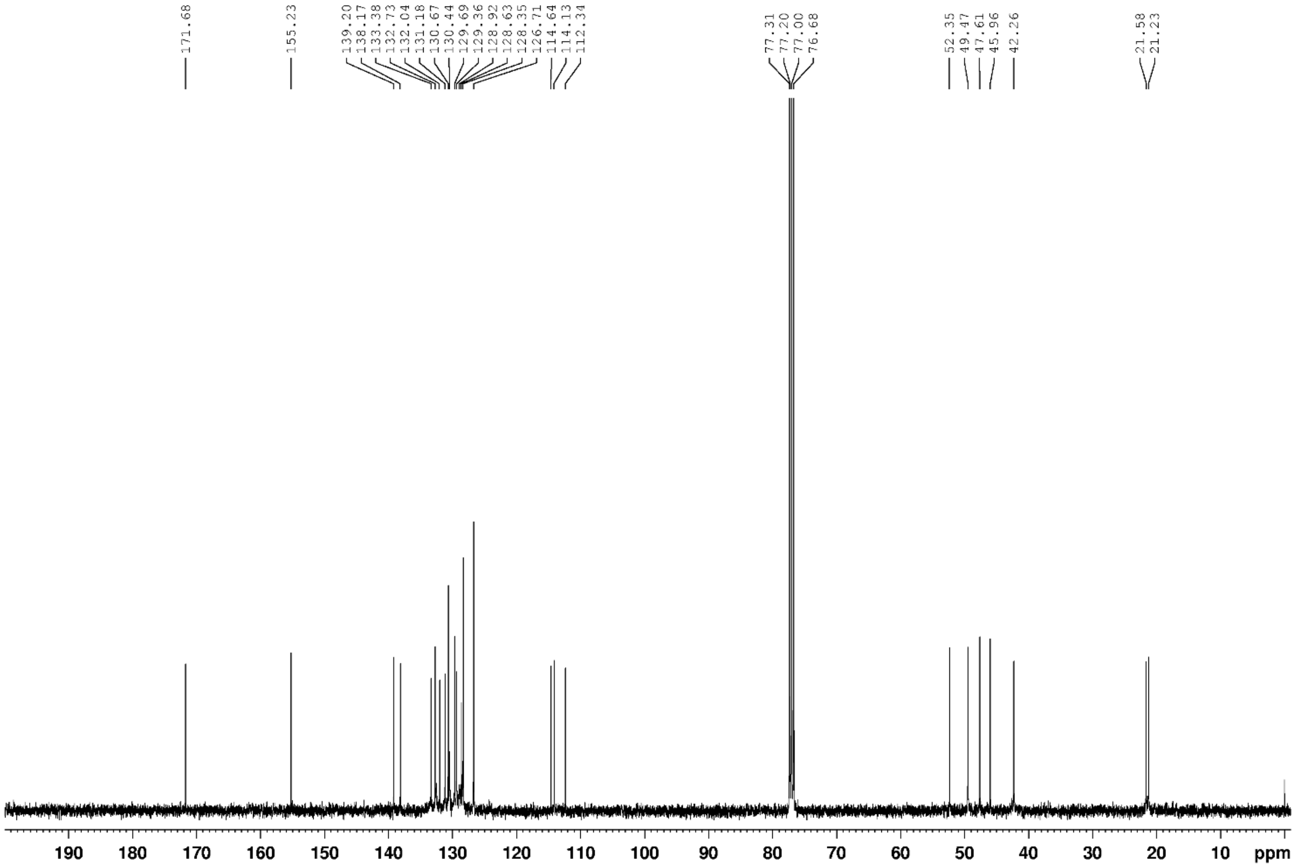

^{1}H NMR spectrum of compound 3d (d_6-DMSO, 400 MHz)

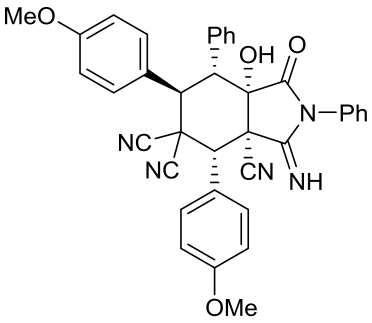

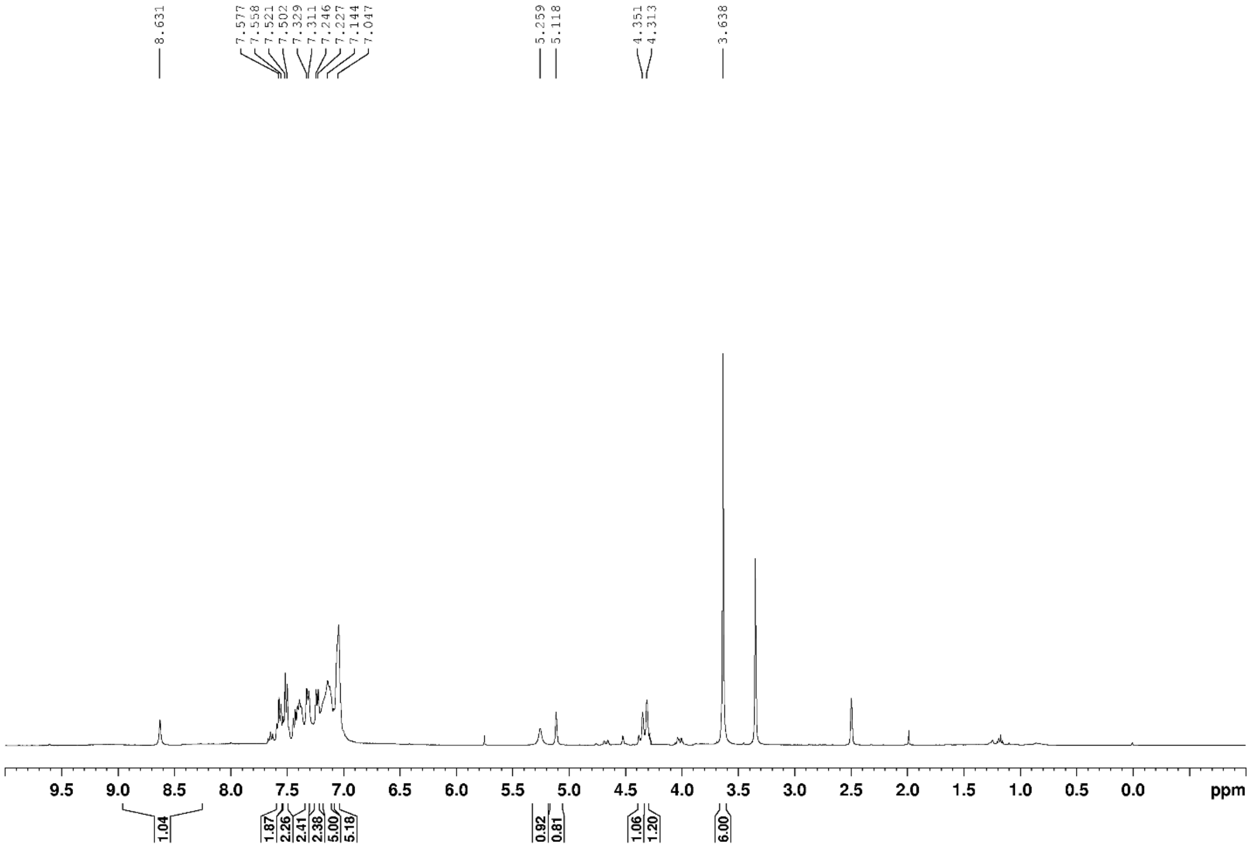

^{13}C NMR spectrum of compound 3d (d_6-DMSO, 100 MHz)

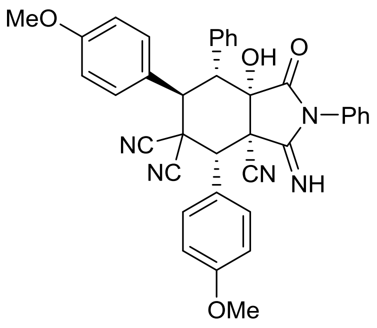

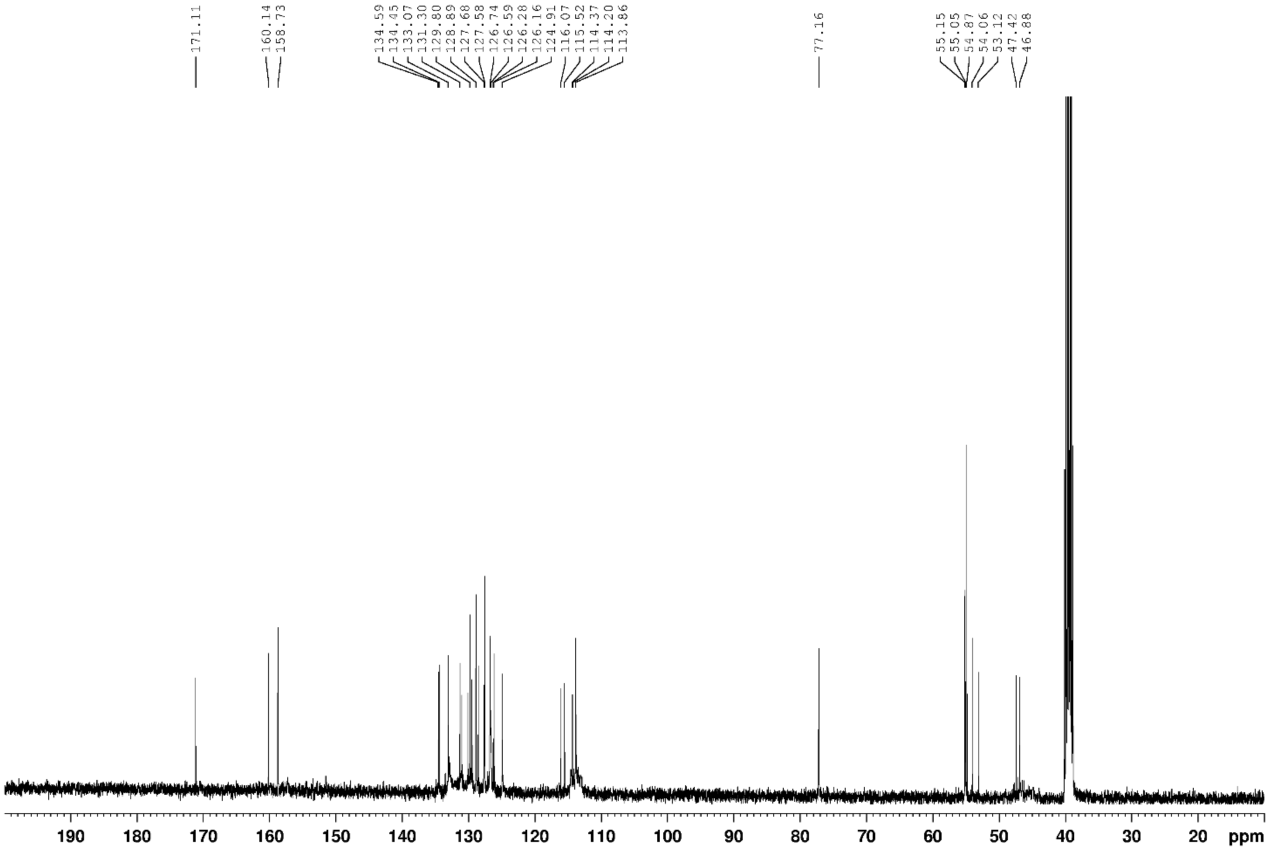

^{1}H NMR spectrum of compound 3e (CD_3CN, 400 MHz)

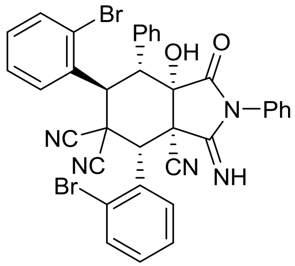

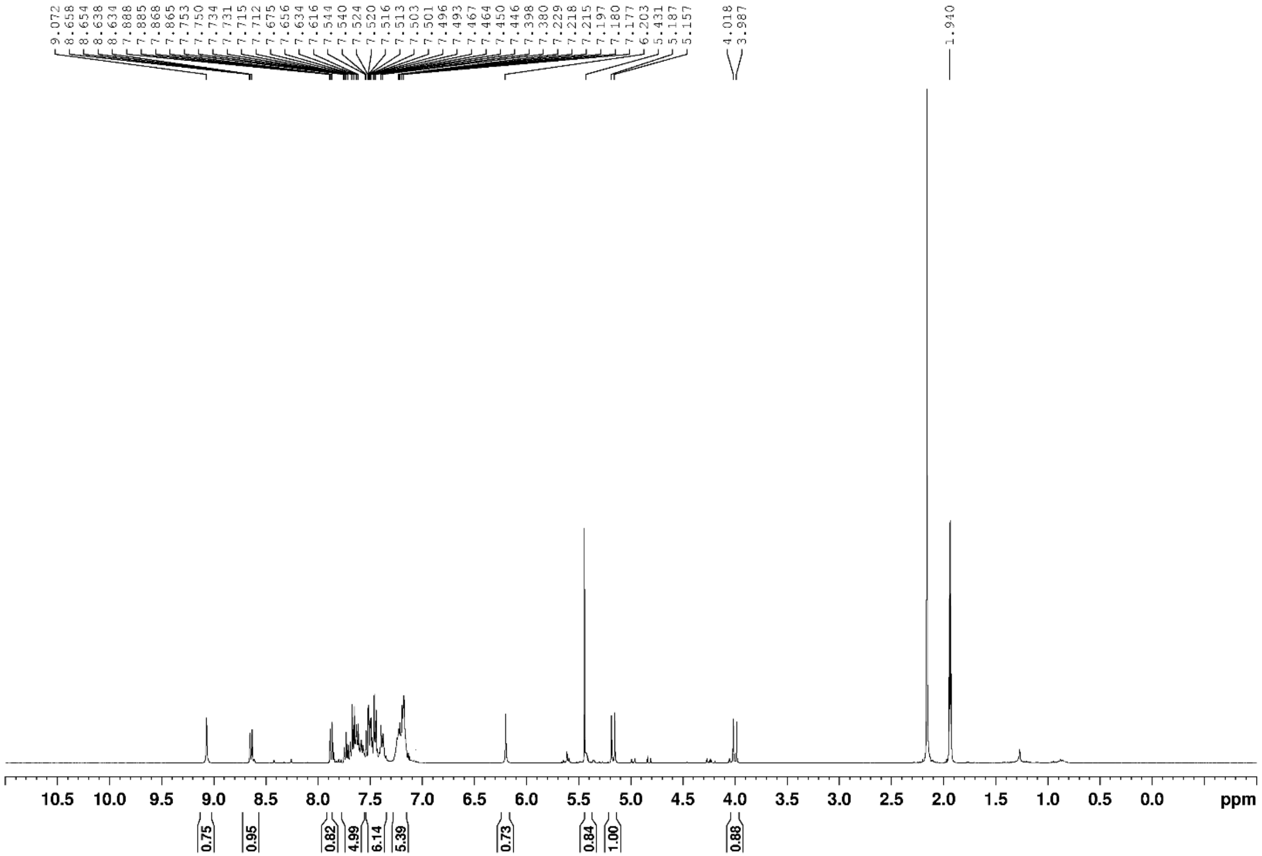

^{13}C NMR spectrum of compound 3e (CD_3CN, 100 MHz)

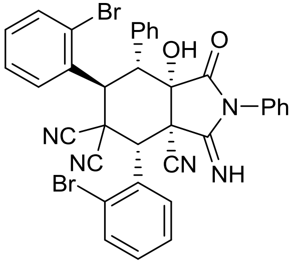

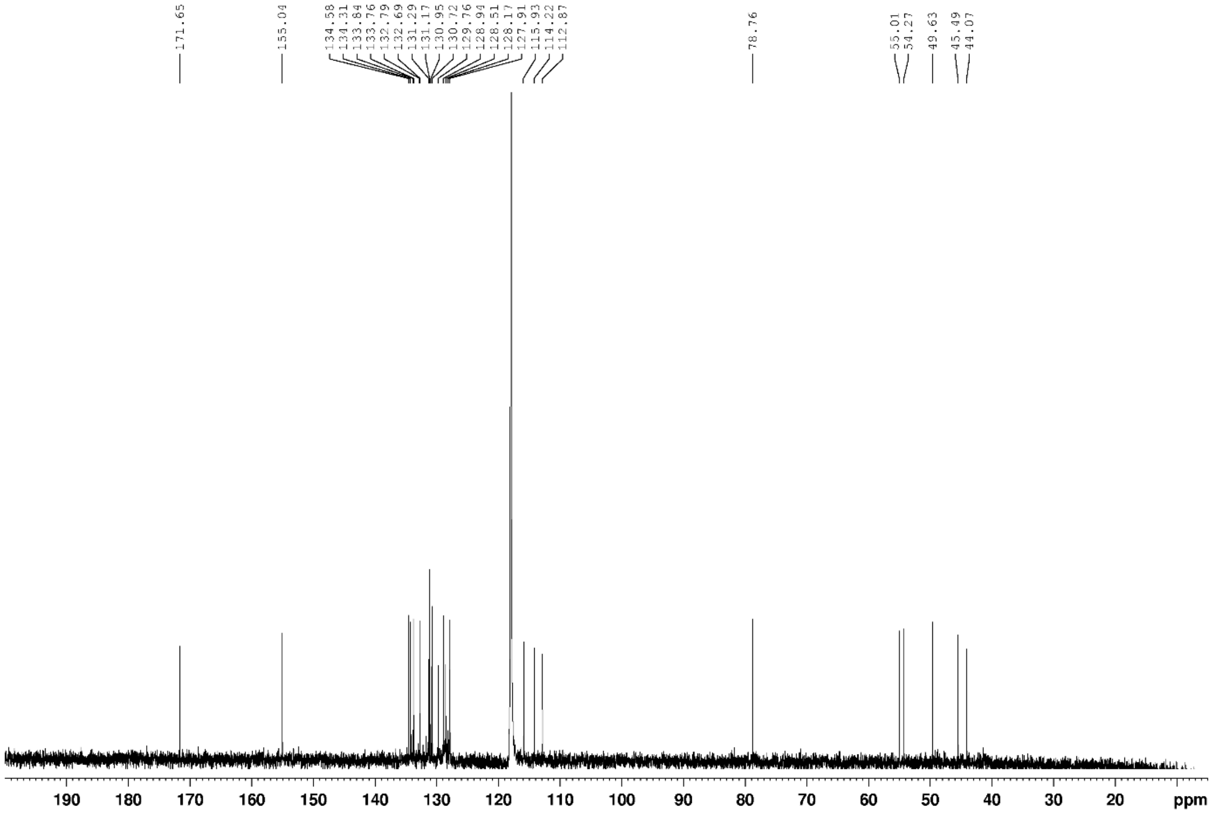

^{1}H NMR spectrum of compound 3f (CD_3CN, 400 MHz)

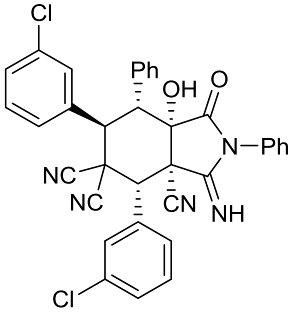

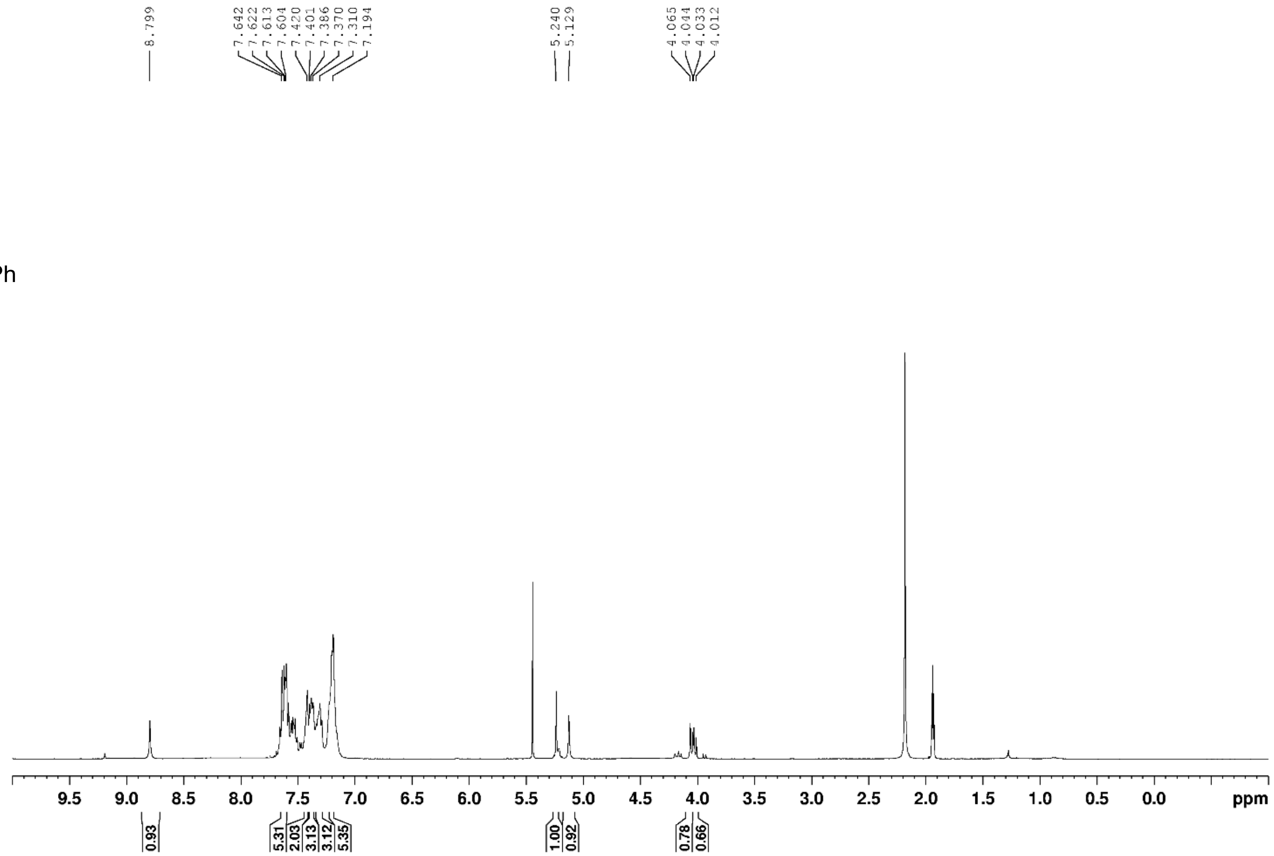

^{13}C NMR spectrum of compound 3f (CD_3CN, 100 MHz)

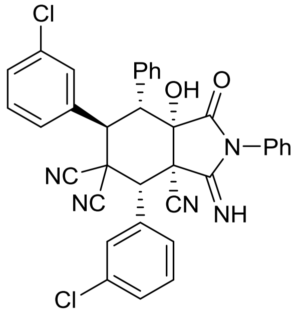

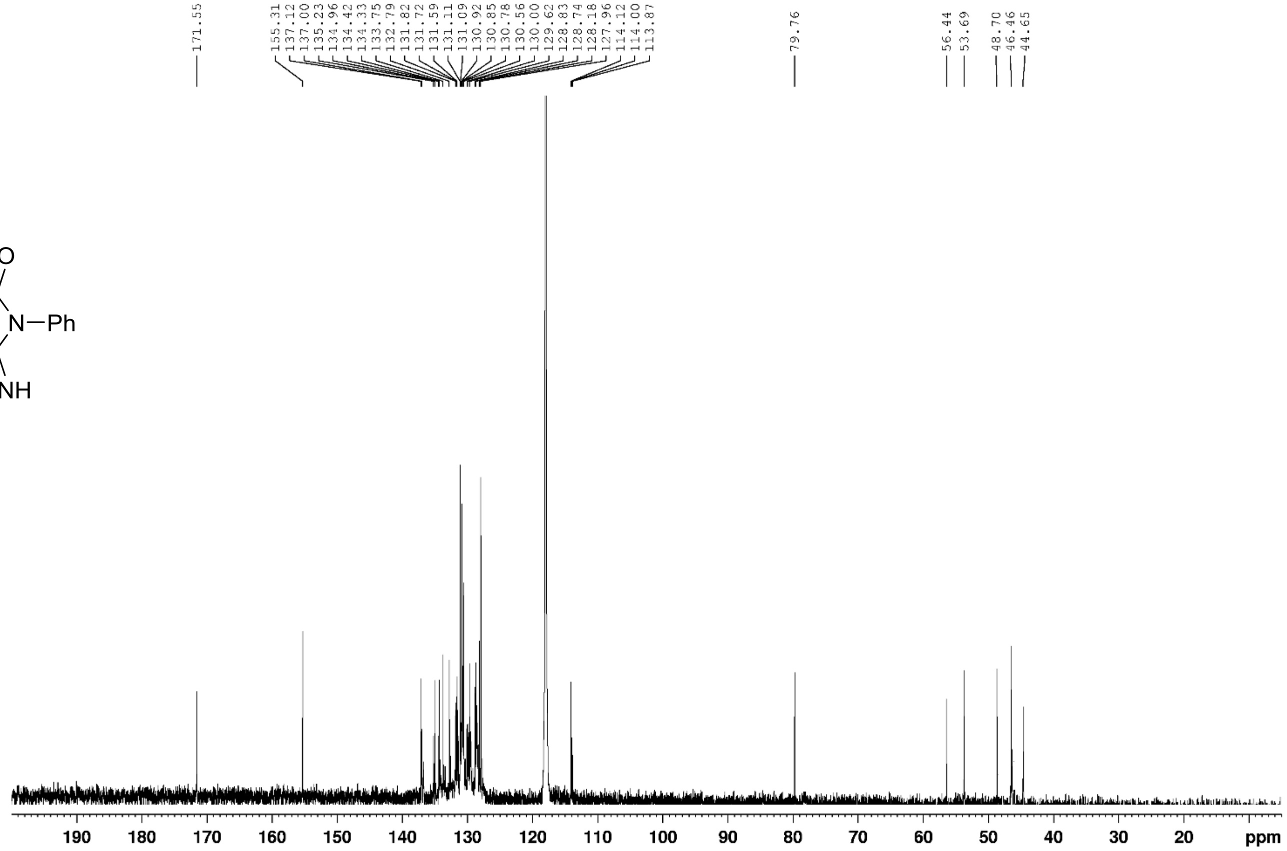

^{1}H NMR spectrum of compound 3g (CD_3CN, 400 MHz)

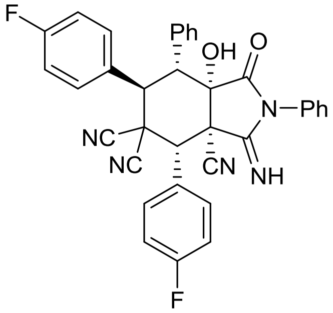

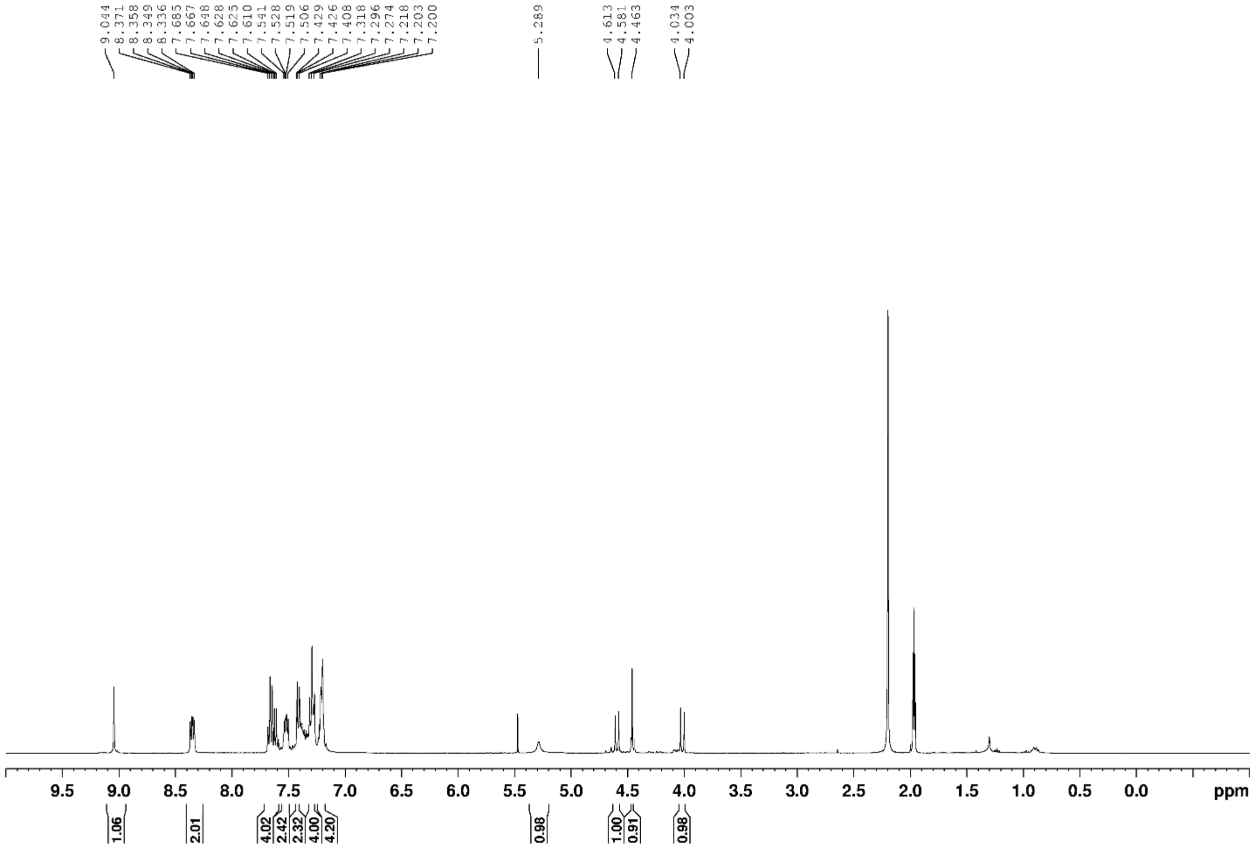

^{13}C NMR spectrum of compound 3g (CD_3CN, 100 MHz)

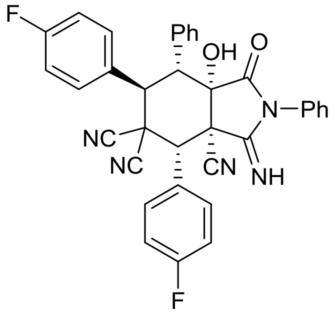

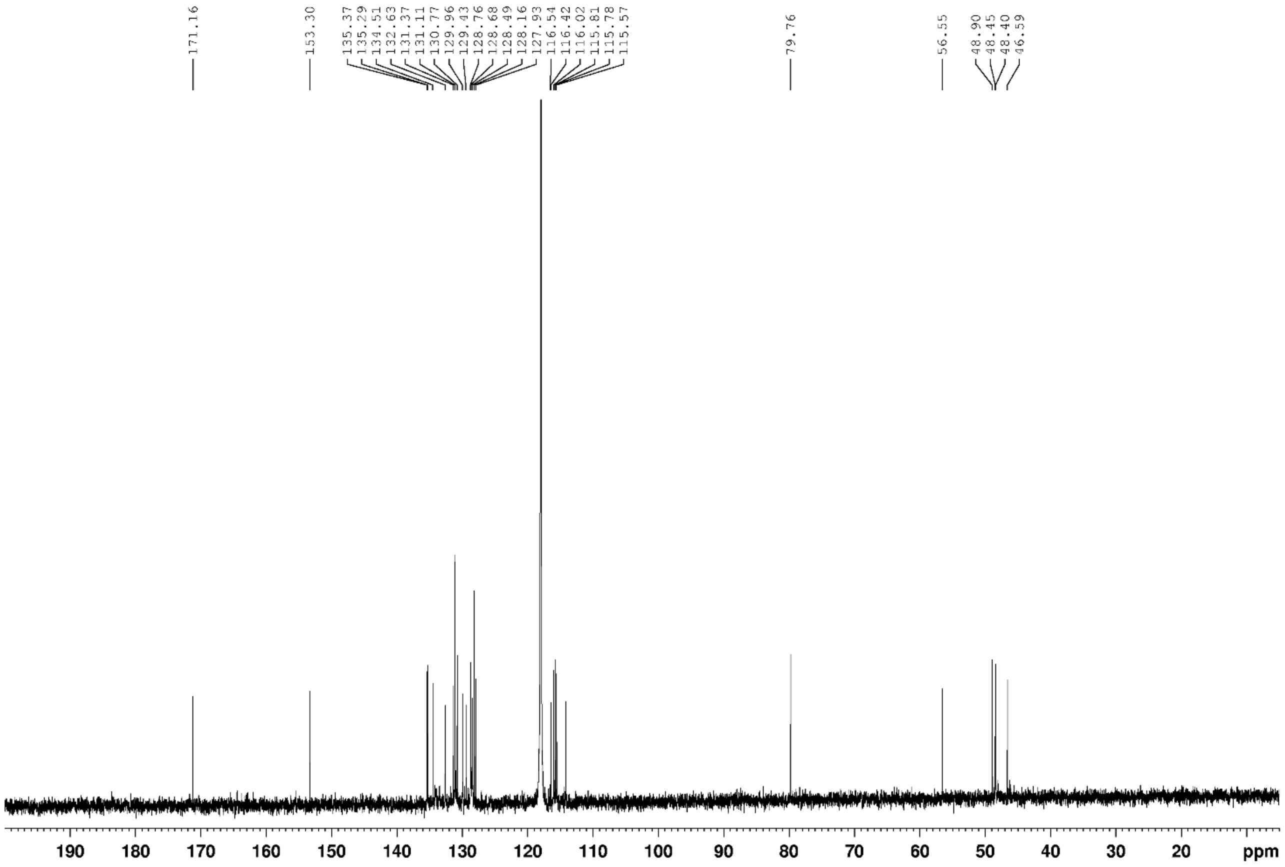

^{1}H NMR spectrum of compound 3h (CD_3CN, 400 MHz)

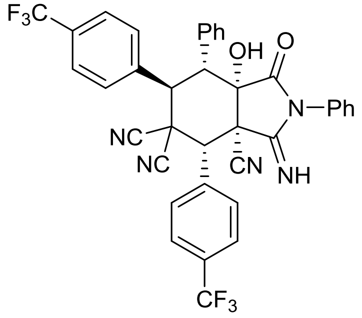

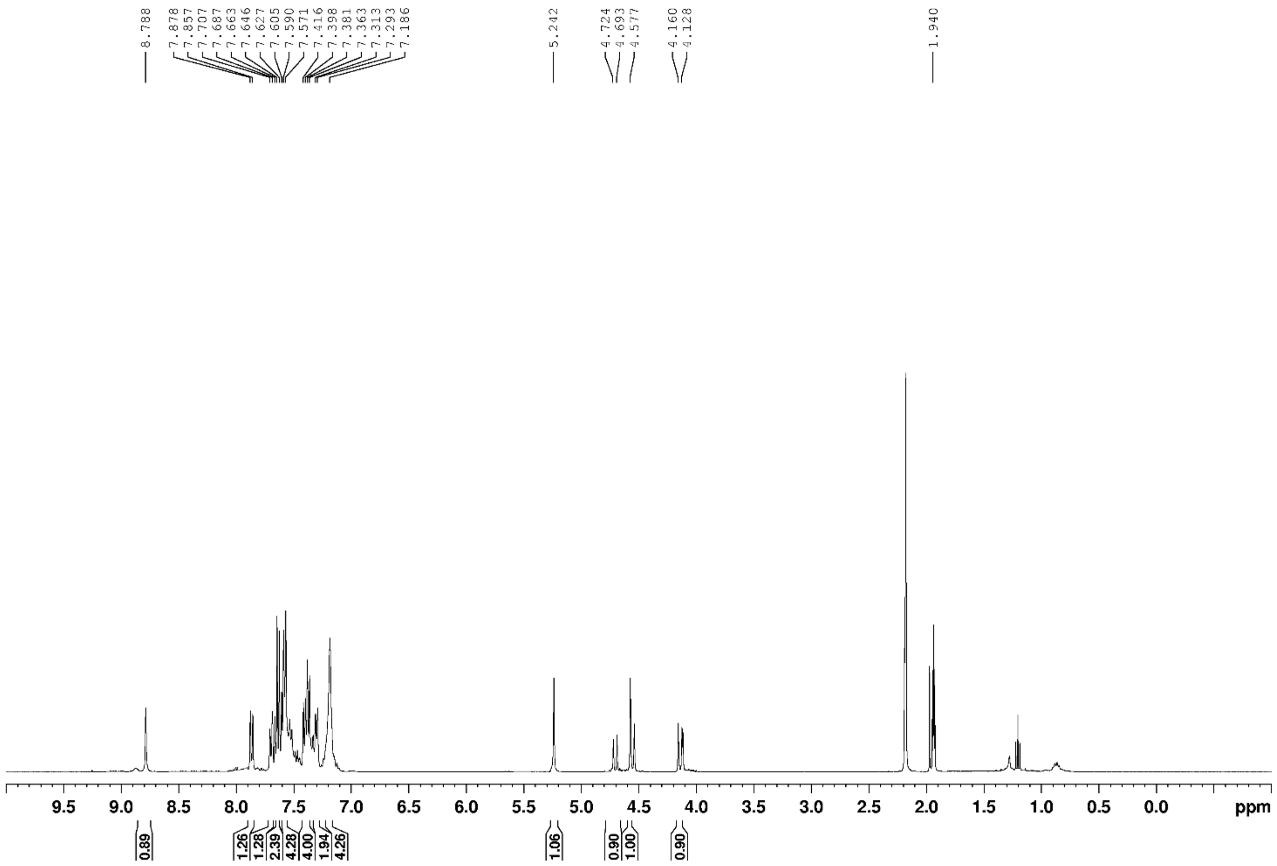

^{13}C NMR spectrum of compound 3h (CD_3CN, 100 MHz)

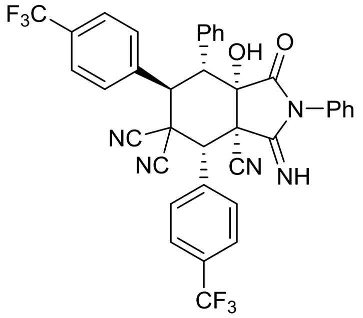

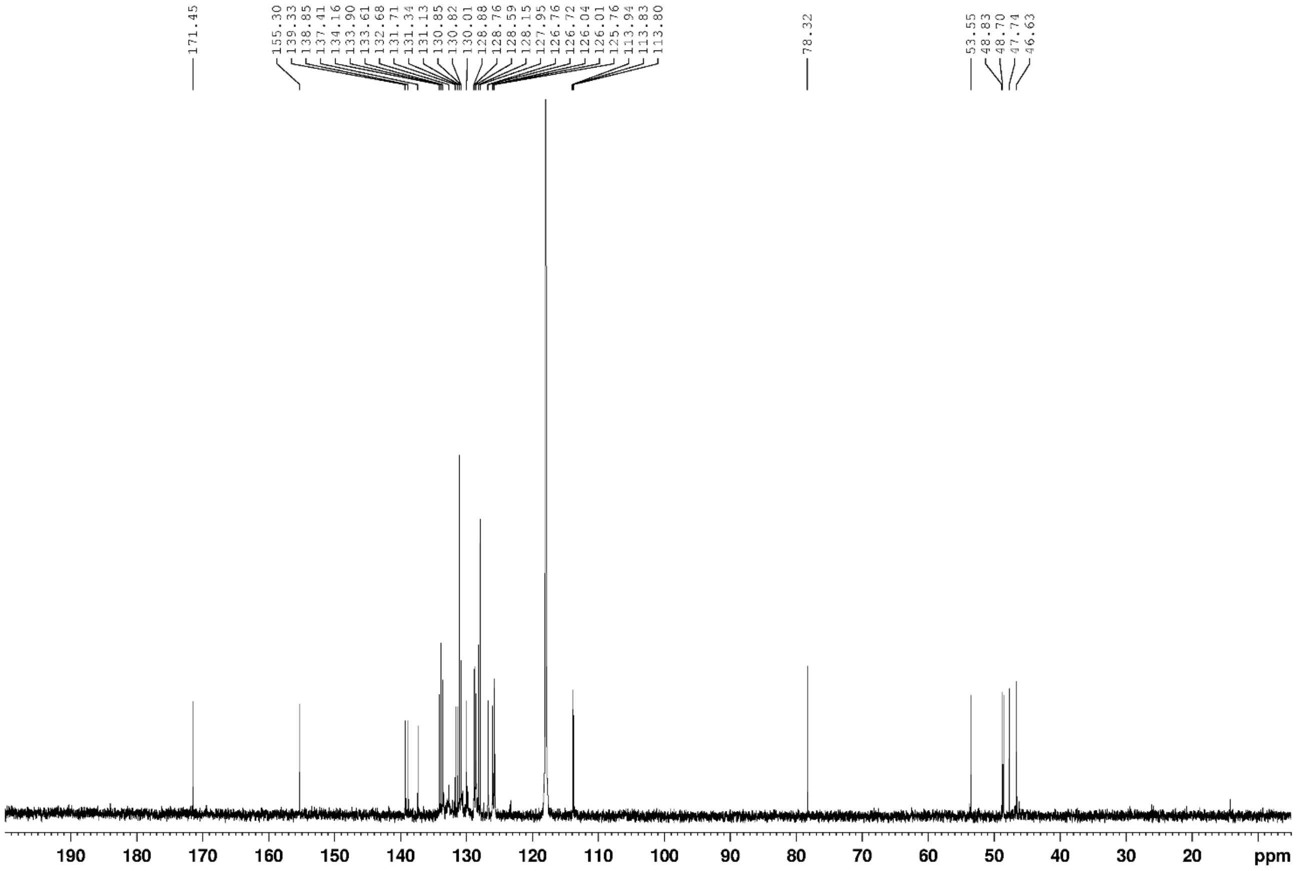

^{1}H NMR spectrum of compound 3i (CD_3CN, 400 MHz)

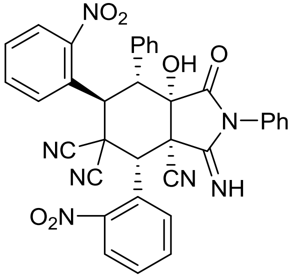

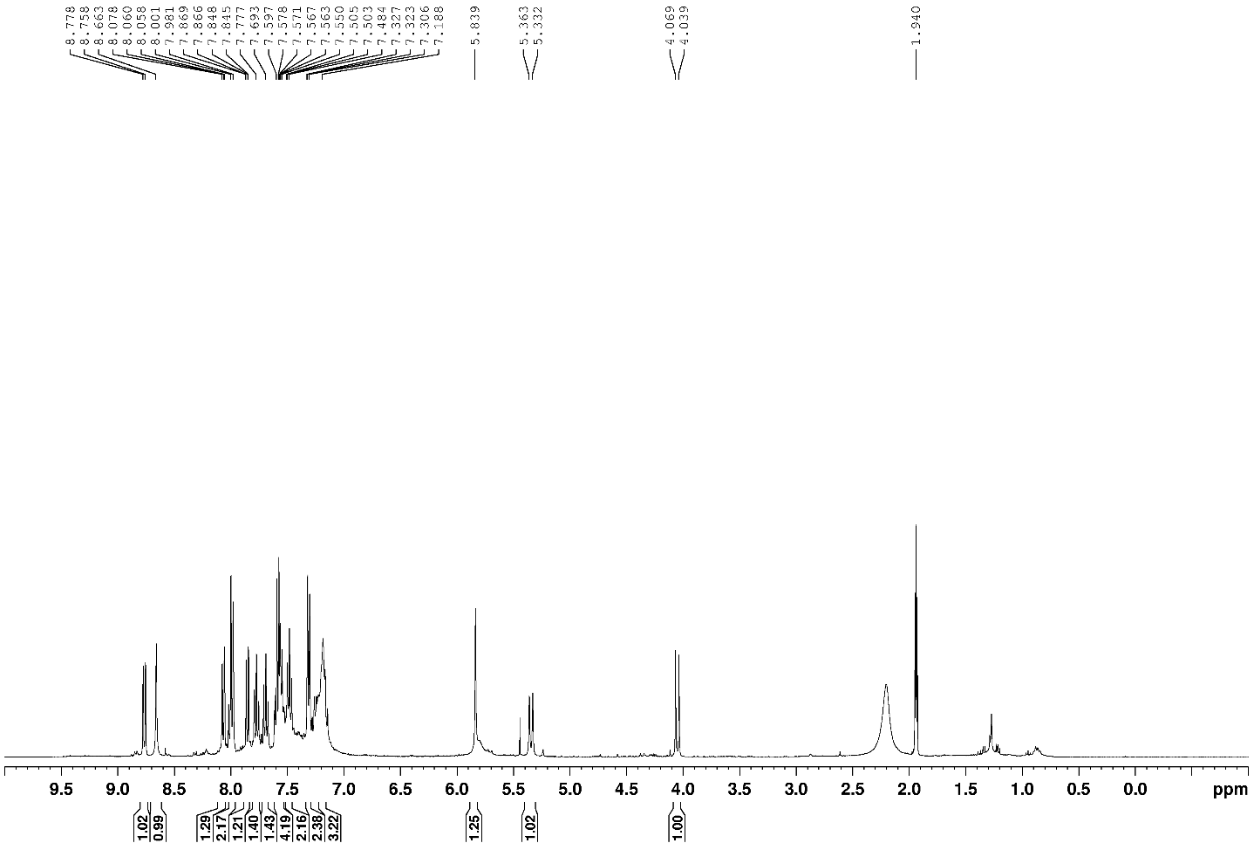

^{13}C NMR spectrum of compound 3i (CD_3CN, 100 MHz)

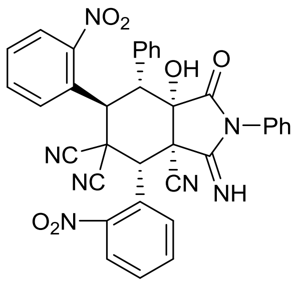

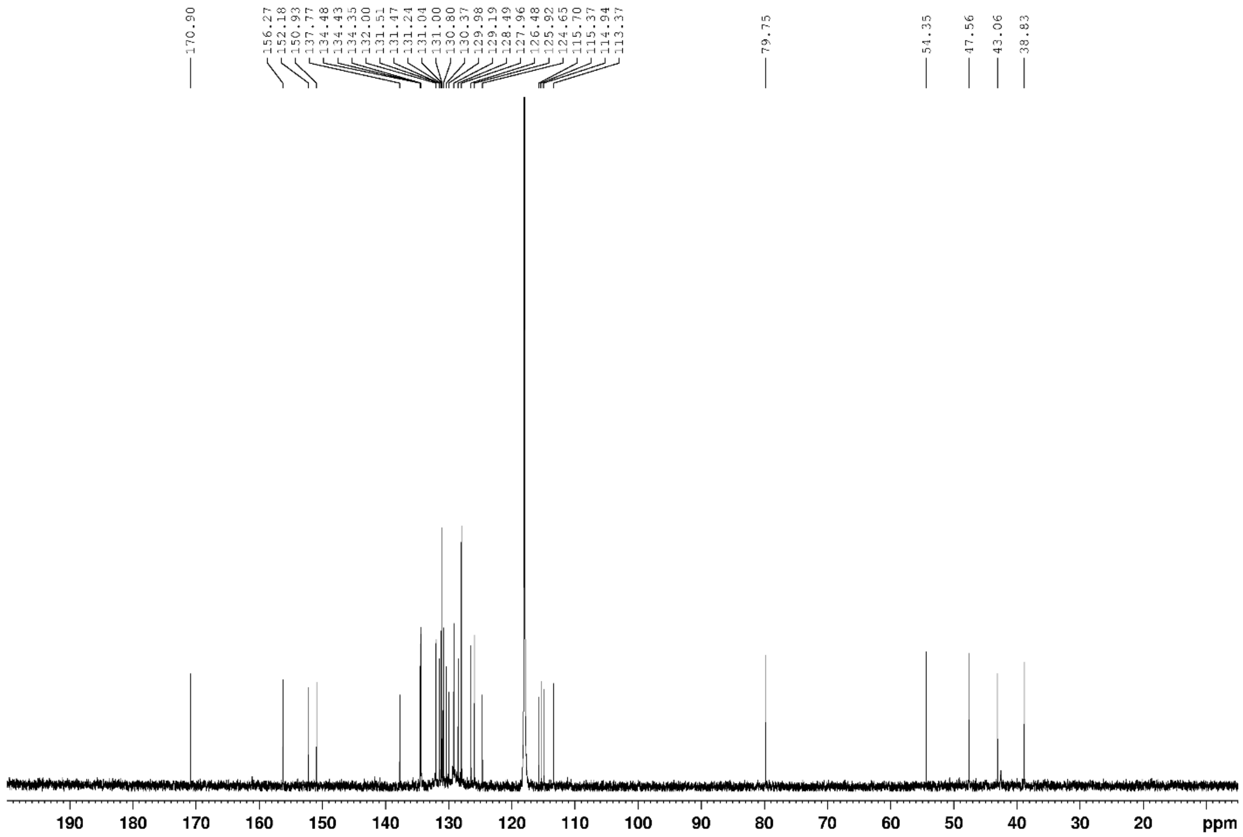

^{1}H NMR spectrum of compound 3j (CD_3CN, 400 MHz)

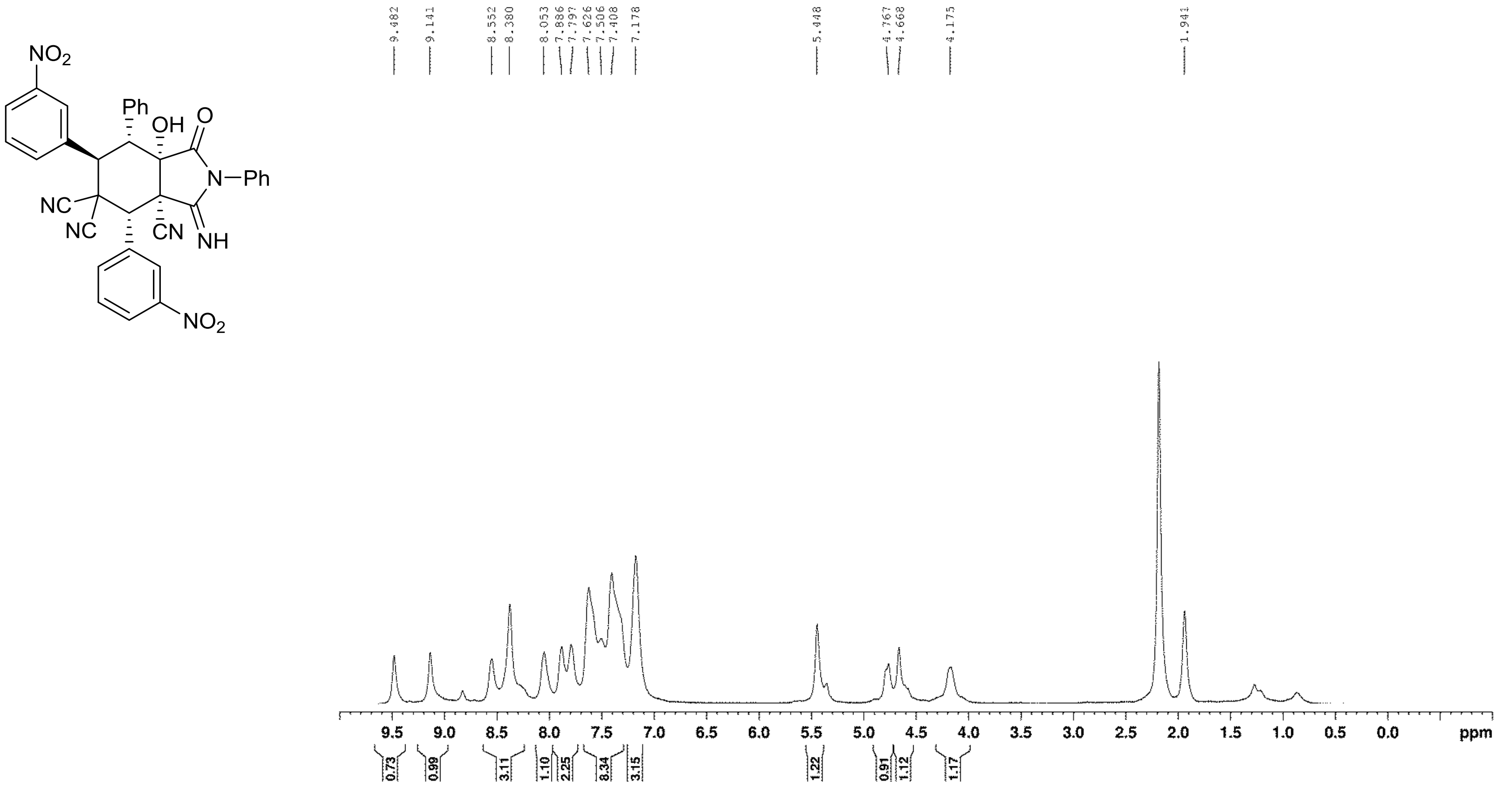

^{13}C NMR spectrum of compound 3j (CD_3CN, 100 MHz)

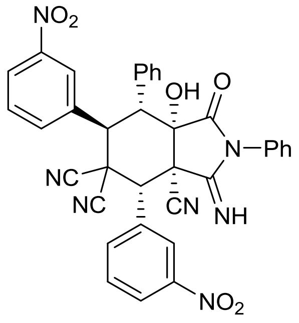

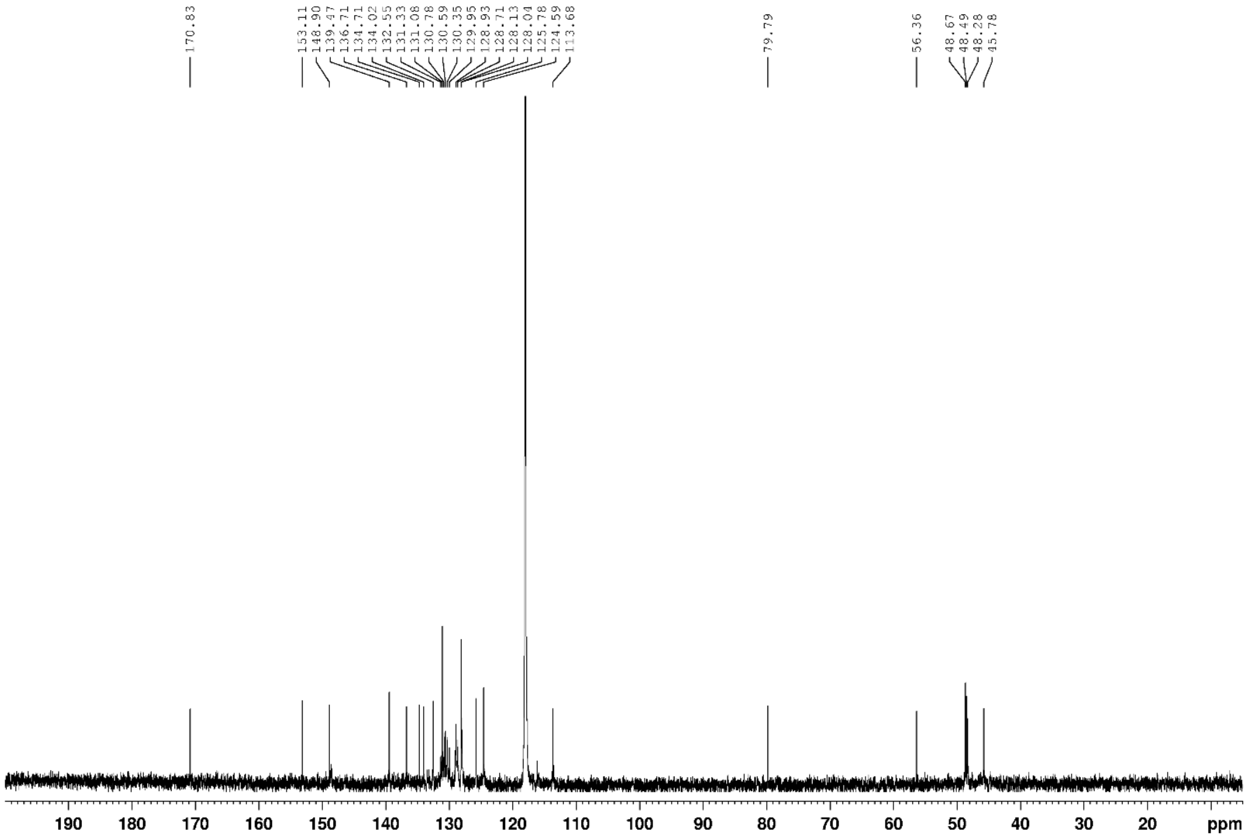

^{1}H NMR spectrum of compound 3k (d_6-DMSO, 400 MHz)

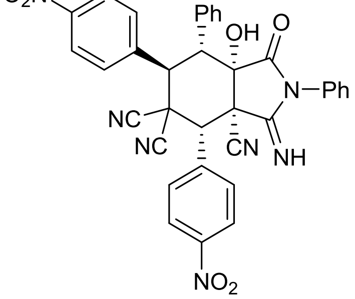

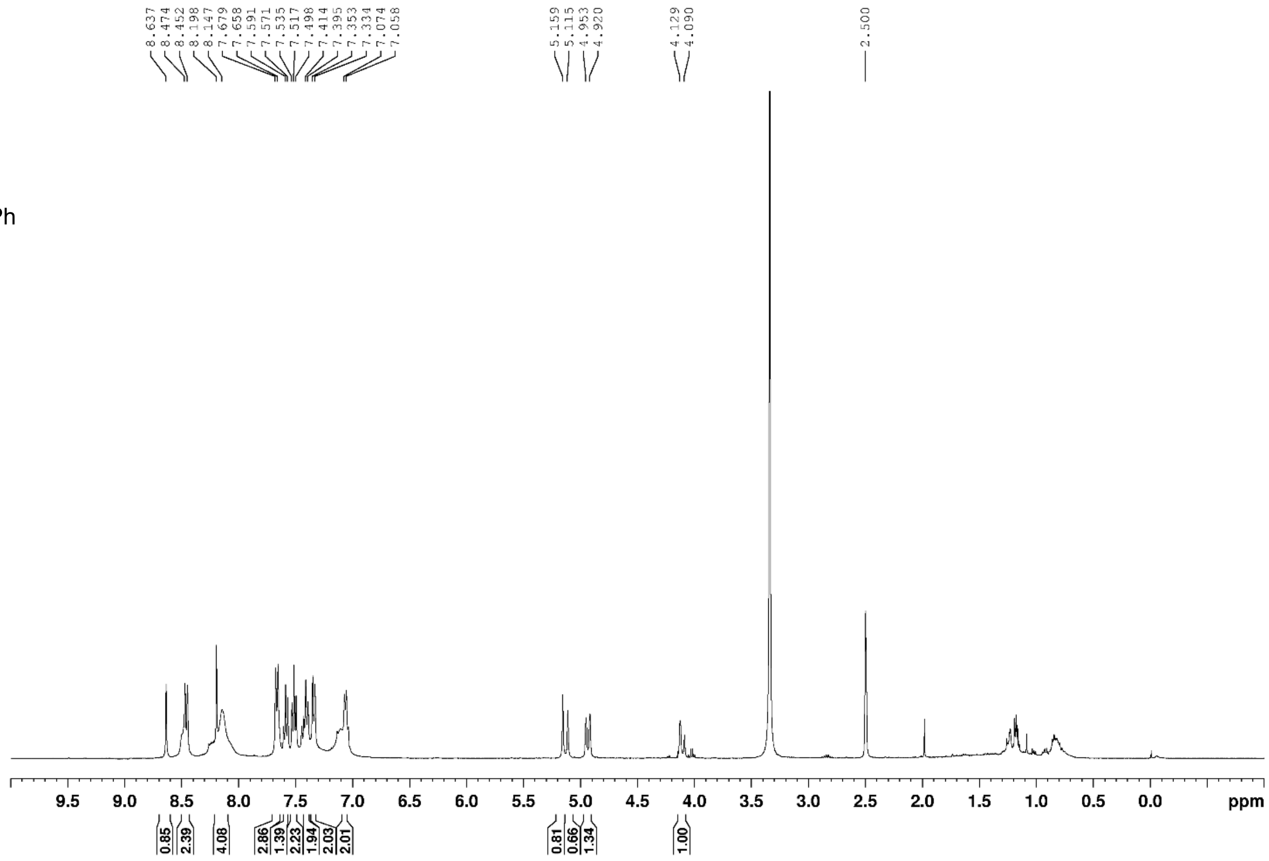

^{13}C NMR spectrum of compound 3k (d_6-DMSO, 100 MHz)

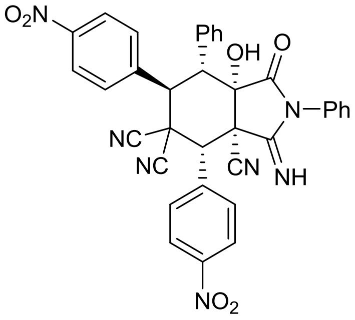

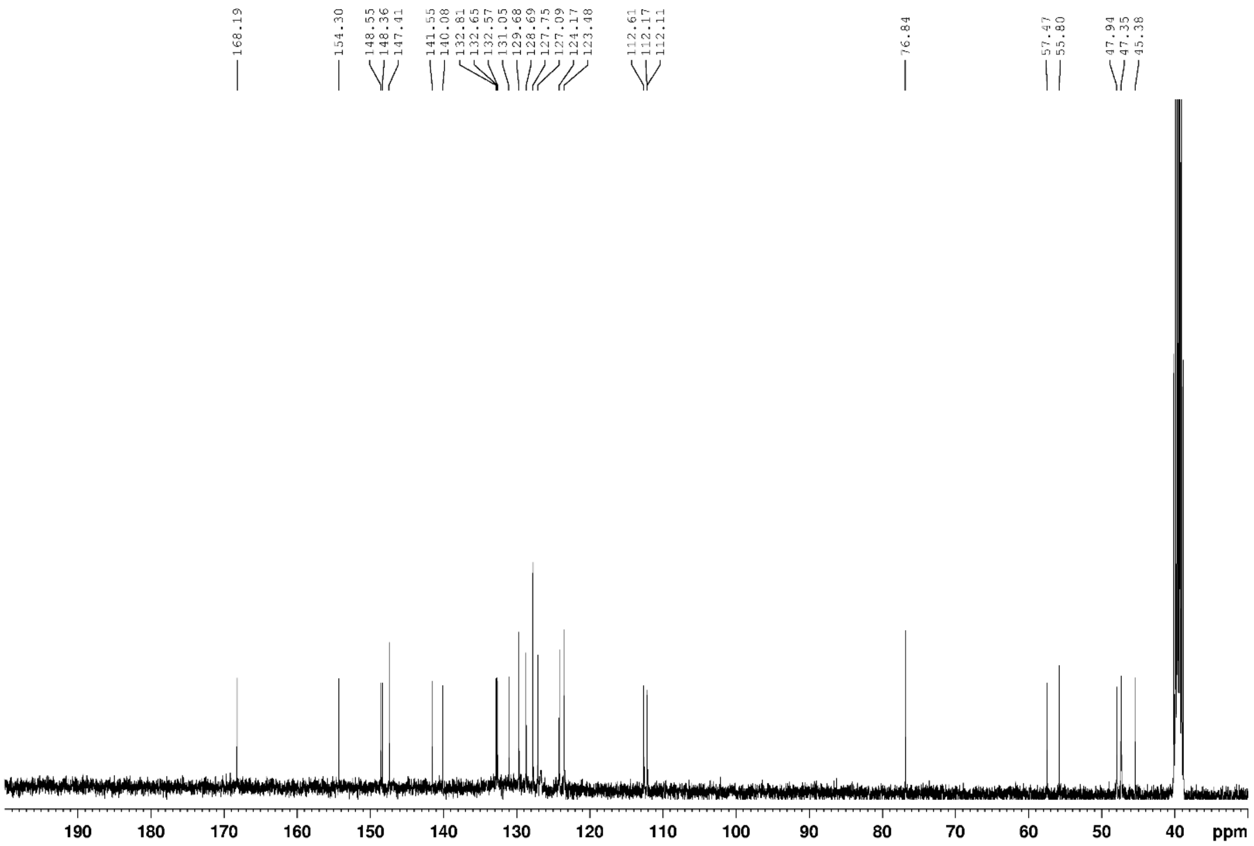

^{1}H NMR spectrum of compound 3l (CD_3CN, 400 MHz)

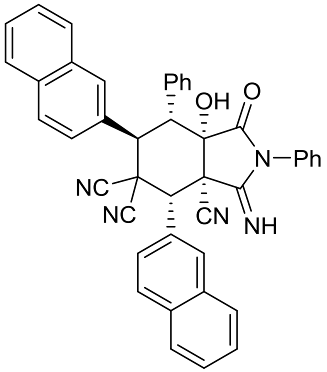

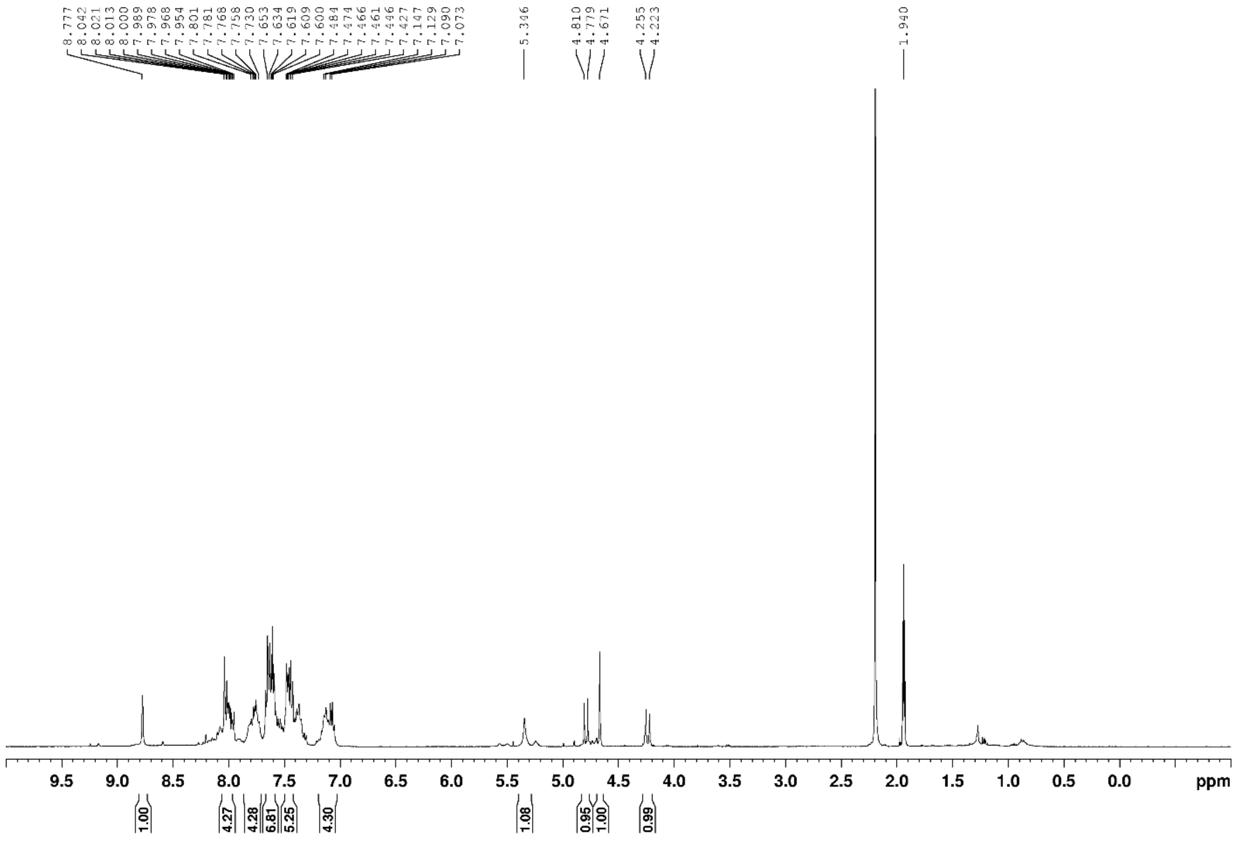

^{13}C NMR spectrum of compound 3l (CD_3CN, 100 MHz)

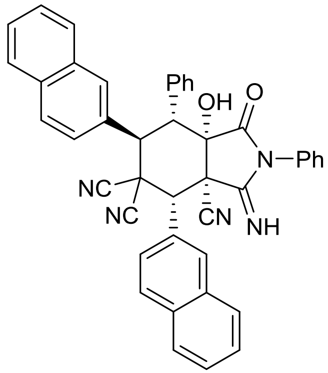

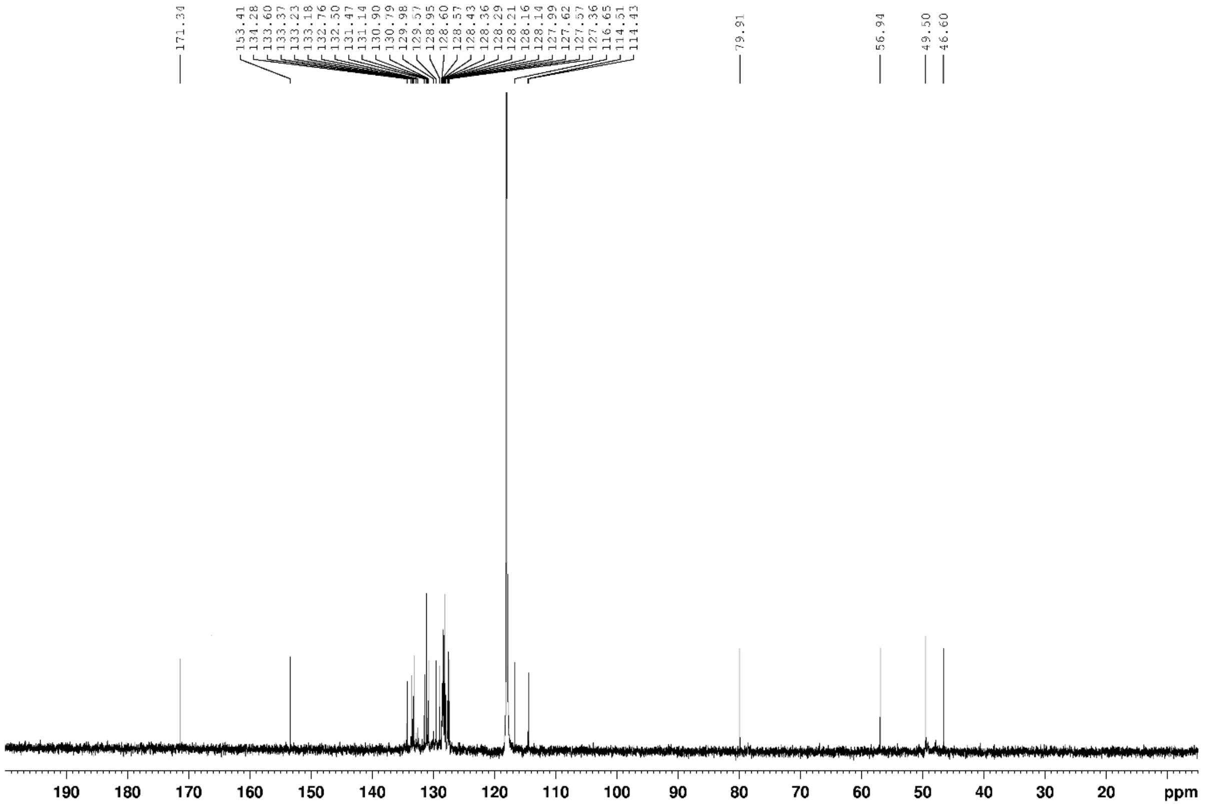

^{1}H NMR spectrum of compound 3m (CD_3CN, 400 MHz)

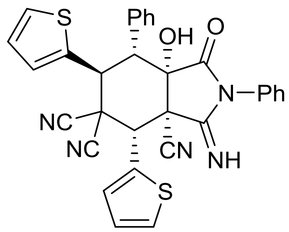

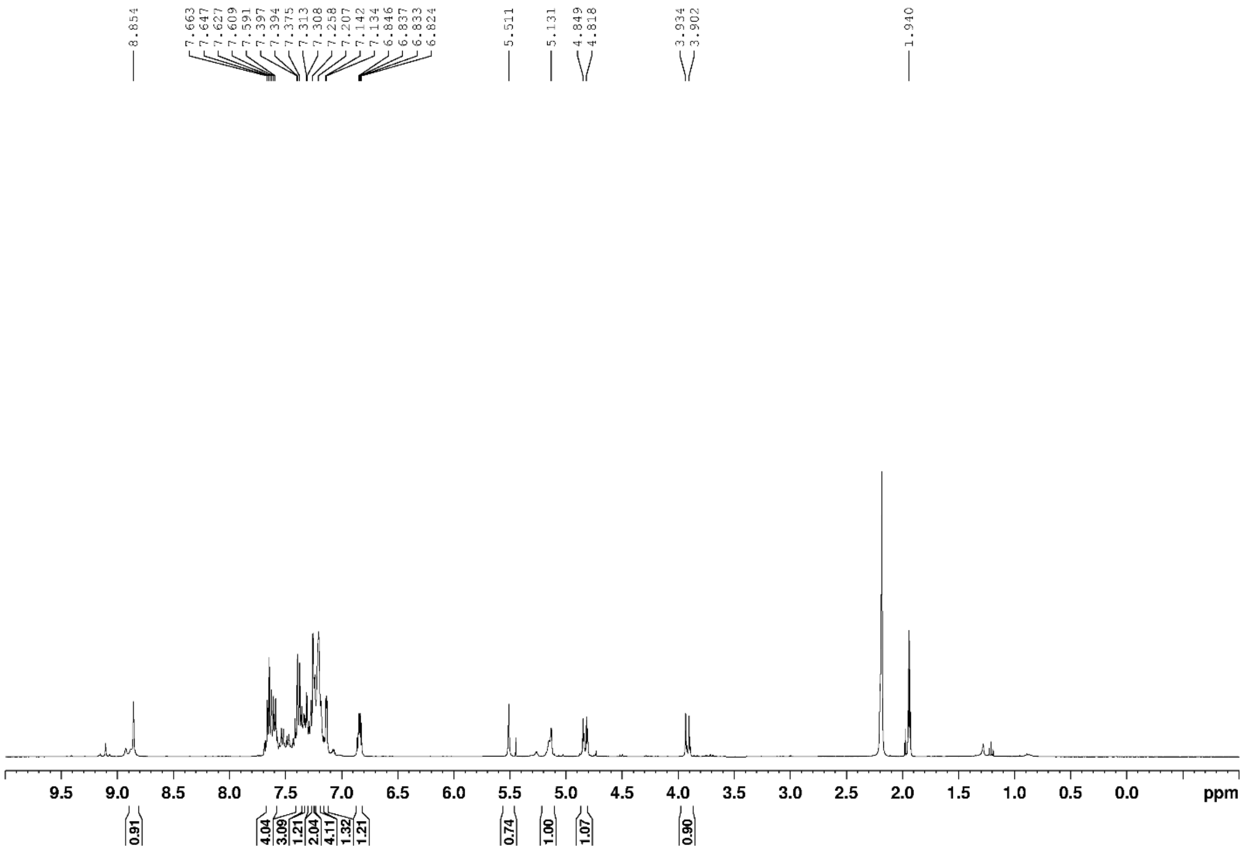

^{13}C NMR spectrum of compound 3m (CD_3CN, 100 MHz)

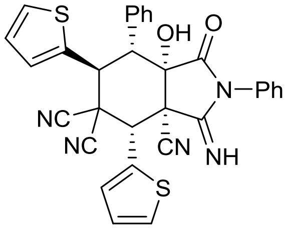

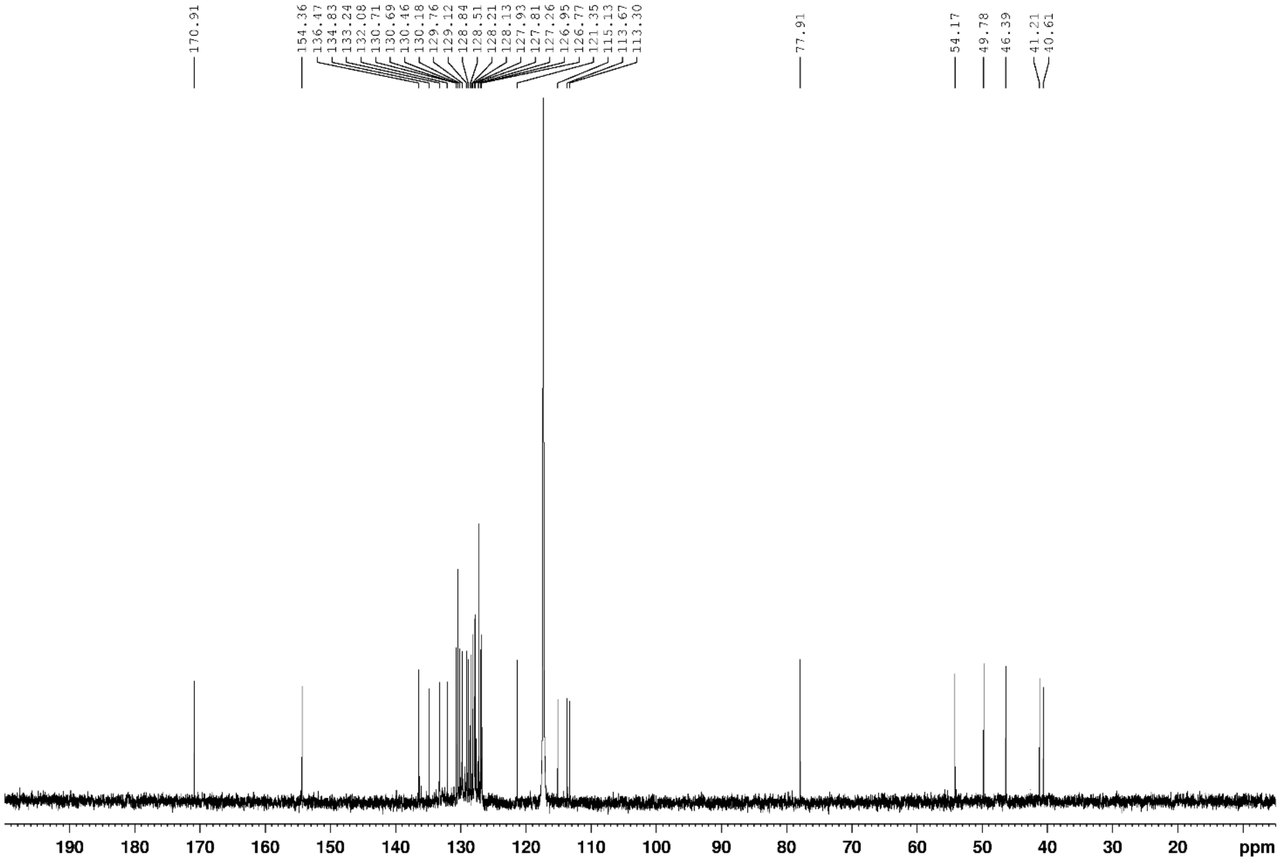

^{1}H NMR spectrum of compound 3n (CD_3CN, 400 MHz)

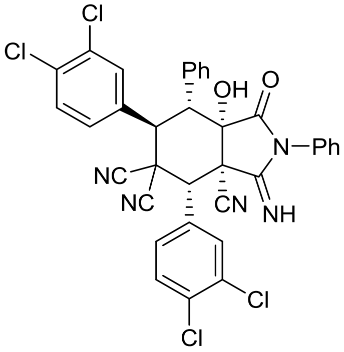

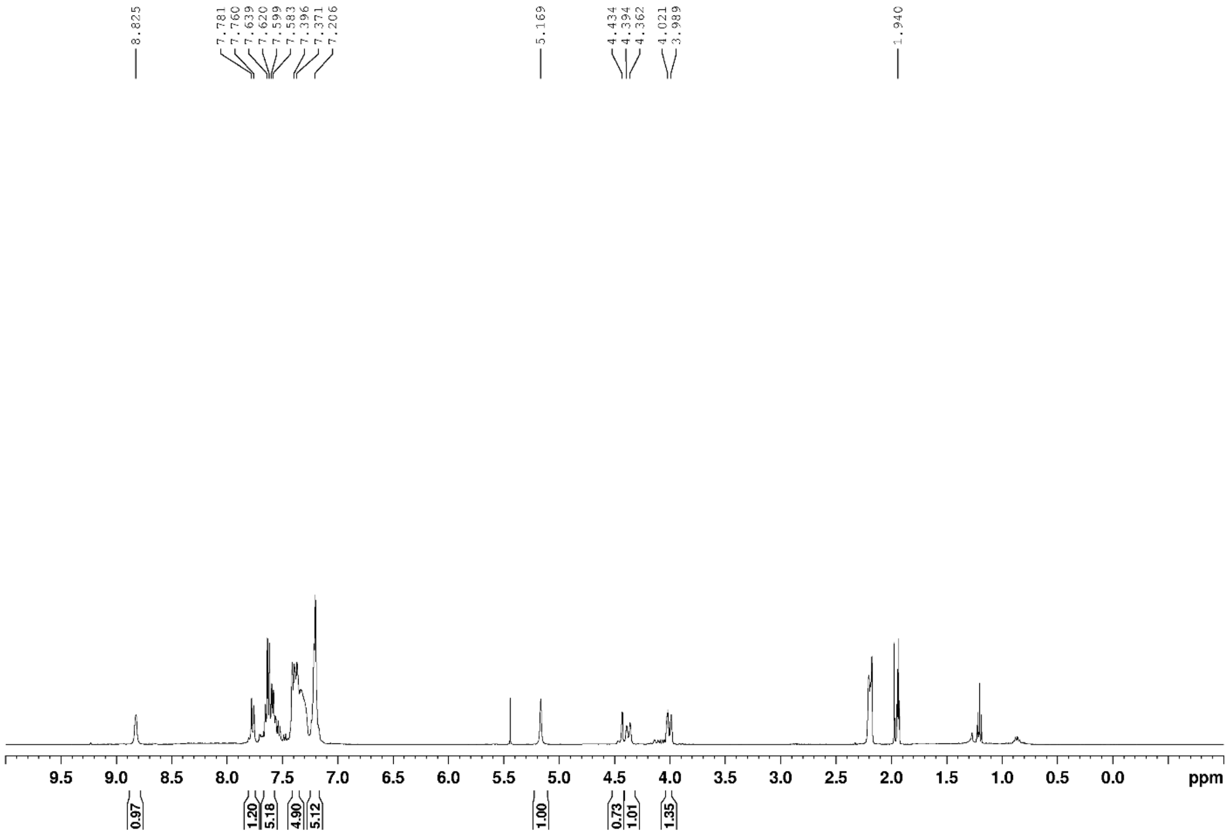

^{13}C NMR spectrum of compound 3n (CD_3CN, 100 MHz)

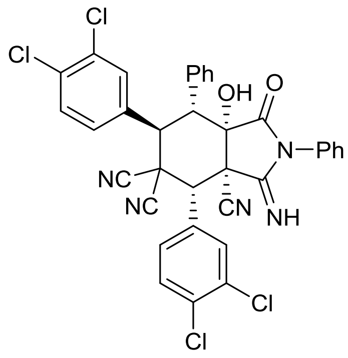

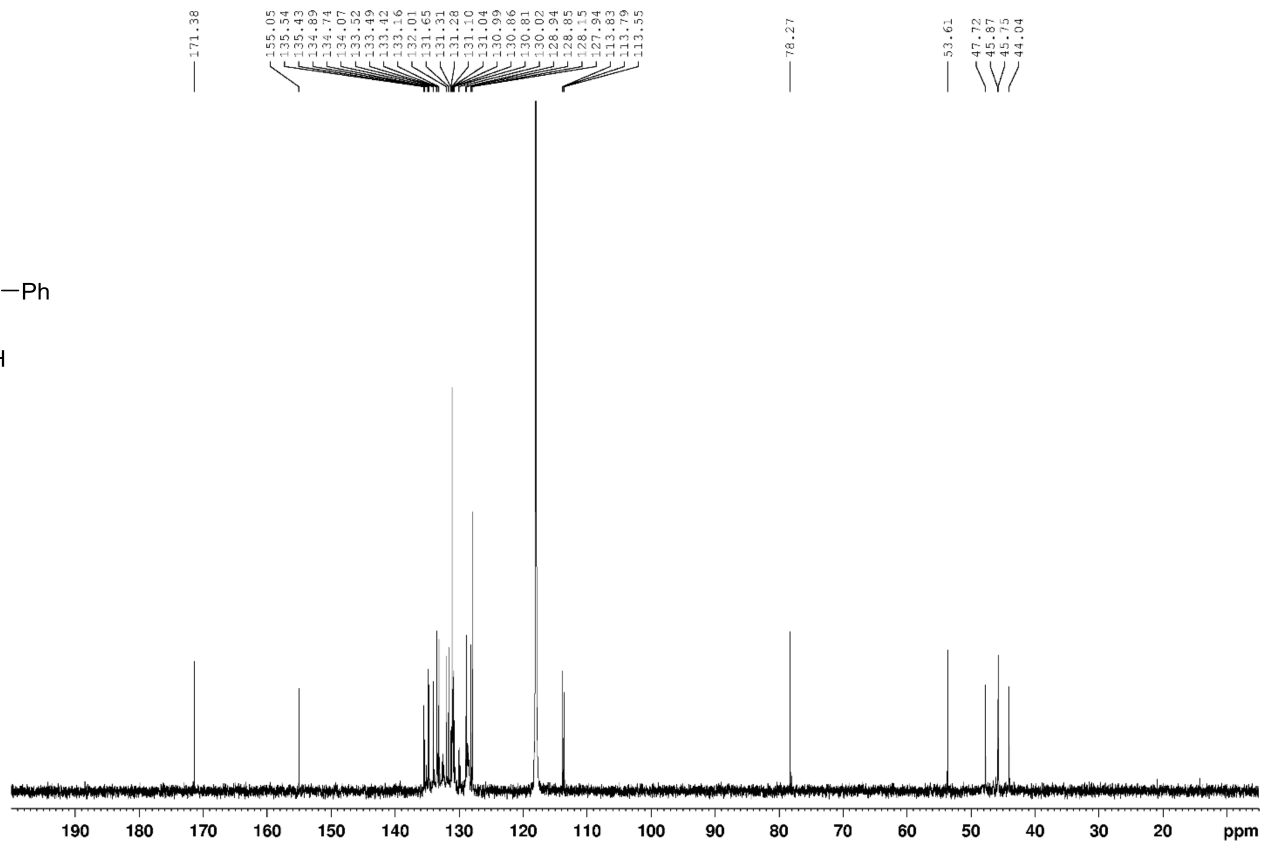

^{1}H NMR spectrum of compound 3o (CD_3CN, 400 MHz)

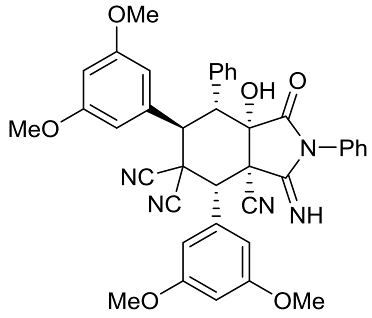

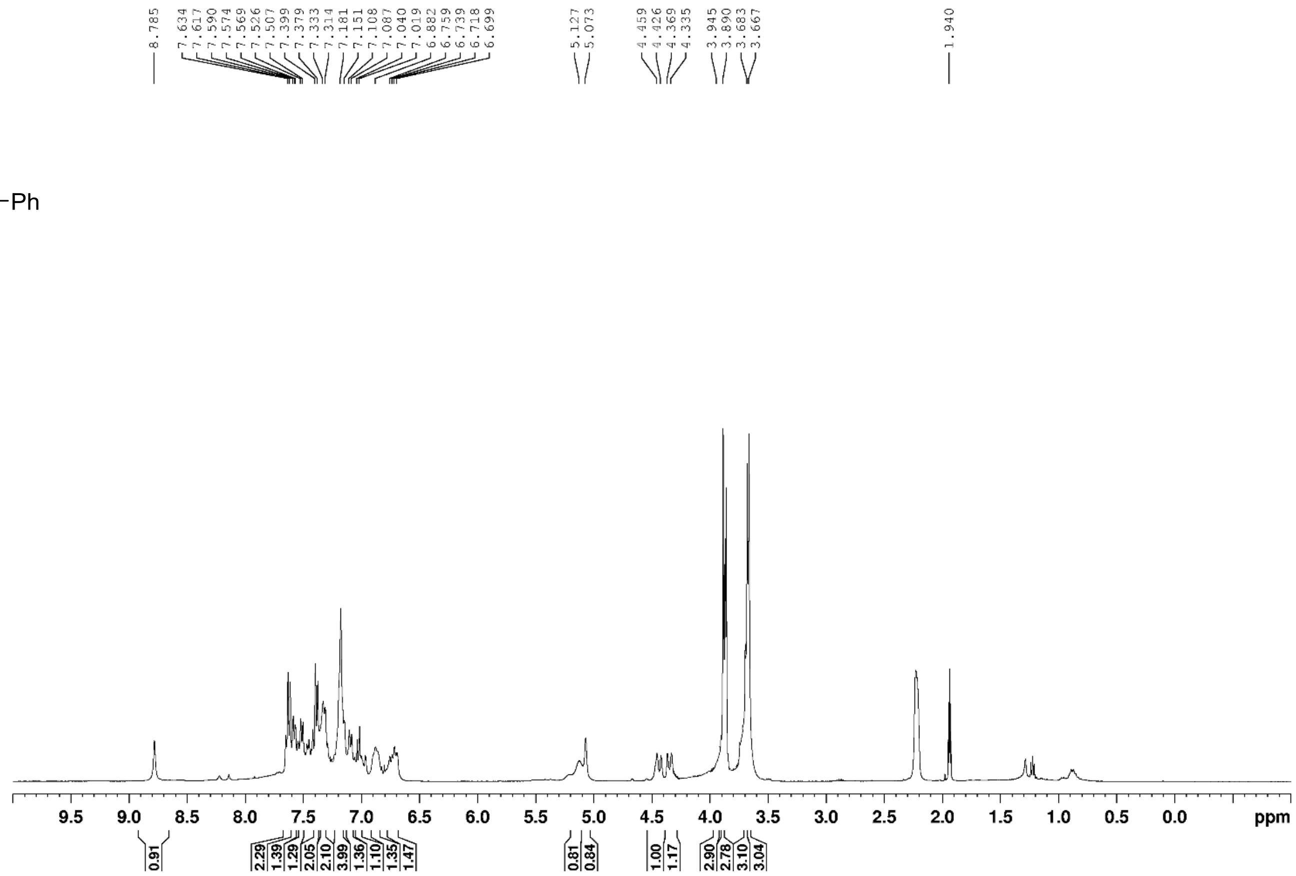

^{13}C NMR spectrum of compound 3o (CD_3CN, 100 MHz)

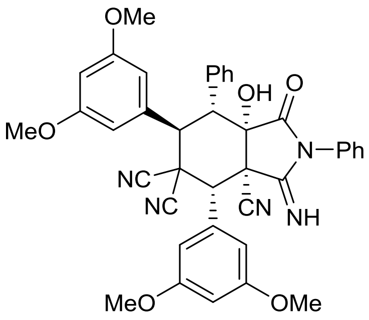

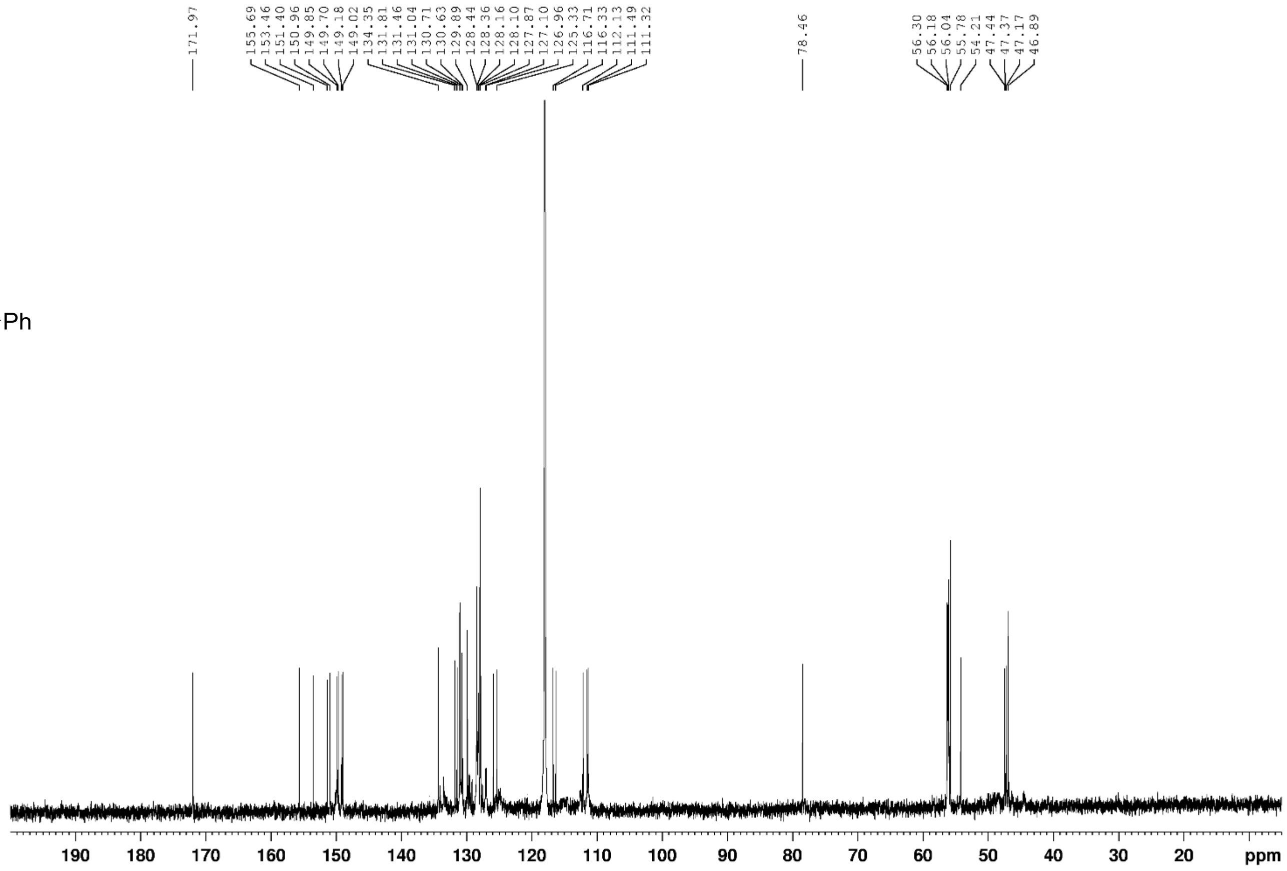

^{1}H NMR spectrum of compound 3p ($CDCl_3$, 400 MHz)

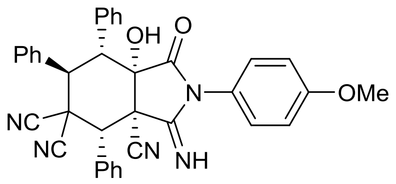

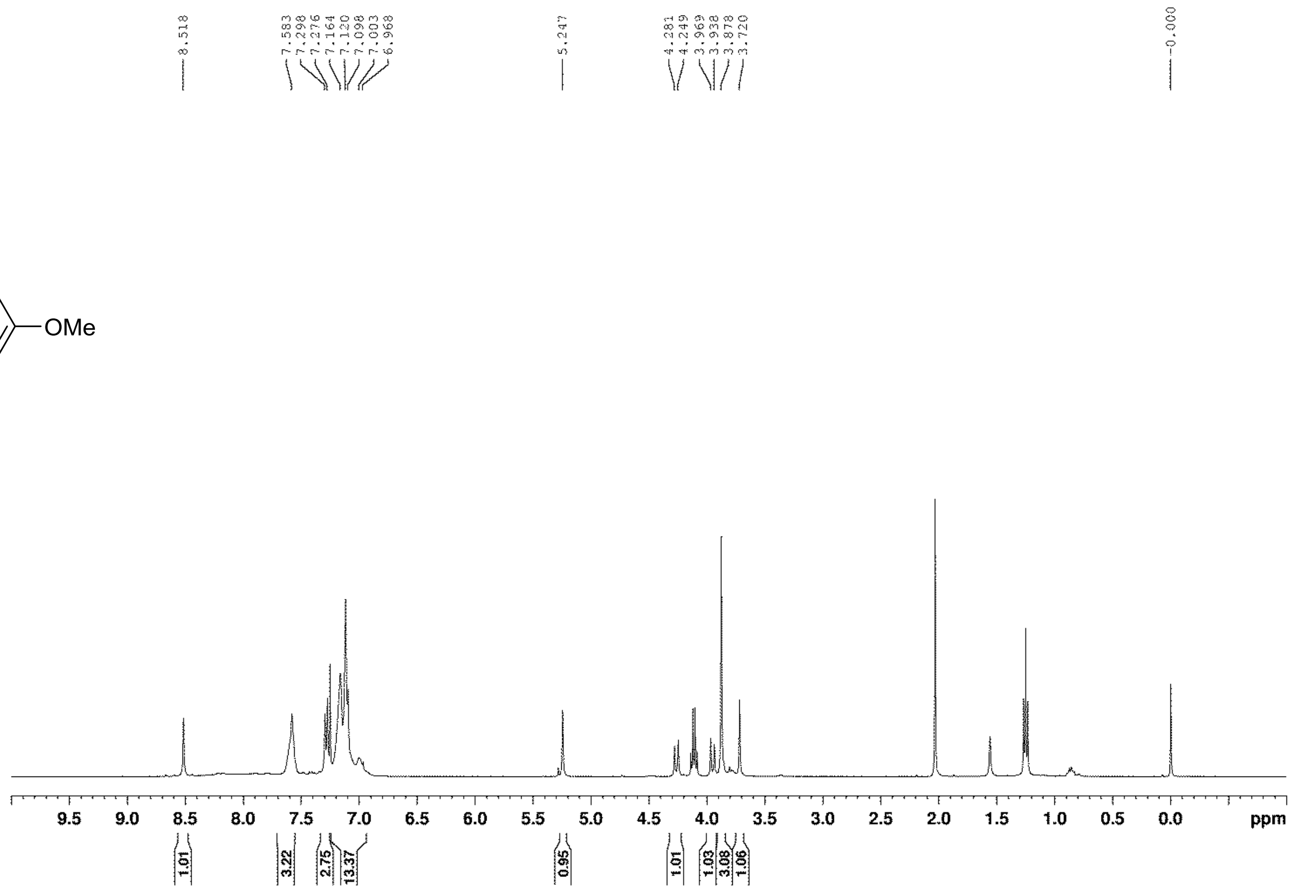

^{13}C NMR spectrum of compound 3p ($CDCl_3$, 100 MHz)

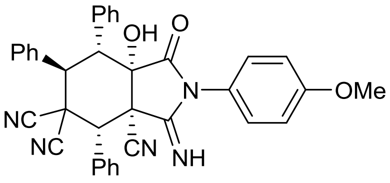

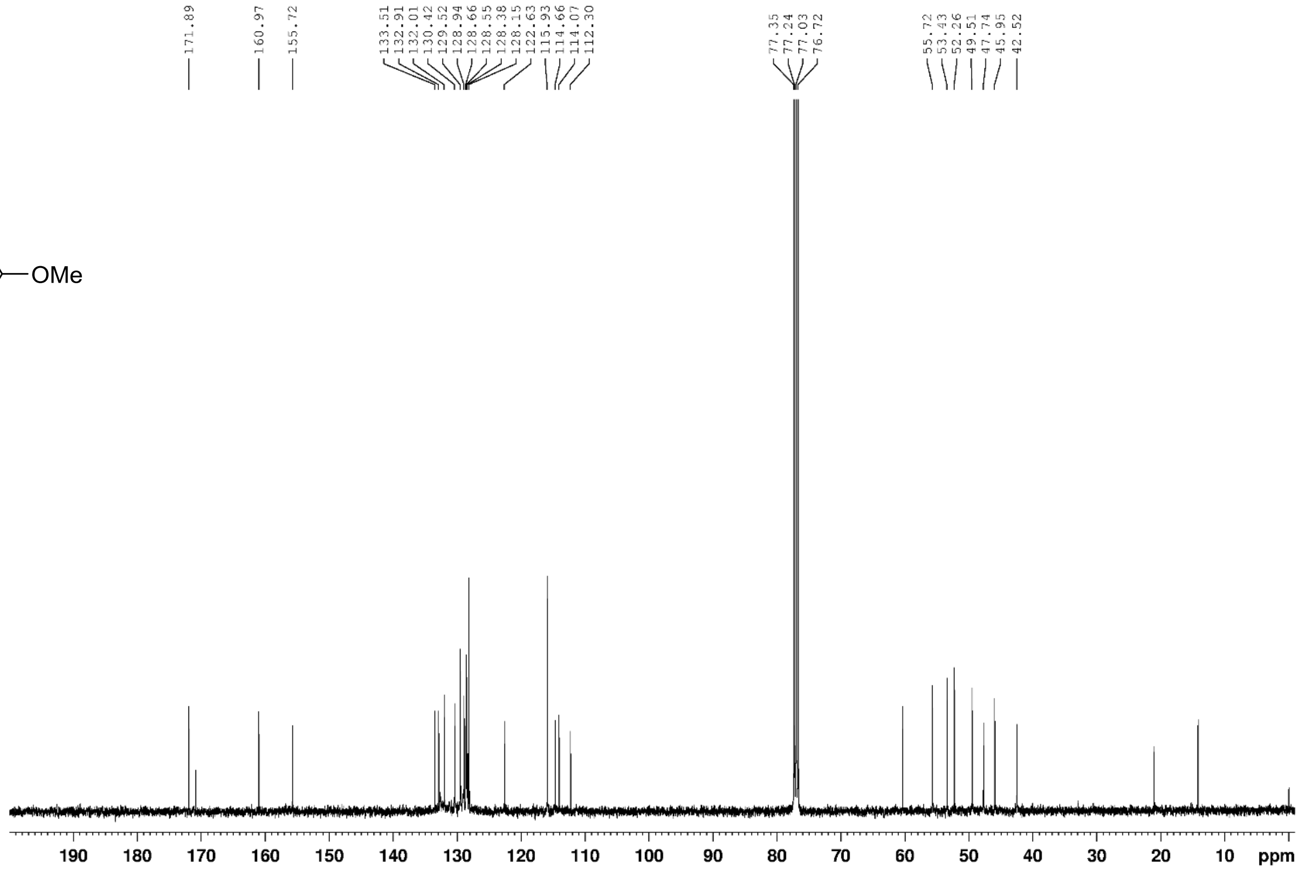

^{1}H NMR spectrum of compound 3q (CD_3CN, 400 MHz)

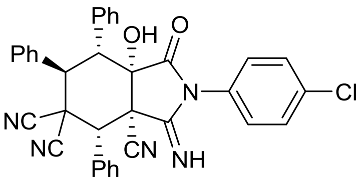

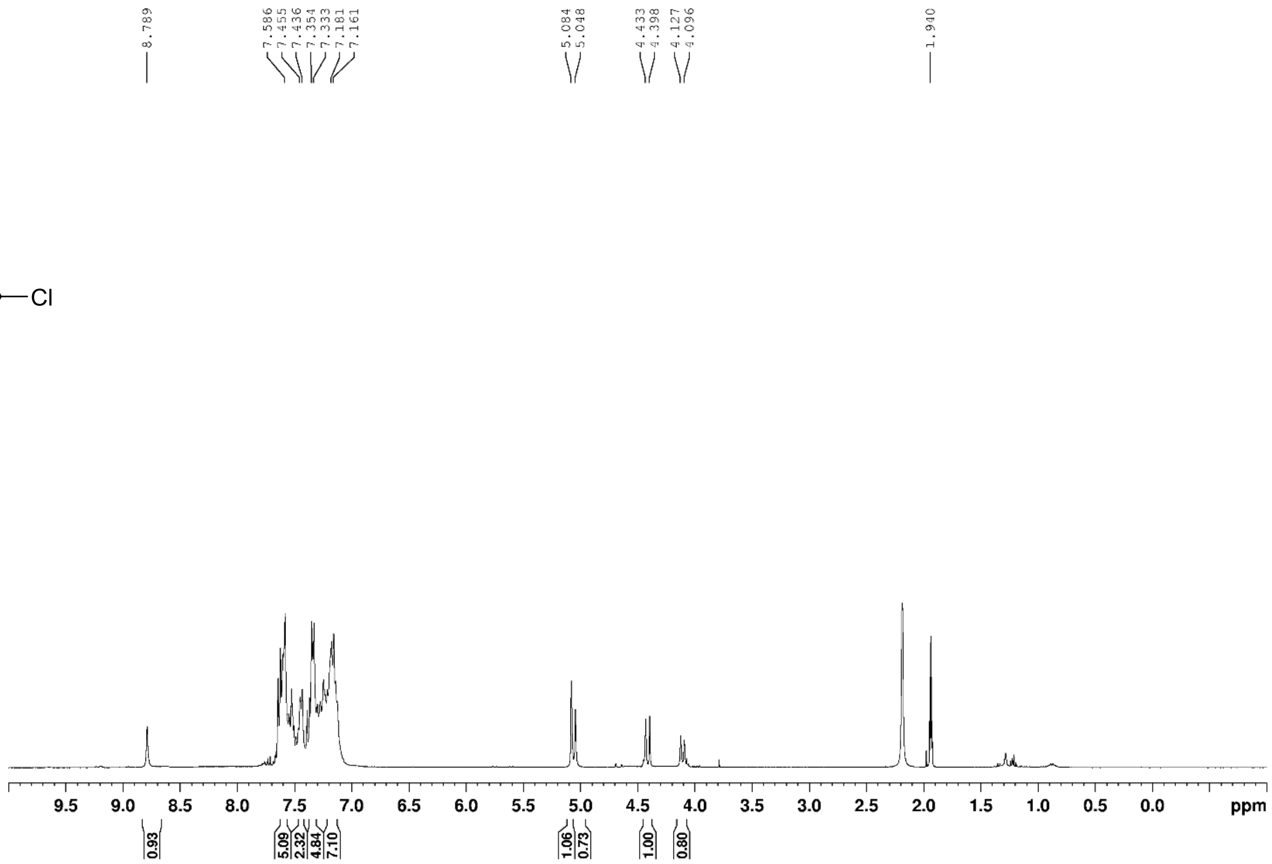

^{13}C NMR spectrum of compound 3q (CD_3CN, 100 MHz)

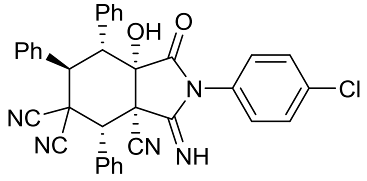

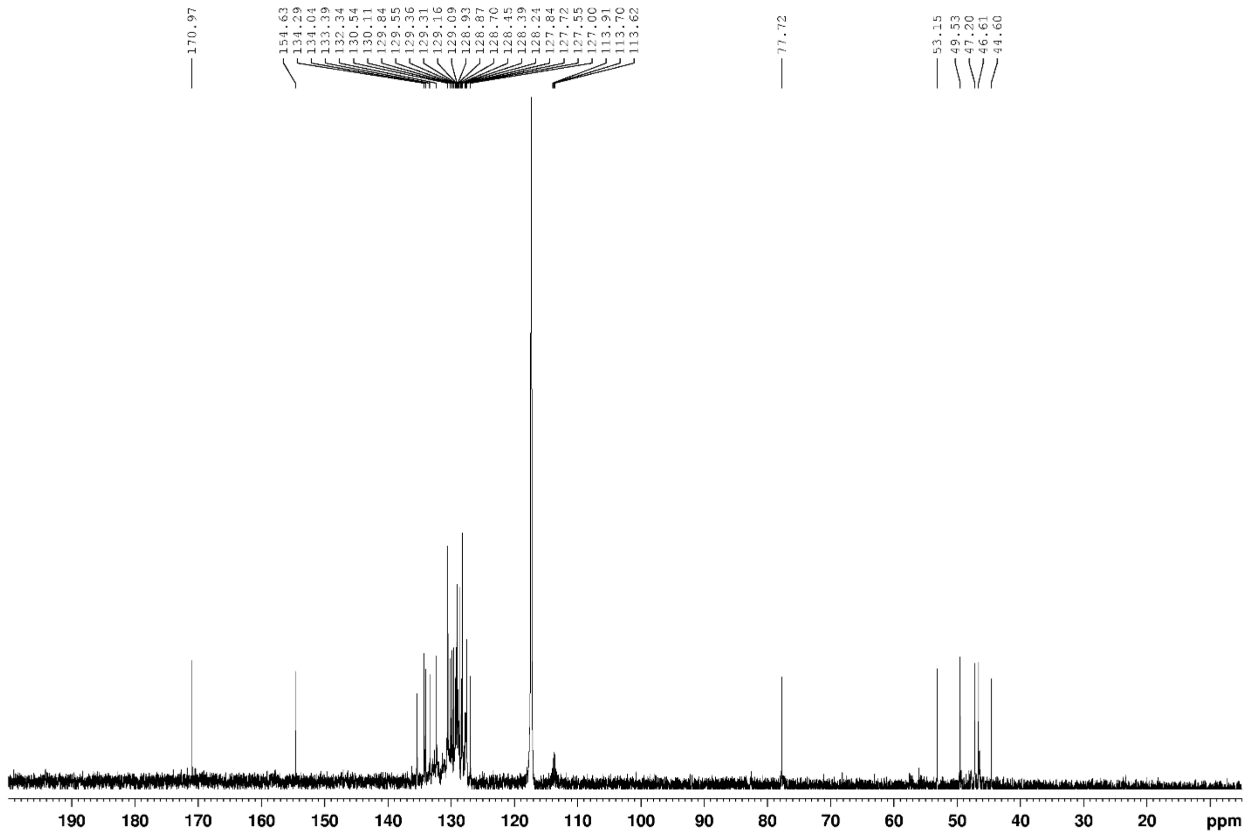

^{1}H NMR spectrum of compound 3r (CD_3CN, 400 MHz)

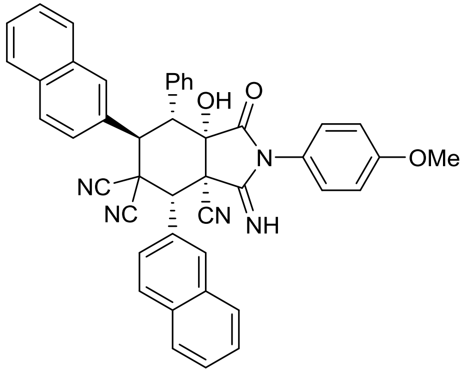

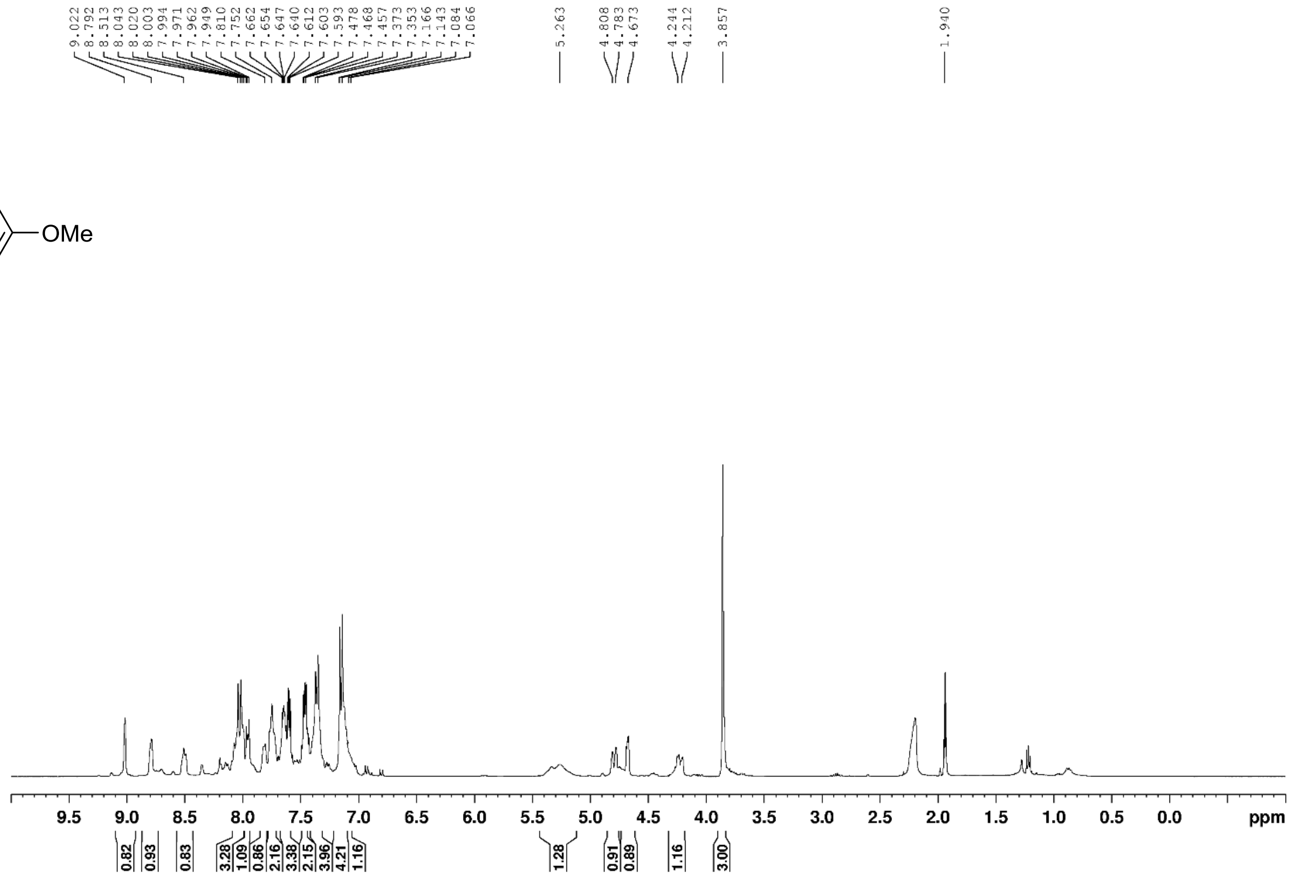

^{13}C NMR spectrum of compound 3r (CD_3CN, 100 MHz)

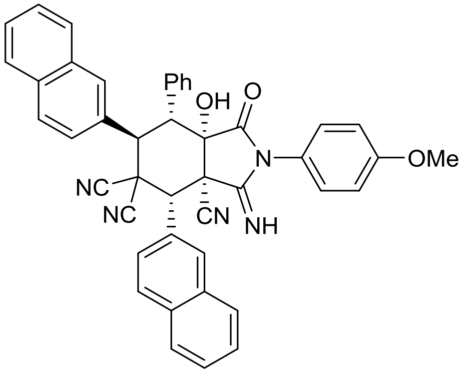

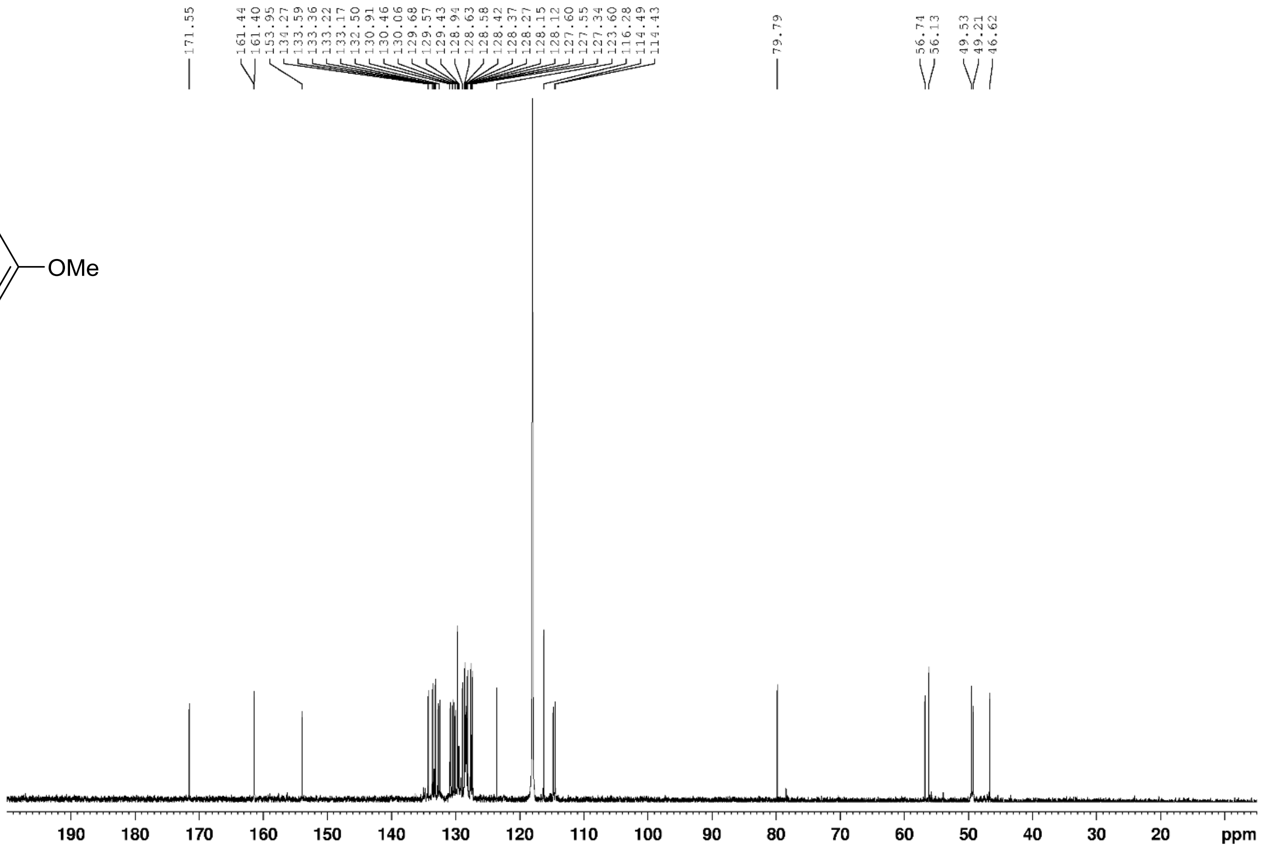

¹H NMR spectrum of compound 3s (CD_3CN, 400 MHz)

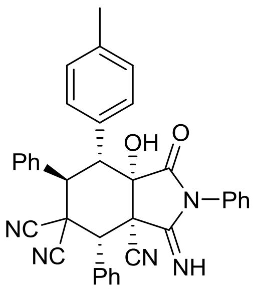

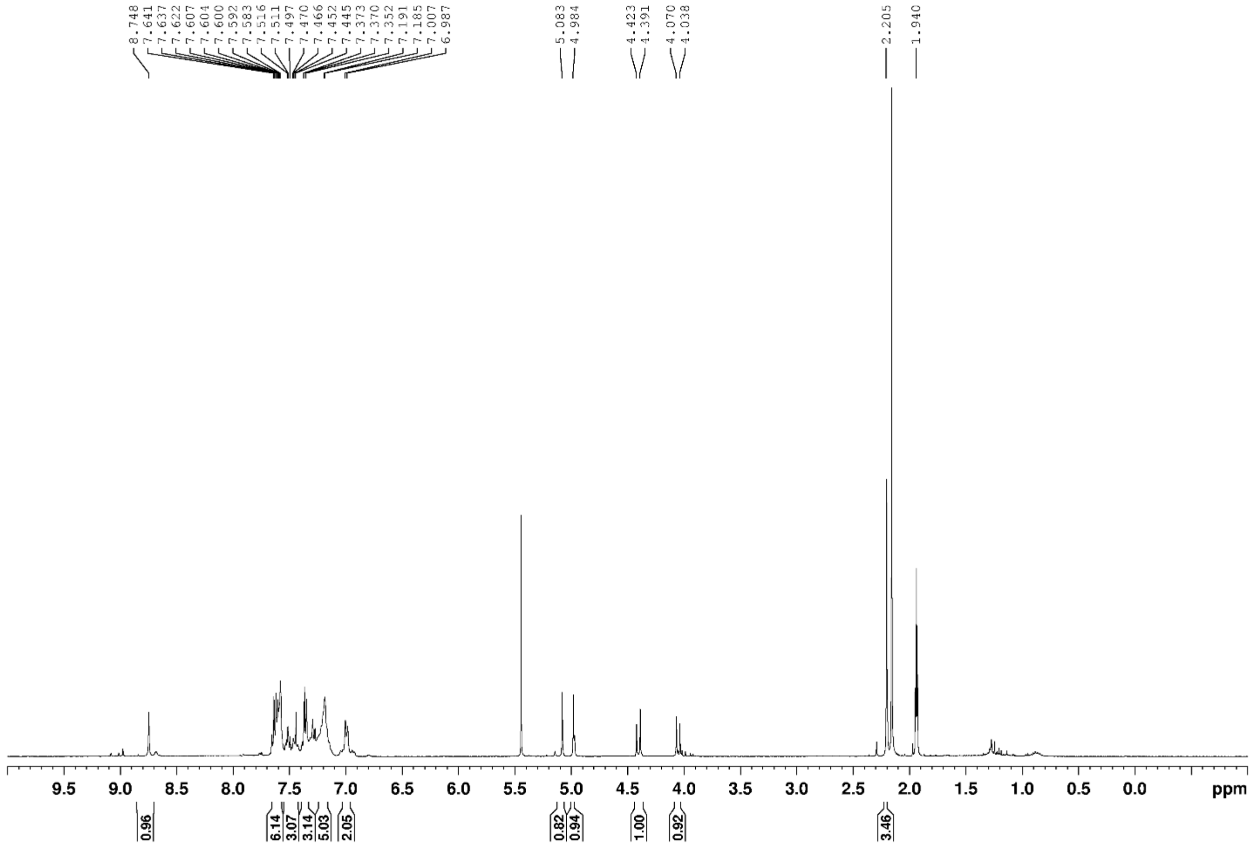

^{13}C NMR spectrum of compound 3s (CD_3CN, 100 MHz)

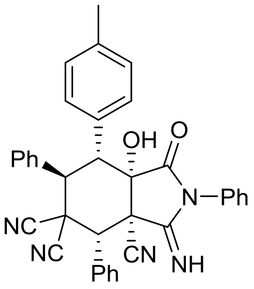

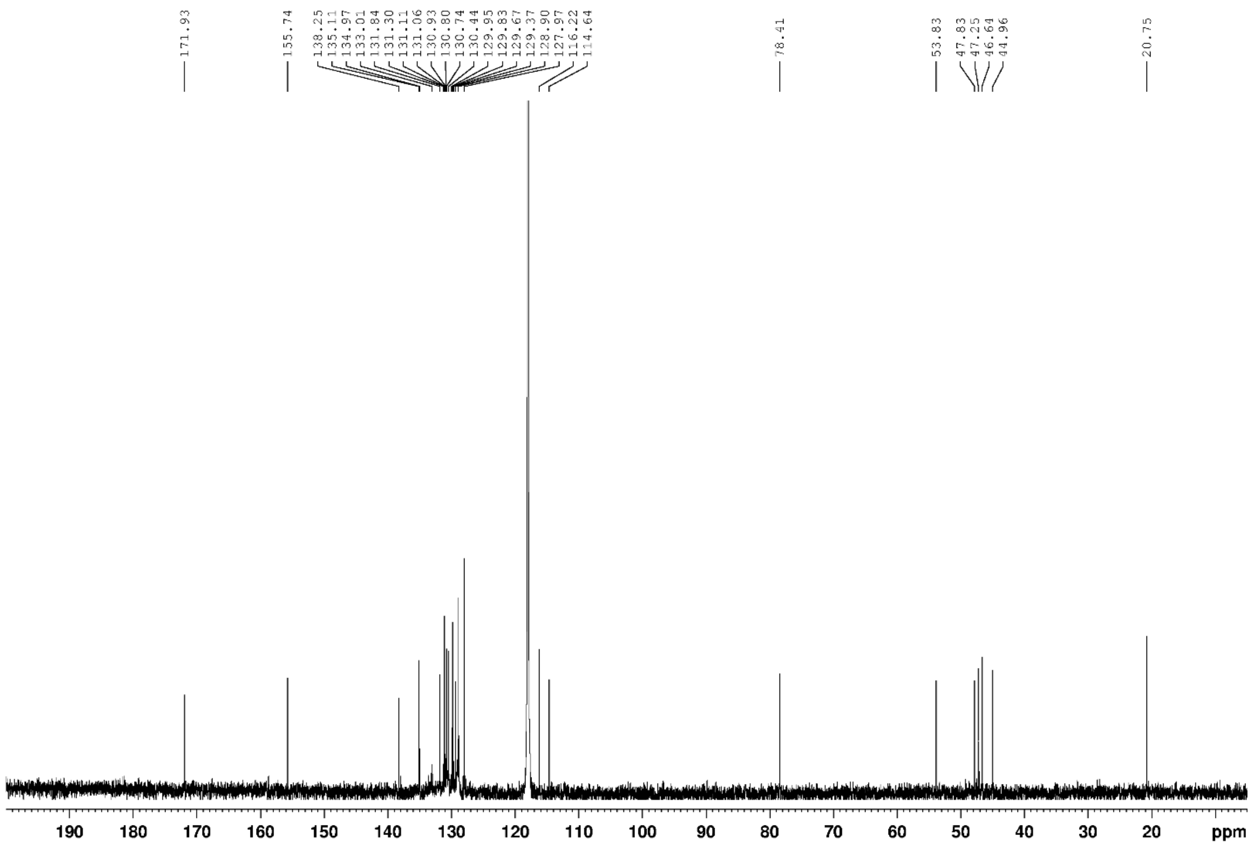

^{1}H NMR spectrum of compound 3t (CD_3CN, 400 MHz)

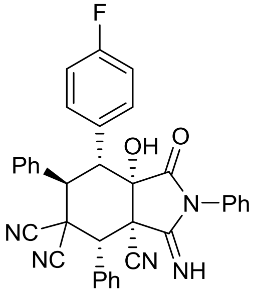

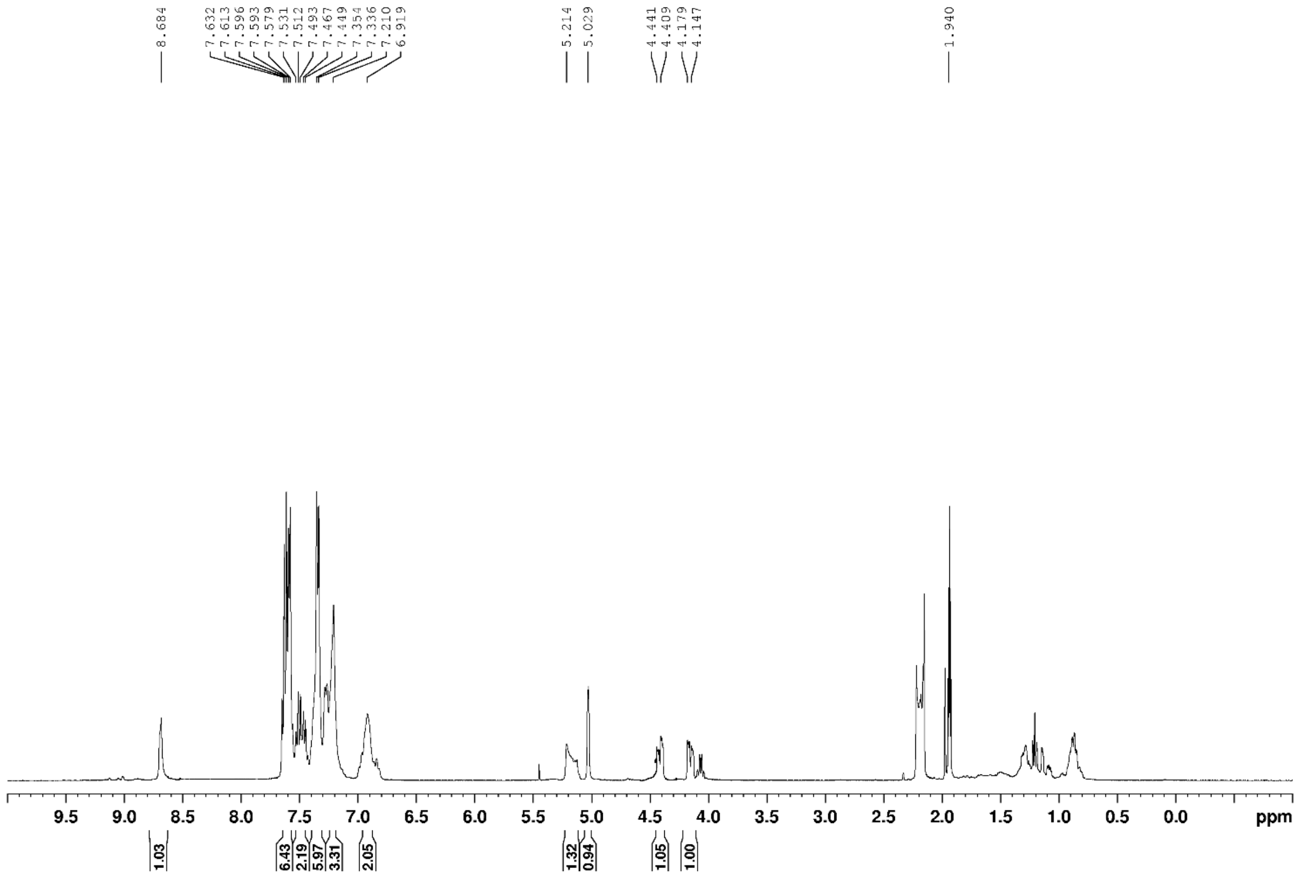

^{13}C NMR spectrum of compound 3t (CD_3CN, 100 MHz)

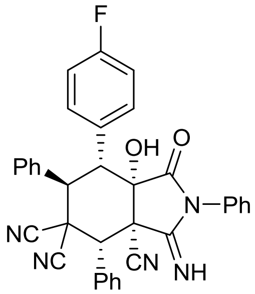

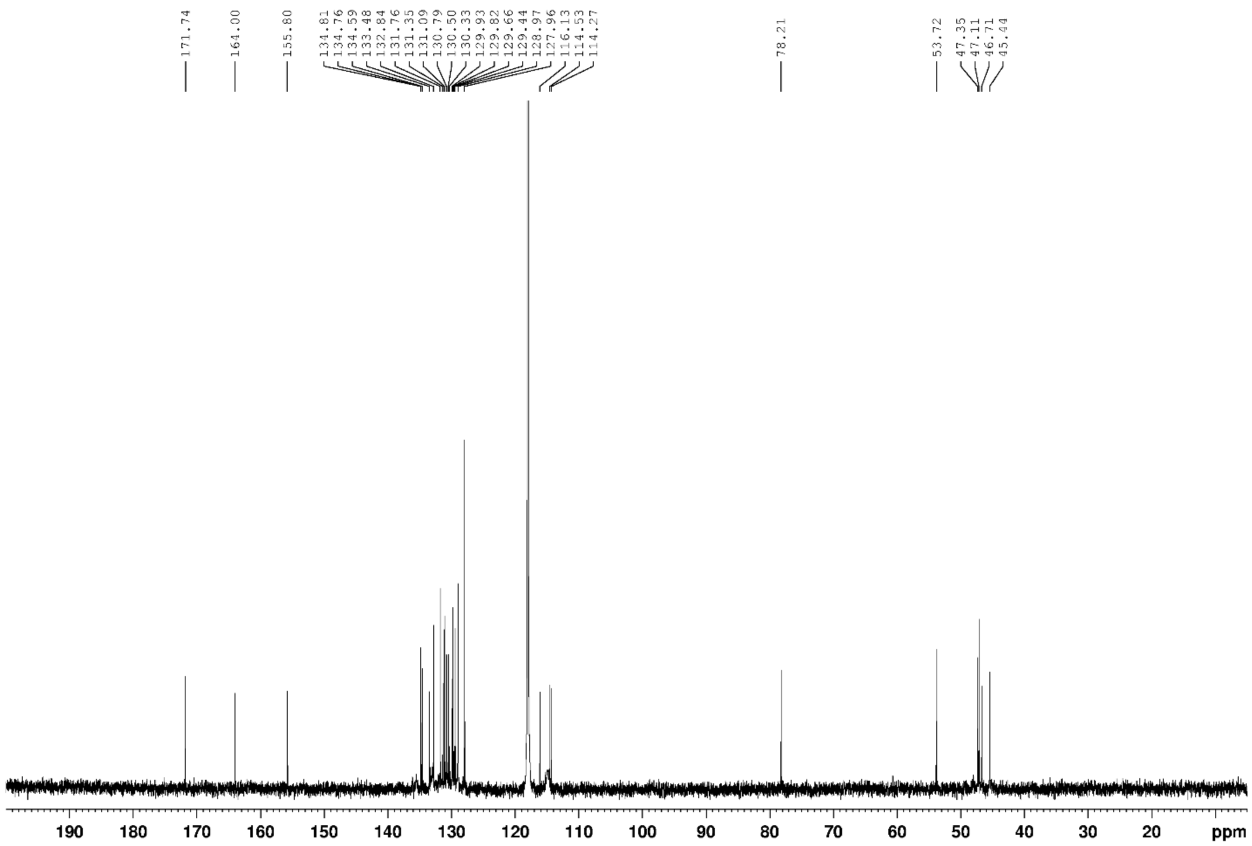

^{1}H NMR spectrum of compound 4a (CD_3CN, 400 MHz)

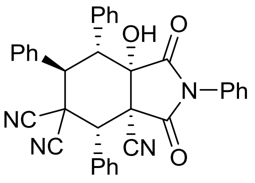

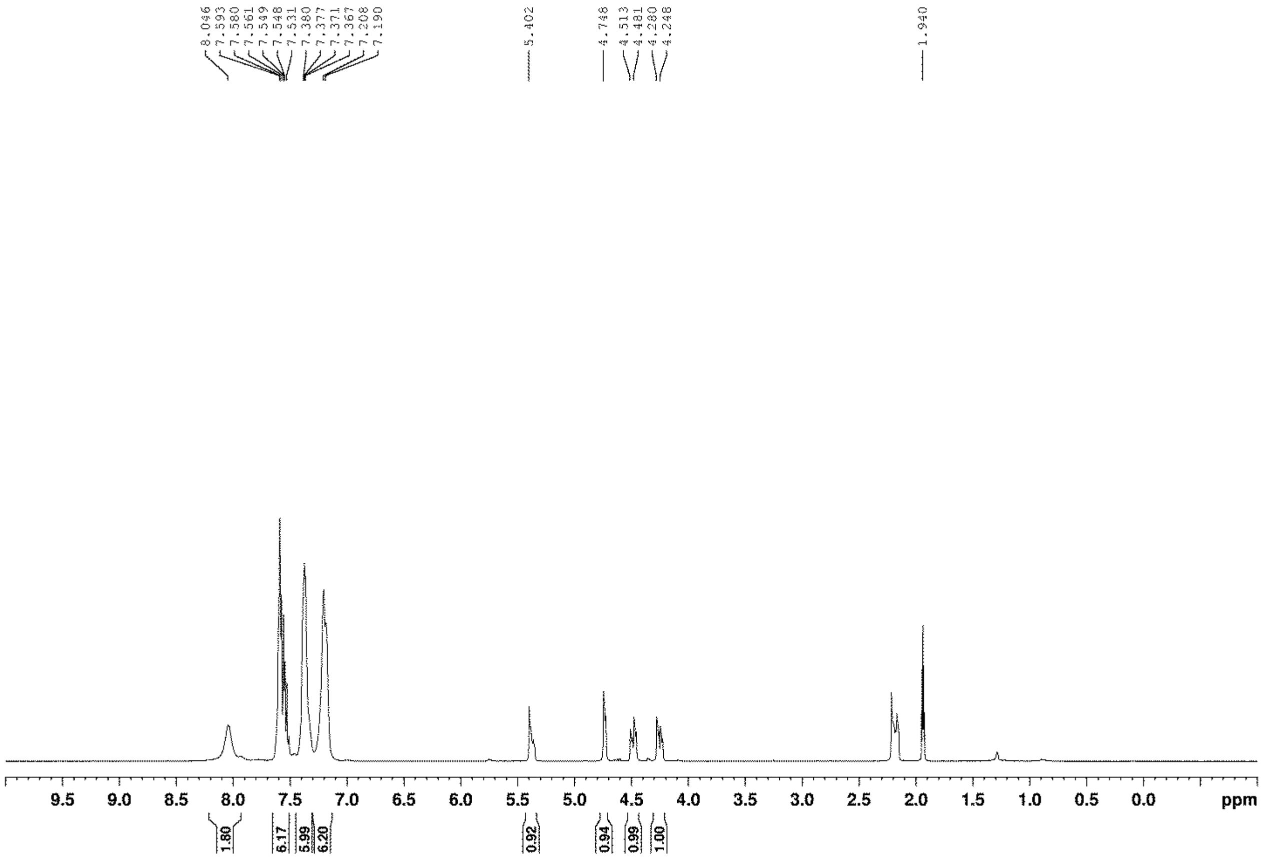

^{13}C NMR spectrum of compound 4a (CD_3CN, 100 MHz)

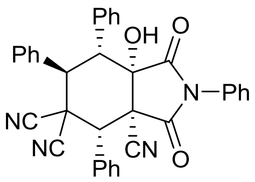

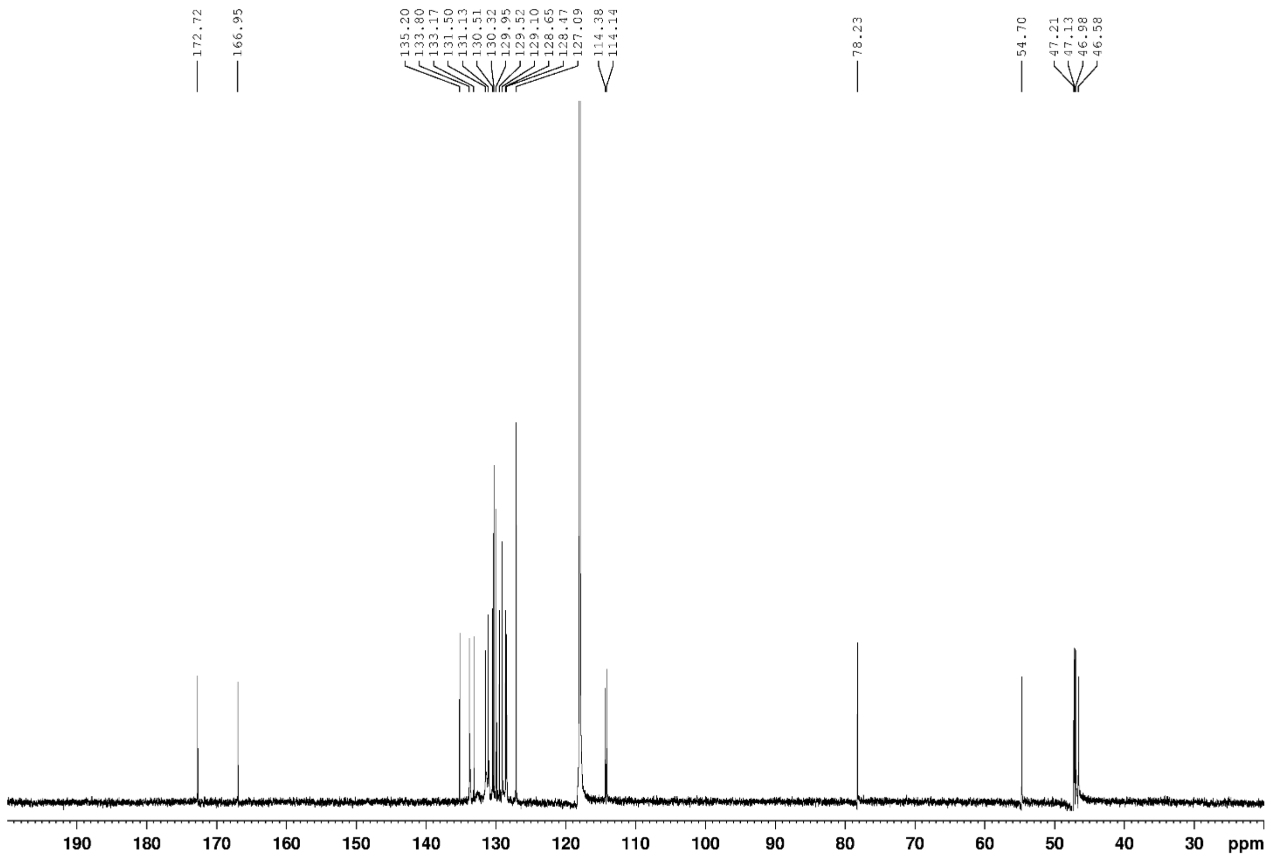

XRD spectra

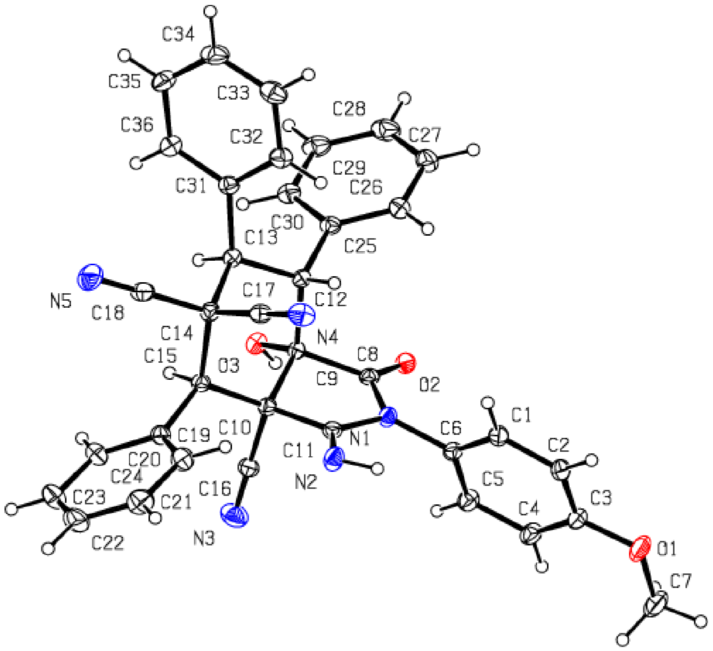

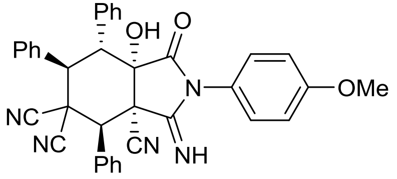

3P, minor

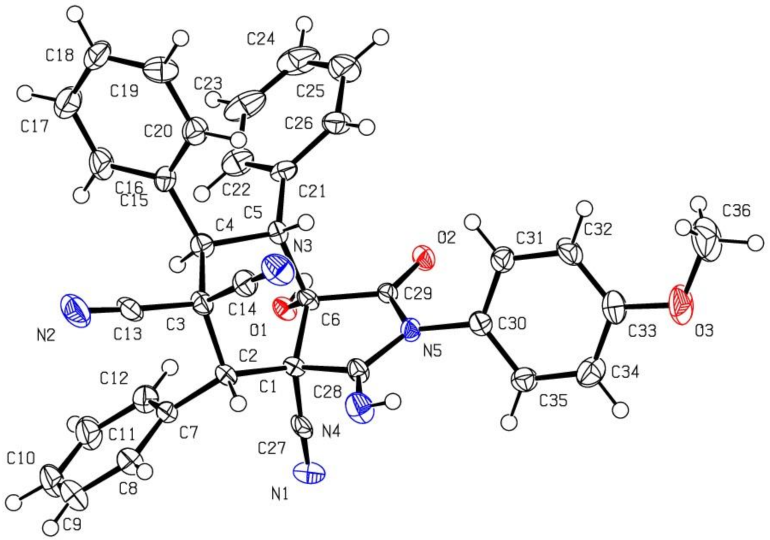

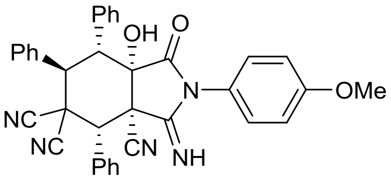

3P, major

CCDC 1404942 (3p, minor) and CCDC 1409146 (3p, major) contain the supplementary crystallographic data for this paper. These data can be obtained free of charge from The Cambridge Crystallographic Data Centre via www.ccdc.cam.ac.uk/data_request/cif.
